# Acoustic-Emergent Phonology in the Amplitude Envelope of Child-Directed Speech

**DOI:** 10.1371/journal.pone.0144411

**Published:** 2015-12-07

**Authors:** Victoria Leong, Usha Goswami

**Affiliations:** Centre for Neuroscience in Education, Department of Psychology, University of Cambridge, Cambridge, United Kingdom; Birkbeck College, UNITED KINGDOM

## Abstract

When acquiring language, young children may use *acoustic* spectro-temporal patterns in speech to derive *phonological* units in spoken language (e.g., prosodic stress patterns, syllables, phonemes). Children appear to learn acoustic-phonological mappings rapidly, without direct instruction, yet the underlying developmental mechanisms remain unclear. Across different languages, a relationship between amplitude envelope sensitivity and phonological development has been found, suggesting that children may make use of amplitude modulation (AM) patterns within the envelope to develop a phonological system. Here we present the Spectral Amplitude Modulation Phase Hierarchy (S-AMPH) model, a set of algorithms for deriving the dominant AM patterns in child-directed speech (CDS). Using Principal Components Analysis, we show that rhythmic CDS contains an AM hierarchy comprising 3 core modulation timescales. These timescales correspond to key phonological units: prosodic stress (Stress AM, ~2 Hz), syllables (Syllable AM, ~5 Hz) and onset-rime units (Phoneme AM, ~20 Hz). We argue that these AM patterns could *in principle* be used by naïve listeners to compute acoustic-phonological mappings without lexical knowledge. We then demonstrate that the modulation statistics within this AM hierarchy indeed parse the speech signal into a primitive hierarchically-organised phonological system comprising stress feet (proto-words), syllables and onset-rime units. We apply the S-AMPH model to two other CDS corpora, one spontaneous and one deliberately-timed. The model accurately identified 72–82% (freely-read CDS) and 90–98% (rhythmically-regular CDS) stress patterns, syllables and onset-rime units. This in-principle demonstration that primitive phonology can be extracted from speech AMs is termed Acoustic-Emergent Phonology (AEP) theory. AEP theory provides a set of methods for examining how early phonological development is shaped by the temporal modulation structure of speech across languages. The S-AMPH model reveals a crucial developmental role for *stress feet* (AMs ~2 Hz). Stress feet underpin different linguistic rhythm typologies, and speech rhythm underpins language acquisition by infants in all languages.

## Introduction

A necessary step in language acquisition is the development of a phonological system, implicit knowledge about the inventory of the sound system of a language. In young children, there is also growing explicit *awareness* of phonological structure as language acquisition proceeds. This explicit phonological awareness may be defined as the ability to detect or manipulate any phonological unit within a word, for example phoneme, rime, or syllable. Explicit phonological awareness develops in an hierarchical fashion from large to small units across languages. Awareness of large phonological units (e.g. syllables and rimes) develops spontaneously prior to literacy tuition, whereas awareness of the smallest sound elements in words (phonemes) depends on tuition in an alphabetic orthography [1]. For example, by 2–3 years of age, children can count the number of syllables in a word, and say whether two words rhyme [2]. These are deceptively complex feats of speech engineering, in principle requiring the child to utilise the overt *acoustic* spectro-temporal structure of the speech signal in order to discover the latent *phonological* building blocks of the English language. These building blocks can be viewed as a nested hierarchy of prosodic stress patterns, syllables and onset-rime units.

The acoustic bases of phonological awareness remain under debate, however children with impaired phonological development (children with developmental dyslexia) show reduced sensitivity to the amplitude modulation (AM) structure of acoustic signals across languages (e.g., English, French, Spanish, Hungarian, Dutch, Chinese, Finnish; [3–8]). In each language studied to date, individual differences in sensitivity to acoustic cues to AM (as measured by children's psychophysical thresholds for detecting differences in amplitude envelope “rise times” or time to the AM peaks) is related to individual differences in phonological awareness. This relationship is found at multiple linguistic levels (e.g., rise time sensitivity is related to prosodic awareness, syllable awareness, rhyme awareness, Chinese tone awareness, phoneme awareness; see [4,8] for recent summaries). These psychophysical data suggest that the ability to extract information about phonological structure from energy patterns in the amplitude envelope (i.e. the slow-varying intensity profile of the signal) could be important for phonological development, across languages. However, it is unclear which particular aspects of speech spectro-temporal modulation architecture (e.g. which modulation timescales and statistics) are the most important for transmitting acoustic information relating to the phonological information that children require to build a language system. The ability to utilise key structural components of the acoustic signal would be particularly crucial for infants, who must build a phonological system from the speech signal ground-up, without the support of pre-existing lexical knowledge.

To investigate in detail how acoustic spectro-temporal structure may be related to linguistic phonological structure, we here apply a Principle Component Analysis (PCA) approach, enabling us to examine the core spectro-temporal dimensions and statistics of the amplitude envelope (AE) of English as spoken to a young child. The AE contains a range of AM patterns across acoustic frequencies and temporal rates. PCA finds orthogonal, uncorrelated components that explain part of the observed variance, and can be used to infer the major spectral frequencies and temporal rates that contribute most to the energy of the CDS signal. We build on modelling by Turner [9] and McDermott & Simoncelli [10] which has demonstrated that natural sounds such as rain, fire, birdsong and speech can be described by statistics such as AM depth, major modulation timescales and cross-frequency-channel modulation dependency. Turner ([9], p. 89–91) noted that the modulation structure of *biological* signals (like speech and birdsong) are characterised by particularly strong correlations across frequency and time. We used PCA to examine whether the rich and highly inter-correlated spectro-temporal patterning in one corpus of child-directed speech (CDS, nursery rhymes) could be captured by a core, low-dimensional, modulation architecture that a naive listener could then exploit to extract phonological patterns. As well as being rhythmically exaggerated, CDS contains simpler syntactic structures [11], shorter sentences [12], and concerns topics that are of interest to the child [13–14]. Thus, CDS provides optimally-structured input for children's extraction of phonological structure. The results of this PCA provided the basic architecture for our Spectral-Amplitude Modulation Phase Hierarchy Model (S-AMPH). Our model uses the core spectro-temporal modulation patterning of CDS (an AM hierarchy) to perform acoustic-phonological *structure-mapping*. Key modulation statistics within the AM hierarchy (such as maxima and oscillatory phase-relationships) are utilised to make mappings between acoustic structure and phonological structure at the prosodic, syllable and onset-rime levels.

To explore the utility of these structure-mappings, we then applied the S-AMPH model to two corpora of nursery rhymes, one rhythmically-timed and one spontaneous (non-timed). The automatic computations of the model enabled us to assess the efficacy of modulation-based (signal-based) phonological parsing by the naive listener (i.e. without prior lexical knowledge). Studies of phonological development in children suggest that the deliberate use of *rhythmically-timed* speech plays a critical role developmentally [2]. Rhythmically-timed speech occurs naturally in the language games, nursery rhymes, singing and playground activities (e.g., clapping games) of children [15], and in caretaking activities with infants and toddlers (e.g., singing lullabies, language routines [e.g. during feeding or bouncing on the lap] and Peek-a-Boo games). When nursery rhymes are spoken spontaneously (rather than sung to a musical beat), there is little apparent loss of prosodic expression. In spoken mode, speakers are free to vary syllable and phrase durations, speaking tempo, and even metrical interpretation. Nevertheless, given that most nursery rhymes are perfect metrical poems, it is plausible that strong rhythmic cues are still present. By using two corpora of CDS, we were able to assess how well the S-AMPH algorithms extracted phonological structure in more optimal (rhythmically-timed) and more spontaneous speaking conditions.

### The Amplitude Envelope

In signal processing terms, the speech signal can be expressed as the product of the slow-varying amplitude envelope (which contains the AM patterns) and the quickly-varying fine structure (which contains frequency modulation [FM] patterns, see [16–17]). In general, AM patterns are perceived as fluctuations in signal loudness (at slow rates of modulation), or as rhythm and texture (at faster rates of modulation) [9–10], whereas FM patterns are perceived as fluctuations in pitch. It is possible to separate the AM-bearing amplitude envelope of speech from its fine structure by a process of demodulation, the approach adopted here [18–20]. Speech sounds do not contain equal energy over all acoustic frequencies at all points in time, thus the envelope for low-frequency components (e.g. ~500 Hz, the carrier used in most dyslexia studies of AE rise time discrimination, see [4]) will typically be very different from the envelope for high-frequency components (e.g. ~5000 Hz). Similarly, speech sounds do not contain equal energy across all modulation rates (timescales). In adult-directed speech (ADS), the modulation spectrum peaks at around 3–5 Hz, the syllable rate (as adults typically produce around 5 syllables per second, see [21–22]). In speech to very young children (i.e. under 2 years of age), by contrast, the modulation spectrum peaks at ~ 2 Hz [23]. This peak at the prosodic rate [24] suggests that different acoustic information is foregrounded in CDS compared to ADS, with slower modulations (~2 Hz) more dominant in the speech used when speaking to very young children. The shifted modulation peak suggests that 2 Hz AMs may form an important acoustic substrate for language acquisition.

Slower AMs at around 2 Hz contribute to the perception of speech rhythm and prosodic structure [25]. For example, we have shown that adult listeners' perception of the strong metrical patterns in children's nursery rhymes (patterns of alternating strong and weak syllables, as in the nursery rhyme ‘*Mary Mary Quite Contrary’*, hereafter referred to as 'Mary Mary') is determined by the relative phase patterning of slow stress-rate (~2 Hz) AMs and syllable-rate (~4 Hz) AMs in the wholeband envelope of the speech signal [25]. In many children's nursery rhymes strong syllables occur approximately every other syllable [26], and indeed across languages, adults produce stressed syllables approximately twice per second, corresponding to a rate of 2 Hz [27]. Here we assess the modulation architecture of a range of types of nursery rhyme, covering trochaic patterns (strong-weak syllable alternation, as in ‘*Mary Mary’*), iambic patterns (weak-strong syllable alternation, as in the nursery rhyme ‘*The Queen of Hearts she made some tarts’*) and dactyl patterns (strong-weak-weak syllable patterning, as in the nursery rhyme '*Pussycat Pussycat Where Have You Been*?'). Developmentally, extraction of a phonological system takes several years, and is facilitated by a rich set of social learning mechanisms [28–31]. Here we consider the potential contribution of the modulation statistics of the speech signal alone. We estimate the efficacy of signal-based acoustic-phonological structure-mapping by a naive learner using our S-AMPH model.

### Potential Links to Neuronal Oscillatory Processing of Speech

Our signal-based approach draws upon a convergent area of research regarding the *neural* basis of speech processing. Auditory neuroscience studies show that speech encoding may be related to the entrainment of oscillatory cortical networks [32–35]. Populations of cortical neurons are known to fluctuate in excitability at certain endogenous temporal rates. These oscillations can be divided into frequency bands, including delta (0.5–4 Hz), theta (4–8 Hz), alpha (8–12 Hz), beta (12–30 Hz) and gamma (30–80 Hz). Amongst these, delta, theta and gamma oscillations are those most frequently linked to speech perception in experimental studies. For example, Poeppel and others [32,36] have argued for the central importance of theta band oscillators in encoding syllable-level information in ADS, while gamma band oscillators are thought to be important for encoding phonetic information. Goswami [4,8] argued that the neural delta band (0.5–4 Hz) might also be important developmentally, perhaps for encoding metrical rhythmic structure (information about syllable stress and prosodic phrasing). Neuronal oscillations in the auditory cortex entrain to both amplitude and frequency modulation patterns in auditory input. The entrained neural response can be measured using electroenecephalography (EEG) or magnetoencephalography (MEG), and is generated in response to AM patterns in speech [33,37–38] as well as to AM-modulated noise [39]. Furthermore, phase-locked components in the neural response to ADS (particularly in the theta band, 4–8 Hz) are correlated with speech comprehension and intelligibility [33,37,40]. Cortical oscillations are hierarchically-nested [35,41], for example delta phase modulates theta phase, and theta phase modulates gamma power. This oscillatory hierarchy provides an ideal neural mechanism for multi-timescale encoding and parsing of the speech signal [33,34,36]. Indeed, Gross et al [35] demonstrated that the neuronal oscillatory hierarchy can entrain to an equivalent hierarchy of acoustic modulation patterns in ADS, enabling each tier of the hierarchy to parse acoustic information relevant to phonological patterning at different timescales (i.e. delta-stress patterning, theta-syllable patterning, gamma-phonetic patterning).

Nevertheless, there is still considerable debate around the potential functional role(s) of cortical entrainment to speech and whether the neural tracking of the speech envelope is related to intrinsic neural oscillations [42]. Cortical entrainment has been implicated in the encoding of acoustic features, in the parsing of boundaries between syllables, and in the selection of sensory information in complex listening environments [42]. Despite these demonstrations, to date it is not known whether an acoustic hierarchy of modulation patterns exists in the speech signal that matches key neural timescales. Also unexplored is the efficacy of such a neuro-acoustic model for real-life speech processing. For example, one criticism of multi-timescale models has been that natural speech is not a highly rhythmic signal, and hence the extent to which a rhythmically-regular parsing mechanism (neuronal oscillations) could capture real-life patterns of uttered syllables is doubtful [43].

Accordingly, one major aim of the current modelling is to understand the core spectro-temporal patterning in CDS, a natural register used with children which does exhibit strong rhythmic patterning. Indeed, the English nursery rhyme deliberately uses a range of metrical patterning (e.g. trochaic [Strong-weak], iambic [weak-Strong], dactyl [Strong-weak-weak]) and children find these different rhythmic patterns very enjoyable. Although we do not assess the related question of neural efficacy here, it is plausible that the rhythms and rhymes of the nursery are developmentally adaptive. Our S-AMPH model therefore utilises oscillatory mechanisms and landmarks such as auditory edges (rise times) to perform acoustic-phonological structure-mapping, and thus provides an *in-principle* source of evidence on the extent to which neuro-acoustic models of speech processing could be successful for parsing highly rhythmic CDS.

We present two related studies, which we preview here. The first is a PCA analysis to uncover the core spectro-temporal dimensions and statistics of the amplitude envelope (AE) of English CDS using a nursery rhyme corpus. This PCA analysis results in the identification of 5 spectral bands and 3 modulation rate bands (as detailed in the first and third tables of the Results section) that provide the basis for the hierarchical modulation architecture that underpins the S-AMPH model. An example of this 5x3 S-AMPH representation of CDS AM patterns is shown in the third figure of the Results section. The second study then goes on to address the potential relationship between acoustic modulation structure (as formalised in the S-AMPH model) and linguistic phonological structure (hierarchical patterns of stress, syllables and onset-rime units). We derive Acoustic-Emergent Phonology theory by using the S-AMPH computational model to perform acoustic-phonological mapping with two nursery rhyme corpora, one timed (Nursery Rhyme Corpus 2) and one spoken spontaneously (Nursery Rhyme Corpus 3, note that this corpus is a subset of the nursery rhymes in Nursery Rhyme Corpus 1). Modulation patterns in the speech signal are used to parse and extract phonological structure automatically, via structure-mapping between the acoustic signal and the phonological hierarchy. We propose several core principles for acoustic-phonological mapping—these include a 1:1 structural relationship between AM cycles and phonological units, and the use of hierarchical AM phase relationships to infer prosodic prominence (see the last figure of the Results section for an illustration). We demonstrate that these automatic mappings enable primitive phonology at stress, syllable and onset-rime levels to be extracted from rhythmic CDS with a high degree of efficiency (see the last table of the Results section).

## Methods

### Ethics Statement

This study was approved by the Cambridge Psychology Research Ethics Committee. Written informed consent was obtained from all participants using a consent form and information sheet approved by the Cambridge Psychology Research Ethics Committee.

### Speech Materials for PCA Analysis

#### Nursery Rhymes Corpus 1

The speech materials used in the PCA analysis were 44 children's nursery rhymes each read in a child-directed manner by 6 different female native speakers of British English, giving a total of 264 spoken samples. The full list of nursery rhymes is provided in S1 Appendix and the speech data are available for download at: http://figshare.com/s/f8012786bda511e48b4906ec4bbcf141. S1 Appendix also provides the metadata for this CDS corpus. The text for the nursery rhymes was compiled from children's books such as 'This Little Puffin' [44] and 'Nursery Treasury' [45]. All 6 female speakers had extensive prior experience in working with young children. Two participants were Cambridge University lecturers in early years education (having previously been teachers), and a further two participants were, at the time, working as early years teachers. One participant was a speech and language therapist working with children. The final participant was an ex-teacher and doctoral student whose research involved working with children using poetry. All the speakers were familiar with the children's nursery rhymes and stories used in this study but were unaware of the goals of the study. To elicit child-directed speech, speakers were prompted with a picture depicting young children of a nursery age (i.e. 3–5 years old). They were told to speak in a lively and engaging manner as if they were conversing with the children in the picture. Each speaker was allowed to speak the nursery rhymes at her own natural pace (rather than speaking in time to a metronome), such that the utterances retained their natural cadence and flow. All speech samples were digitally recorded using a TASCAM digital recorder (44.1 kHz, 24-bit), together with an AKG C1000S condenser microphone.

### Speech Materials for Model Evaluation

#### (a) Timed speech: Nursery Rhyme Corpus 2

The timed child-directed speech was generated by asking 3 native English speakers (1 M, 2 F) to repeat a single nursery rhyme in time to a 3 Hz metronome beat. Four different prosodic templates were used, as shown in Table 1, yielding 12 sentences in total. The nursery rhyme was *"Jack and Jill went up the hill to fetch a pail of water*, *Jack fell down and broke his crown*, *and Jill came tumbling after"*. The speakers were asked to vary the prosodic patterning of the rhyme to reflect common metrical patterns in English (i.e. trochees, iambs, dactyls and amphibrachs). Speakers were allowed to practice each prosodic variation to proficiency before their utterances were recorded. These recordings comprised Nursery Rhyme Corpus 2.

**Table 1:** Prosodic templates for metronome-timed sentences in Corpus 2.

Syllable Stress Pattern (CAPITAL LETTERS = stressed)	Prosodic Foot
JACK and JILL went UP the HILL to FETCH a PAIL of WAter…	Trochaic (Sw)
jack AND jill WENT up THE hill TO fetch A pail OF waTER…	Iambic (wS)
JACK and jill WENT up the HILL to fetch A pail of WAter…	Dactyl (Sww)
jack AND jill went UP the hill TO fetch a PAIL of waTER…	Amphibrach (wSw)

Stressed syllables are shown in capital letters.

#### (b) Untimed speech: Nursery Rhyme Corpus 3

The untimed child-directed speech consisted of 20 nursery rhyme sentences, each produced by 6 female native English speakers, yielding a total of 120 sentences. These sentences comprised Nursery Rhyme Corpus 3. Each nursery rhyme sentence was 24 syllables long, and the 20 nursery rhymes were selected from the larger set of 44 nursery rhymes that were used in the PCA exercise. As shown in Table 2, ten of these sentences were the first lines of bi-syllable footed nursery rhymes (i.e. predominantly trochaic or iambic patterning), and the other ten sentences were the first lines of tri-syllable footed nursery rhymes (i.e. predominantly dactyl or amphibrach patterning). Each nursery rhymes was freely-read by native English speaking early-years practitioners, as if they were speaking to young children. Each speaker was allowed to read the sentences at her own natural pace, rather than speaking in time to a metronome. Consequently her rate of speaking could vary considerably within the same utterance.

**Table 2:** List of 20 untimed nursery rhyme sentences, each 24 syllables long.

Bi-Syllable Foot	Tri-Syllable Foot
1. Old MacDonald had a farm, E-I-E-I-O. And on that farm he had some cows, E-I-E-I	1. Little Miss Muffet sat on a tuffet eating her curds and whey. There came a big spider who sat …
2. Mary had a little lamb its fleece was white as snow. And everywhere that Mary went the lamb was …	2. Little Jack Horner sat in a corner eating his Christmas pie. He stuck in his thumb and pulled out …
3. Polly put the kettle on, Polly put the kettle on, Polly put the kettle on, we'll all have …	3. Little Boy Blue come blow your horn, the sheep's in the meadow the cow's in the corn. Where is the boy who …
4. Yankee Doodle came to London riding on a pony. He stuck a feather in his hat and called …	4. Curly locks, curly locks will you be mine? You shall not wash dishes nor feed the swine, but sit on a…
5. Peter Peter pumpkin eater had a wife and couldn't keep her. Put her in a pumpkin shell and …	5. To market to market to buy a fat pig. Home again home again dancing a jig. To market …
6. Mary Mary quite contrary, how does your garden grow? With silver bells and cockle shells and pre …	6. Pussycat pussycat where have you been? I've been up to London to visit the Queen. Pussycat …
7. Simple Simon met a pieman going to the fair. Says Simple Simon to the pieman, "let me …	7. Ladybird ladybird fly away home, your house is on fire and your children are gone. All except …
8. Lucy Lockett lost her pocket, Kitty Fisher found it. Not a penny was there in it, only …	8. There was an old Lady who swallowed a spider, that wriggled and wiggled and tickled inside her …
9. Cobbler Cobbler mend my shoe, get it done by half past two. Half past two is much too late, get it done …	9. There once were two cats of Kilkenny. Each thought there was one cat too many. So they fought and they fit …
10. Peter Piper picked a peck of pickled peppers. A peck of pickled peppers Peter Piper picked ….	10. Lavender's blue, dilly dilly, lavender's green. When I am king, dilly dilly, you shall be queen …

All timed and untimed speech samples were digitally recorded using a TASCAM digital recorder (44.1 kHz, 24-bit), together with an AKG C1000S condenser microphone. The digital sound file of each sentence was then manually analysed to locate the "ground-truth" location of its syllables, their onset-rime divisions, and to transcribe its prosodic stress patterning. To locate syllables and their onset-rime divisions, the midpoints (for syllable locations) and onsets (for onset-rime divisions) of all syllable vowel nuclei were manually annotated using Praat software [46]. The stress patterns of each sentence (i.e. pattern of 'S' and 'w' syllables) were then manually stress-transcribed by a female native English speaker with formal training in Linguistics (not the authors). Stress transcription was done by listening to each sentence carefully, and judging whether each syllable sounded stressed or unstressed. The hypotheses of the current study were not disclosed to the individual doing the transcription so that her judgments would be unbiased.

Table 3 shows the key acoustic parameters for the three CDS corpora. For comparison, the same acoustic parameters were also measured for a corpus of adult-directed speech that was produced by the 6 female speakers of CDS Corpora 1 & 3 (see S4 Appendix for ADS description). Consistent with the prior literature [28–29], all 3 CDS corpora have a higher mean and maximum intensity than ADS. For the pitch measures, both female-only CDS corpora (1 & 3) were higher in mean pitch than the female-only ADS corpus as expected. However, the mixed CDS corpus 2 (1M, 2F speakers) had a similar mean pitch to the female-only ADS corpus, due to the lower fundamental frequency of the male speaker. CDS corpus 1 (the stimulus set used in PCA analysis for model derivation) was also higher in maximum pitch than the ADS sample, but the other CDS corpora showed a lower maximum pitch than the ADS sample.

**Table 3:** Acoustic parameters for the three CDS corpora used for PCA analysis (Corpus 1) and for model evaluation (Corpora 2 & 3). Each cell shows the mean value across all speakers, with the standard deviation across speakers in brackets below. Note that the speakers of Corpora 1 & 3 are all female, but Corpus 2 included 1 male speaker.

Corpus	Max Intensity (dB SPL)	Mean Intensity (dB SPL)	Max Pitch (Hz)	Mean Pitch (Hz)
**CDS Corpus 1** (n = 6, all F)	79.4 (4.7)	57.0 (4.7)	470.9 (34.6)	200.4 (12.4)
**CDS Corpus 2** (n = 3, 1M 2F)	83.3 (3.2)	61.1 (2.5)	394.1 (46.1)	168.8 (26.3)
**CDS Corpus 3** (n = 6, all F)	76.0 (4.6)	59.6 (4.7)	409.4 (30.1)	203.4 (11.9)
**ADS** (n = 6, all F)	75.7 (6.0)	55.3 (5.5)	427.6 (30.8)	169.0 (8.5)

This acoustic analysis confirms that the CDS corpora showed the appropriate acoustic hallmarks of child-directedness [28–29], and that intensity (the key acoustic parameter being modelled here) was appropriately and consistently modulated in our speakers' utterances.

### Signal Pre-Processing

The purpose of signal pre-processing was to generate high-dimensional representations of the AM structure of each speech sample in the spectral and temporal domains respectively. These high-dimensional representations form the substrate for later dimensionality reduction by PCA, whose purpose is to infer the underlying core modulation architecture of the signal. For example, in the spectral domain, 28 dimensions are generated by pre-processing, corresponding to the time-varying AM patterns in each of 28 finely-spaced acoustic frequency bands spanning 100–7250 Hz. In the temporal domain, 24 dimensions are generated by pre-processing, corresponding to the time-varying AM patterns at different oscillatory rates from 0.9–40 Hz.

#### (a) Generation of High-Dimensional Representation of Spectral Modulation

**Fig 1:**
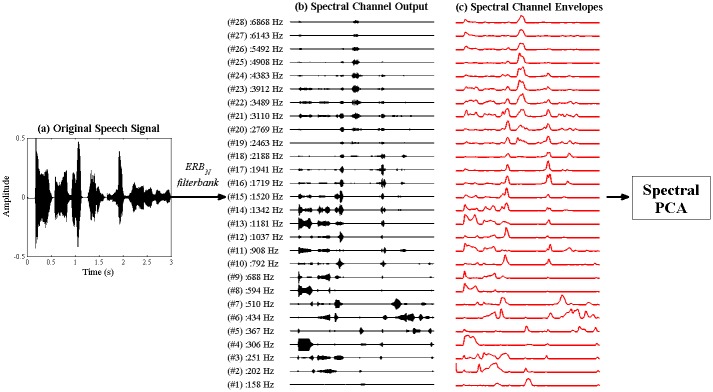
Signal processing steps prior to Spectral PCA. The original acoustic signal (a) is passed through an ERB_N_-spaced filterbank, yielding a set of high-dimensional spectral channel outputs (b). The envelope is extracted from each spectral channel output using the Hilbert transform (c), and these envelopes are entered into the spectral PCA to identify patterns of covariation across spectral channels.

As shown in Fig 1, a high-dimensional representation of the spectral modulation patterns in the speech signal was constructed in 2 steps. First, the raw acoustic signal was passed through a finely-spaced ERB_N_ (equivalent rectangular bandwidth) spectral filterbank that mirrored the frequency decomposition that is performed at the cochlea of a normal human listener [47–48]. This filtering step effectively split the speech signal into 28 log-spaced audio frequency channels spanning 137–7250 Hz. The parameters and frequency response characteristics of the ERB_N_ spectral filterbank are provided in S2 Appendix. In the next step, the Hilbert envelope was extracted from each of the 28 channel outputs of the spectral filterbank, and low-pass filtered under 40 Hz.

**Fig 2:**
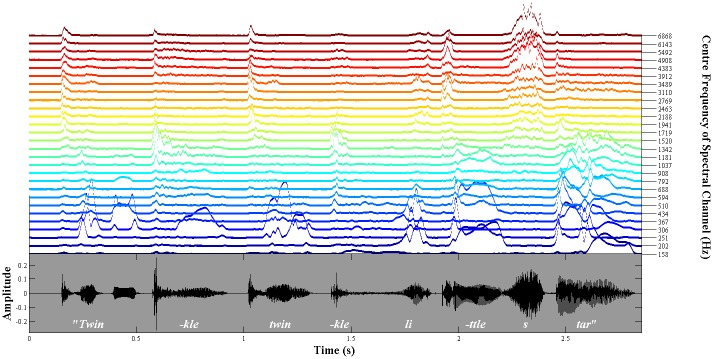
28 spectral-channel (high-dimensional) envelope representation of the nursery rhyme sentence "Twinkle twinkle little star". The bottom panel (grey) shows the original sound pressure waveform of the sentence, where the x-axis indicates time and the y-axis indicates signal amplitude. The top panel shows the corresponding high-dimensional set of 40 Hz low-pass filtered Hilbert envelopes. The envelopes for the 28 ERB_N_-spaced spectral channels are plotted upwards in order of increasing acoustic frequency, where each coloured line indicates a different spectral channel (i.e. blue = low frequency, red = high frequency). The vertical height of each coloured line indicates the amplitude of the envelope at each point in time.

Fig 2 illustrates the resulting high-dimensional (i.e. 28 channel) set of envelopes from each spectral band for the nursery rhyme sentence *"Twinkle twinkle little star"*. From Fig 2, it may be observed that certain groups of spectral channels tend to show similar patterns of activation over time. For example, the high-frequency channels, plotted in red (~ above 4000 Hz), all show markedly increased activation corresponding to the fricative /s/ in the word "star" (at ~2.3s). Conversely, the energy in the vowel sound /a/ in "star" (at ~2.5s) predominantly activates a group of low-mid frequency channels plotted in blue (~below 1000 Hz). This similarity in the pattern of co-activation across groups of spectral channels supports the view that there is significant *redundancy* in this high-dimensional spectral representation of the speech signal. That is, the major patterns of activation elicited by speech sounds could be adequately captured by a smaller number of more widely-spaced spectral *bands*, which are designed to reflect the core spectral content of different speech sounds. So, in the previous example, the 6 spectral channels (CFs of 3912–6868 Hz) that were co-activated by the /s/ sound could theoretically be replaced by a single spectral band spanning their combined bandwidth, whose activation pattern would then be the weighted sum of the original 6 channels. The aim of the first spectral dimensionality reduction (PCA) exercise is to identify the appropriate number and spacing of this smaller set of non-redundant spectral bands, using co-modulation in the high-dimensional ERB_N_ representation as the basis.

#### (b) Generation of High Dimensional Representation of Temporal (Rate) Modulation

**Fig 3:**
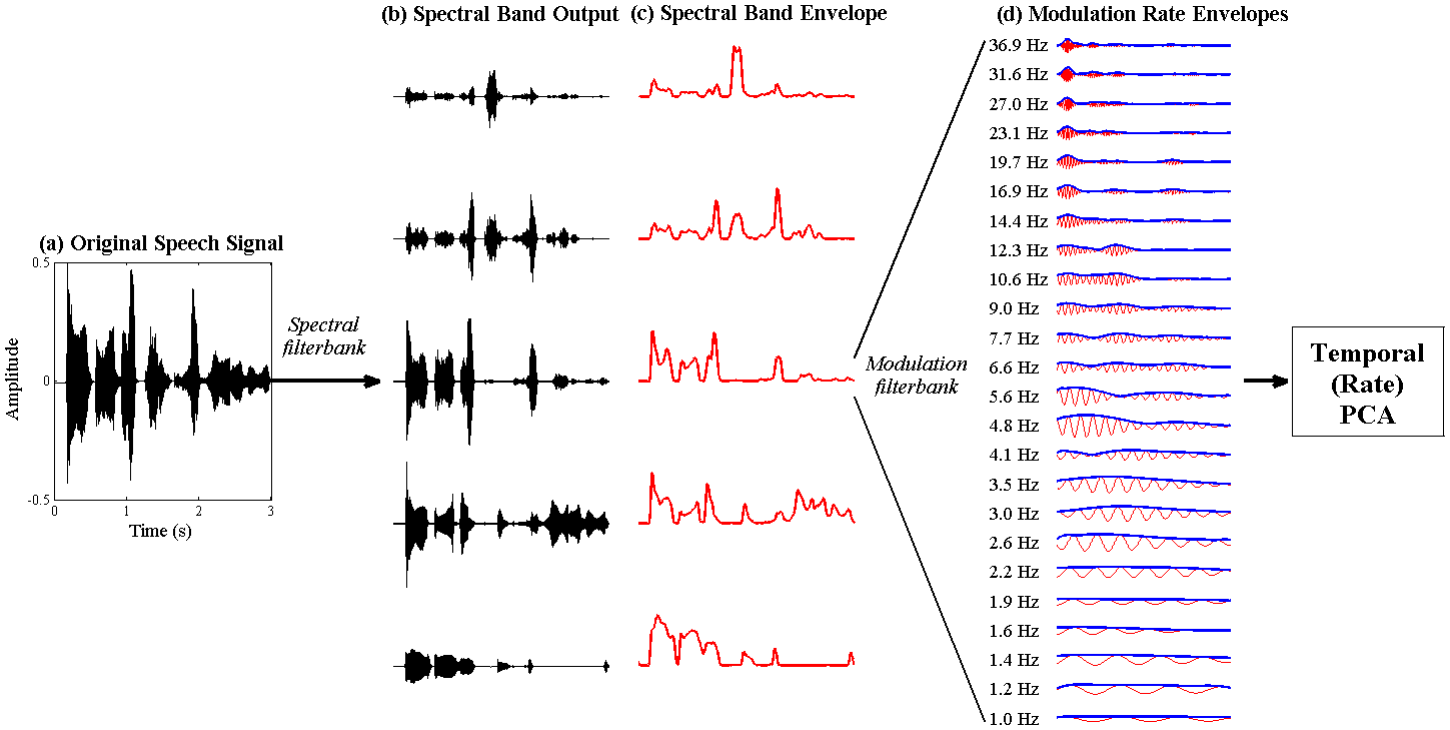
Signal processing steps prior to Temporal (rate) PCA. The original acoustic signal (a) is passed through a low-dimensional spectral filterbank, yielding a small set of core spectral band outputs, (b). The parameters of the low-dimensional spectral filterbank were determined in the prior Spectral PCA procedure. The envelope, (c), is extracted from each spectral band output using the Hilbert transform. Each envelope is further passed through a high-dimensional modulation filterbank, yielding a set of high-dimensional modulation rate envelopes, (d). This rate-filtering is performed for each spectral band envelope, but for simplicity, only the modulation rate envelopes from a single spectral band are shown in this figure. Finally, the power profiles of the modulation rate envelopes (bold blue line) are entered into a temporal (rate) PCA to identify patterns of covariation across modulation rates.

Applying a similar logic to the temporal modulation *rate* domain, patterns of co-activation can be identified from a high-dimensional representation of speech modulations at different temporal rates, and used to derive a smaller number of modulation rate *bands* that characterise the major *timescales* at which speech sounds vary. The high-dimensional representation of modulation rate patterns in speech was constructed in 3 steps (see Fig 3). First, the raw acoustic signal was filtered into the small number (5) of spectral bands that were identified in the spectral PCA analysis. Next, the Hilbert envelope was obtained for each of these spectral bands. Finally, the 5 spectral band envelopes were each passed through a finely log-spaced, 24-channel modulation filterbank, spanning 0.9–40 Hz, resulting in 5 sets of 24 modulation rate-filtered envelopes. The parameters of the modulation filterbank are detailed in S2 Appendix. The PCA analysis to identify patterns of co-activation across modulation rate channels was then conducted separately for *each* of the 5 spectral bands. For this PCA exercise, only the *power* of the modulation-rate envelopes was used. This was because the rate-filtered envelopes were effectively sinusoids at different frequencies whose oscillatory phases were not aligned in time, and therefore had an artificially low correlation to each other (on average <0.1). When the envelopes were entered whole into a PCA analysis, this low correlation across modulation rates meant that the first few principal components were weak, only accounting for ~35% of the total variance in the data. Since we were primarily interested in patterns of co-activation (i.e. changes in power) across different modulation rates, rather than in patterns of temporal synchronisation (i.e. alignments in phase), phase information was discarded, and only the power of the modulation-rate envelopes was used in this PCA analysis.

### Principal Components Analysis (PCA) to Derive the Spectro-Temporal Banding Structure of the S-AMPH Model

A separate principal components analysis was conducted for each speech sample in Nursery Rhyme Corpus 1 (44 nursery rhymes x 6 speakers), taking the 28 spectral channels or 24 modulation rate channels as variables, and the individual datapoints in the filtered envelopes (downsampled to 1050 Hz) as observations. It should be noted that the filtered spectral and temporal modulation envelopes are time series data, and could contain strong temporal dependencies. Therefore, individual datapoints may not be truly independent observations, as assumed by the PCA procedure. That is, the value of the observed data, *x*, at timepoint *t* (i.e. x_t_) is correlated to some degree to its value at the preceding timepoint (x _t-1_), as well as to the *n* timepoints before (x _t-n_). In general, the strength of this temporal correlation decreases as the lag (the value of *n*) between the two timepoints increases. However, Jolliffe [49] notes that *"when the main objective of PCA is descriptive*, *not inferential*, *complications such as non-independence do not seriously affect this objective"* (p.299), and often, no special account is taken of dependence between observations. In the current analyses, our aim was descriptive rather than inferential—we sought to identify major patterns of covariation across spectral or modulation rate channels, rather than to make precise statistical comparisons of the eigenvalues or eigenvectors of the resulting principal components. Indeed, in a preliminary spectral PCA analysis, we found that the loading patterns we obtained were broadly similar whether we used all datapoints (separated by ~1 ms), or only a subset of datapoints that were separated by 250 ms or by 500 ms (and therefore had a lower temporal correlation). Thus, for the current analysis, we included all envelope datapoints and did not perform any correction for temporal dependency. In each Spectral PCA, the 28 spectral channels were entered as separate variables, and the results of the PCA yielded a total of 28 principal components. Only the top 5 principal components were considered in the further analysis of loading patterns (described later), as these cumulatively accounted (on average) for over 65% of the total variance in the original signal. For each modulation rate PCA, the 24 modulation-rate channels were taken as separate variables. Moreover, each of the 5 spectral bands was also analysed separately (i.e. 5 PCAs were conducted per sample). Each PCA yielded a total of 24 principal components, of which only the top 3 were considered for further analysis, as these already cumulatively accounted (on average) for over 70% of the total variance in the original signal.

#### (a) Rectification of PC Loading Patterns

Each principal component (PC) had a different loading pattern across the 28 (or 24) entered variables. For this exercise, only the *pattern* of loadings was important and not the sign (positive or negative). Therefore, the *absolute* PC loading values were used instead of the raw values. This 'rectification' of the component loadings was done to avoid mutual cancellation in the subsequent averaging process. This cancellation would occur when the loading pattern had an opposite valence across samples. In fact, a switch in the valence of PC loadings can be created simply by rotating the eigenvector basis matrix by 180 degrees, which would not change the variance explained (eigenvalues). As such, the +/- signs in component loadings are in effect arbitrary, and the relative *pattern* of loading across channels is more important. The resulting rectified (absolute valued) loading functions allow channel clustering patterns to be observed, but do not allow the computation of principal component *scores* (which were not used in this analysis). The rectified loading patterns for each PC were averaged across all samples, resulting in grand average loading patterns for each PC, which were used in the next step of the analysis.

#### (b) Identification of peaks and troughs in PC loading

In the next step of the PC analysis, peaks and troughs in the grand average rectified PC loading patterns were identified by means of an automated peak-detection procedure in MATLAB ([50], 'findpeaks.m', see S1 Code for formal description). This function identifies local maxima (peaks) of the input data, defined as a data sample that is larger than either of its neighbouring samples. Troughs were detected by first inverting the loading pattern (turning troughs to peaks) and then applying the peak detection procedure to this inverted pattern. As we wanted to detect ALL the peaks and troughs that were present in an unbiased manner, no minimum threshold for peak height was set. However, a minimum peak-to-peak distance was set to ensure that there would be an adequate spacing between the resulting inferred spectral or modulation rate bands. For the Spectral PCA, a minimum peak-to-peak distance of 2 channels was used. For the Temporal (rate) PCA, a larger peak-to-peak distance of 5 channels was used as less frequent peaks/troughs were observed in the loading pattern as compared to the Spectral PCA. Peaks in the rectified loading pattern correspond to clusters of channels that collectively load strongly onto a given principal component due to the strong co-modulation of their envelopes. We took these peaks to indicate the core spectral regions and modulation timescales that characterise speech sounds in our sample of child-directed speech. Troughs in the rectified loading pattern were also of interest because they indicate *boundaries* between co-modulated groups of channels, and therefore mark the edges of the wider, non-redundant modulation bands that we sought to identify in this dimensionality-reduction exercise. As these were troughs in the *rectified* loading patterns, they could have arisen from sign changes in the original loading patterns (e.g. crossings from positive→negative or negative→positive that become inflections after rectification), or else from local drops in loading strength. Either way, troughs are consistent with the abrupt change due to a transition from a spectral/rate region where all the channels carry similar speech information, to one where different speech information is being transmitted (e.g., voicing or frication).

#### (c) Determination of spectral and modulation rate banding

After all the peaks and troughs in the rectified loading pattern had been identified for each of the top 5 (spectral) or 3 (modulation rate) PCs, the following set of criteria were applied to assess the location of spectral and modulation rate bands respectively:

- A spectral band was deemed to be present in a region where at least 2 of the first 5 PCs showed a peak.
- A modulation rate band was deemed to be present in a region where at least 1 of the first 3 PCs showed a peak, for at least 2 out of the 5 spectral bands.

The exact locations of boundary edges between spectral or modulation rate bands were determined on the basis of the most consistent locations of flanking troughs for each group of PC peaks that indicated the presence of a band. For example (see first figure in Results section), for spectral PCA, if 3 of the 5 PCs all showed peaks at around 2500 Hz, this was taken to indicate the presence of a spectral band in the region of 2500 Hz. The upper and lower boundaries of this spectral band were then determined with reference to the flanking (adjacent) troughs for each of the 3 relevant PC peaks. If the flanking troughs for all relevant PCs were located at the same location (e.g. 3900 Hz), this was taken as a clear indication that the upper boundary of the spectral band was located at 3900 Hz. However, if there was a discrepancy between the 3 PC trough locations (e.g. 2 PCs showed a trough at 3900 Hz, but 1 PC showed a trough at 3000 Hz), then the location with the majority of troughs would be used (i.e. 3900 Hz). So boundaries were determined using the most consistent locations across all PCs.

### The S-AMPH Computational Model for Acoustic-Phonological Mapping 1: Finding Syllables

**Fig 4:**
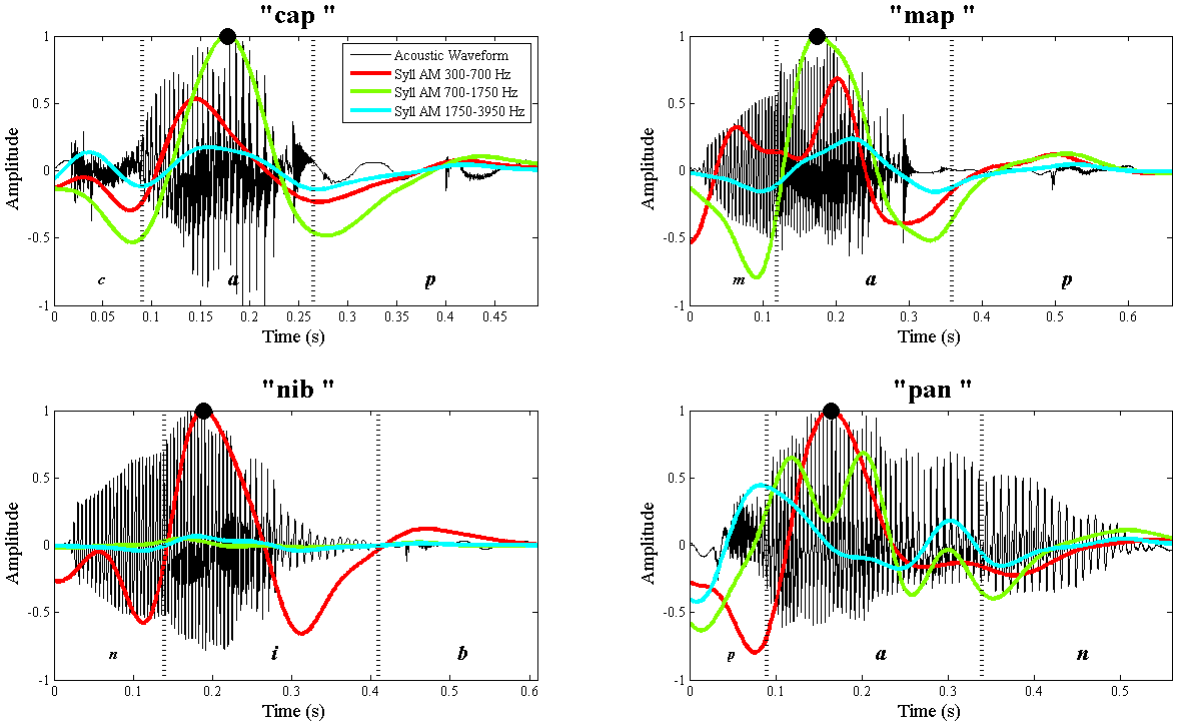
Acoustic waveforms and corresponding Syllable AMs for 4 different CVC syllables. The syllables are: "cap" (top left), "map" (top right), "nib" (bottom left) and "pan" (bottom right). For each word, its acoustic waveform is shown in black, and the Syllable AMs from spectral bands 2,3 & 4 are overlaid as coloured lines. The vertical dotted lines in each plot indicate the manually-located boundaries between the onset, vowel nucleus and coda. Note that for each word, the highest Syllable AM peak (point of greatest energy) falls within the vowel nucleus, as indicated with black dots. However, the spectral band with the highest Syllable AM peak varies across words. For example, for "cap" and "map", it is spectral band 3 (700–1750 Hz, green) which carries the strongest peak. For "nib" and "pan", it is spectral band 2 (300–700 Hz, red) which carries the strongest peak.

The purpose of the S-AMPH model is to use the core modulation architecture of CDS (identified by PCA) to perform acoustic-phonological mapping—a computational process whereby AM patterns and statistics are used to extract the primitive phonological units in the English language (i.e. syllables, onset-rime units and prosodic stress patterns). The principles and algorithms used by the model to find syllables are as follows:

For a spoken syllable, the point of highest energy typically corresponds to the vowel nucleus [21,51]. During vowel phonation, the vocal tract is at its most open and permits the greatest airflow, while during the articulation of obstruent consonants in the onset and coda it is at its most constricted. Thus, the acoustic waveform of a typical CVC (consonant-vowel-consonant) syllable is shaped like a hill with its apex located within the vowel nucleus (describing its sonority profile), as shown in Fig 4. Meanwhile, less energetically-prominent (*unstressed*) syllables are less reliably encoded in children's phonological representations of words (e.g. very young children will say "NANA" (Strong-weak, or “S-w”) instead of "ba-na-na" (weak-Strong-weak, or "w-S-w"); [52–53]).

Fig 4 shows that local *peaks* in syllable-rate modulations (i.e. peaks in the Syllable AM) map closely to syllable vowel nuclei. As expected, each monosyllable generally elicits a single large Syllable AM peak (although there are exceptions, discussed later). Moreover, the single highest Syllable AM peak out of all the spectral bands (indicated with black dots) consistently falls within the vowel nucleus, approximately at the point of highest amplitude in the acoustic waveform. However, there are important caveats to note. First, across the 4 different words, different spectral bands yield the highest Syllable AM peak (e.g. here, either band 2 or band 3). Therefore, the output from any single spectral band will not provide robust syllable location across all possible words. The model needs a principled way to weight and select the most appropriate spectral band for use in syllable vowel nucleus detection. Second, each Syllable AM may occasionally contain *secondary* peaks that correspond to other voiced segments within the syllable, such as the /m/ in "map" (top right plot, red line has 2 peaks). The presence of these secondary peaks undermines the assumption of 1:1 mapping between Syllable AM cycles and syllable units (vowel nuclei), and could lead to spurious findings in the S-AMPH model. Therefore, a principled way to screen out these irrelevant (i.e. not vowel-related) secondary peaks in the Syllable AM is required. The following computational procedure has 3 steps, detection, ranking and selection, and includes safeguards to address the problem of secondary peaks.

#### (a) Candidate Peak Detection

**Fig 5:**
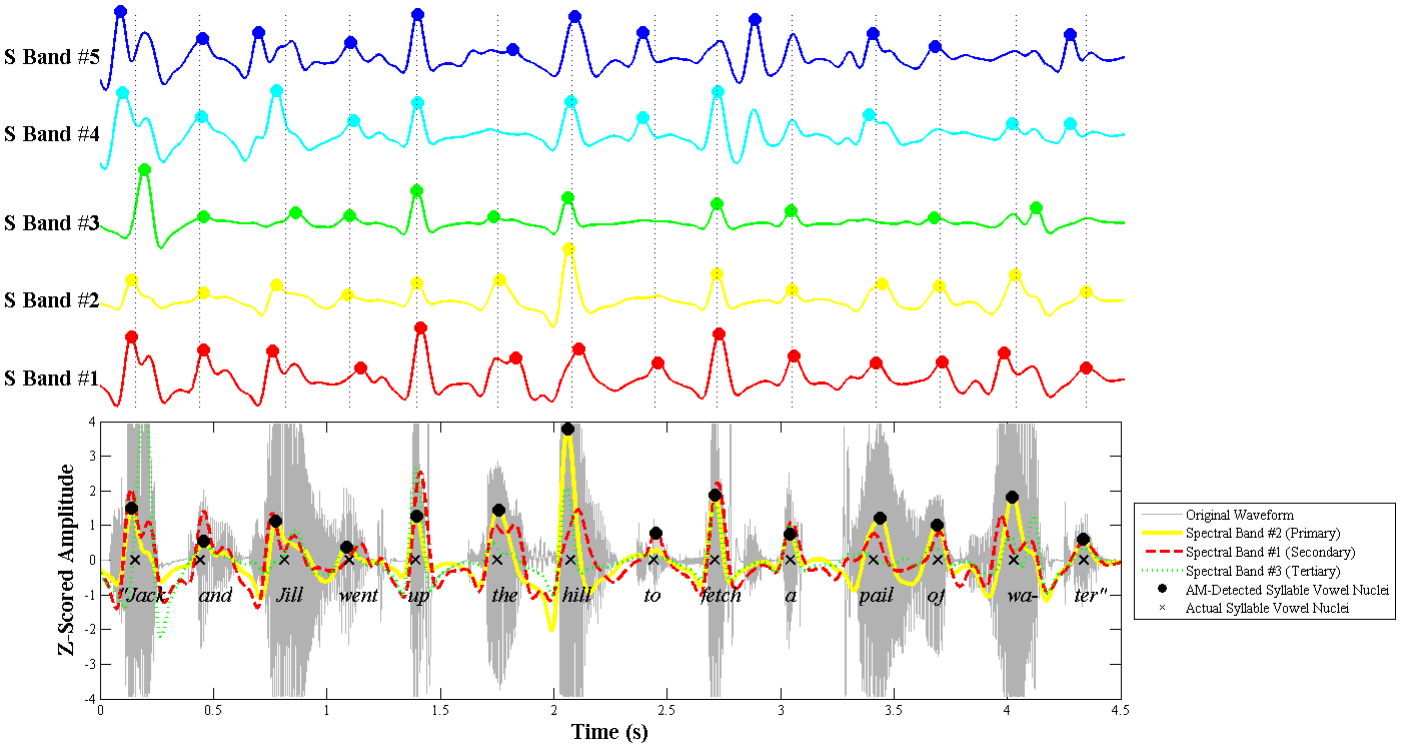
Example of Syllable AM peak detection and evaluation. The example shows the trochaic nursery rhyme sentence "Jack and Jill". (Top). The Syllable AMs from spectral bands 1–5 are each plotted in separate rows in different colours. The original pool of candidate peaks that were detected in these 5 AMs are shown as coloured dots. The vertical dotted lines indicate the actual (manually-annotated) location of syllable vowel nuclei in the sentence.(Bottom) Results of the peak evaluation procedure. The Syllable AMs from 3 power-ranked primary (Band #2), secondary (Band #1) and tertiary (Band #3) spectral bands are overlaid on the original acoustic waveform. The final selected Syllable AM peaks (= model-detected syllable vowel nuclei) are shown as black dots. The actual syllable vowel nuclei in the utterance are shown as black crosses. Notice that the Syllable AM peaks in the primary spectral band (#2, yellow) correctly correspond to almost all the actual syllable vowel nuclei, with the exception of the syllable "to", whose vowel nucleus is captured within the secondary spectral band (#1, red).

First, all the Syllable AM peaks across the 5 spectral bands are identified using an automated MATLAB peak detection algorithm ([50], 'findpeaks.m', see S1 Code for formal description), resulting in a large pool of candidate peaks. Examples are shown in the top half of Fig 5. This peak detection algorithm allows the listener to take into account variations in *modulation depth* and *speaking rate* via two parameters. Variations in modulation depth are accounted for by z-scoring each Syllable AM, and then setting the minimum peak height (MPH) for peak detection at +0.5 standard deviations. Variations in speaking rate are accounted for by setting the minimum peak-to-peak distance (MPD) at 60% of the syllable period (duration) for each sample. The syllable period is estimated on a sample-to-sample basis by applying a discrete Fourier transform, computed with a MATLAB fast Fourier transform algorithm ([50], 'fft.m', see S1 Code for formal description) to each Syllable AM and then determining the modulation frequency component with the highest power. This was based on the assumption that the main peak in the modulation spectrum of speech, which typically occurs ~3–5 Hz, corresponds to the energy of uttered ADS syllables [21]. Therefore the peak modulation rate of the Syllable AM should indicate the main syllable rate of the utterance. For example, if the largest frequency component identified in the Fourier analysis is 3 Hz, the MPD for the sample would be 0.6 x (1000/3) = 200 ms.

#### (b) Candidate Peak Ranking

In the second step, the large pool of candidate AM peaks is reduced, and peaks are ranked in order of vowel likelihood according to the root-mean-square (RMS) power of their spectral band of origin. Since English vowels are voiced, it follows that the spectral band with the highest power should also be transmitting the most vowel energy. Thus, Syllable AM peaks arising from this highest-powered spectral band should be the most likely to correspond to syllable vowel nuclei (i.e. have the highest vowel likelihood). However, the main frequency of vowel energy could be different for different speakers, and for different samples. Moreover, even within the same sample for the same speaker, not all of his or her vowels will fall in the same spectral band due to variations in token type (e.g. /a/ vs /i/), or prosodic stress. Thus, even lower-powered spectral bands could contain vowel energy. To address these concerns, we determine the highest-powered spectral band individually for each speaker and sample. Moreover, we retain the Syllable AM peaks from the top 3 highest-powered spectral bands (not just the top 1 band) as potential correlates of syllable vowel nuclei, discarding only the peaks from the two lowest-powered spectral bands. These top 3 spectral bands are designated accordingly as the primary band (highest RMS power), secondary band (2nd highest RMS power) and tertiary band (3rd highest RMS power). This is shown in the bottom half of Fig 5. Thus, the second step in the algorithm yields a reduced pool of candidate AM peaks, each ranked as being either primary, secondary or tertiary in vowel likelihood (according to their spectral band of origin).

#### (c) Candidate Peak Evaluation

In the final step, the power-ranked AM peaks are systematically evaluated for overlaps in timing to eliminate duplication and irrelevant peaks. Duplication occurs when there are multiple AM peaks across primary, secondary and tertiary bands that correspond to the same syllable vowel nucleus. Irrelevant peaks are AM peaks that do not correspond to a real syllable vowel nucleus. According to the S-AMPH model, the vast majority of uttered syllable vowel nuclei should produce Syllable AM peaks in the primary spectral band. However, a smaller number of syllable vowel nuclei will also generate Syllable AM peaks in the secondary or tertiary spectral bands. To distinguish whether secondary and tertiary peaks correspond to genuine outlying syllable vowel nuclei, or whether they are simply duplicates, a temporal proximity criterion is applied. Syllables are uttered sequentially in time. Therefore, secondary and tertiary AM peaks that correspond to outlying syllable vowel nuclei should lie at some temporal distance from existing primary Syllable AM peaks, rather than overlapping in time. Accordingly, in the model each secondary-ranked AM peak is temporally compared to the existing set of primary AM peaks. Any secondary peak that lies within ±0.5 syllable-lengths of a primary peak (based on the estimated syllable rate for that sample, using the estimation procedure described in the previous section on candidate peak detection) is treated as a duplicate and discarded. The surviving secondary peaks are retained and added to the set of primary peaks. Finally, the procedure is repeated by comparing each tertiary peak against the existing bank of primary *and* any surviving secondary AM peaks. This evaluation procedure yields the final set of Syllable AM peaks that are deemed to correspond to syllable vowel nuclei in the utterance. In Fig 5, this final set of Syllable AM peaks are shown as black dots in the bottom half of the figure. In this example, these model-detected Syllable AM peaks correspond perfectly to the actual (manually annotated) syllable vowel nuclei in the utterance, shown as black crosses in the figure.

### The S-AMPH Computational Model for Acoustic-Phonological Mapping 2: Finding Onset-Rime Divisions within Syllables

Similar to the perceptual onset models by Pompino-Marschall [54] and Harsin [55], here we use a modulation rate-of-change function within different spectral bands to model the onset-rime division within syllables. We assumed that points on the Syllable AM where there was a rapid rate of amplitude increase (i.e. a rapid rise time) corresponded to onset-rime divisions, because there is typically a sharp increase in energy associated with the transition from a consonant (onset) to a vowel (rime beginning). These sharp increases in amplitude were detected by first computing the derivative (rate of change) of the z-scored Syllable AM, and then identifying peaks in this derivative function using MATLAB's automated peak detection algorithm (using the same settings for minimum peak height and mean peak distance as for the Syllable detection procedure). This procedure was performed for the Syllable AM in all 5 spectral bands, and the resulting pool of identified onset-rime division peaks was passed through identical serial ranking and evaluation procedures as described in Steps 2 & 3 of the previous Syllable-finding procedure (which assess vowel likelihood and eliminate duplications respectively). Note that we did not utilise the Phoneme AM to extract the onset-rime division, as empirical data suggest that phoneme awareness in children is a product of learning to read [1,2,4].

### The S-AMPH Computational Model for Acoustic-Phonological Mapping 3: Determining the Prosodic Strength of Syllables

Finally, we focus on how the Stress-Syllable AM phase relationship may be used to compute the prosodic prominence of syllables in an utterance. We propose a Prosodic Strength Measure (PSM) which transforms the Stress-Syllable AM phase relationship into an empirical measure of whether a given syllable is prosodically-strong ('S') or weak ('w'). By computing the PSM for each syllable, the overall rhythm pattern of an utterance may be revealed.

#### (a) The Prosodic Strength Measure (PSM)

**Fig 6:**
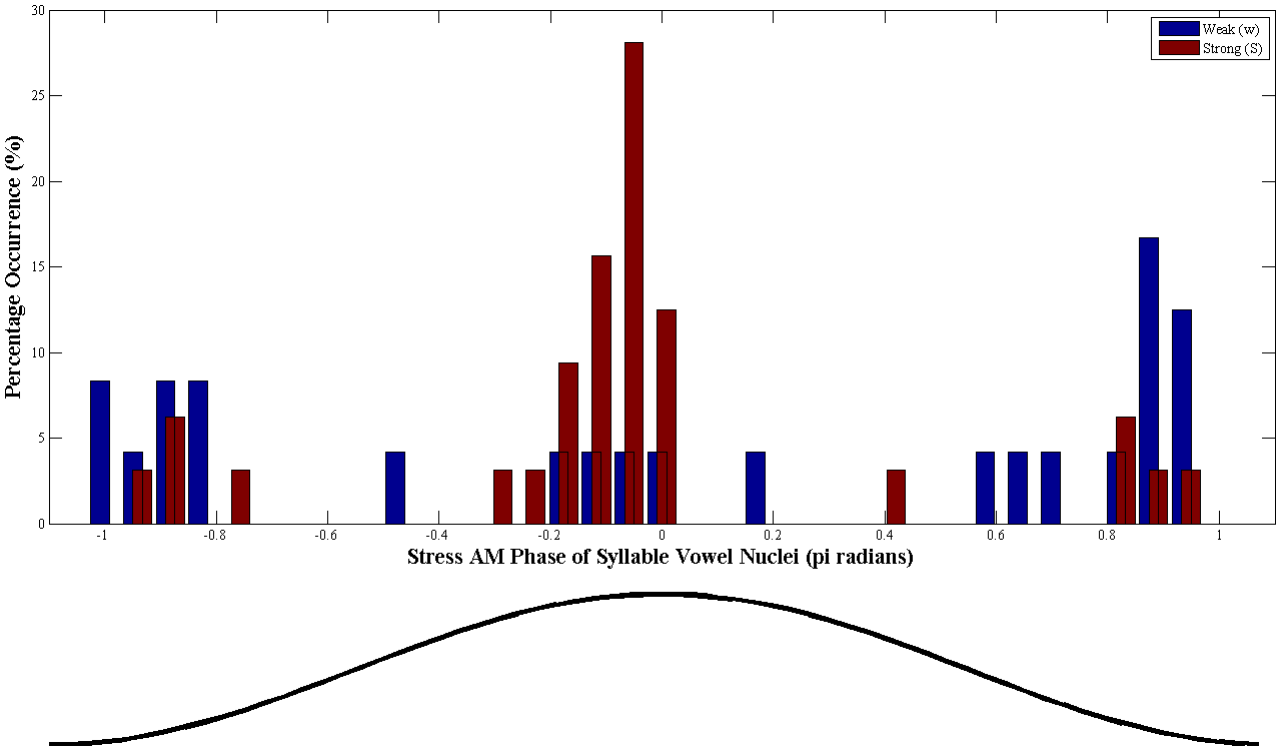
Distribution of instantaneous Stress AM phase for 'Strong' (red) and 'weak' (blue) syllable vowel nuclei. The example shown is for the trochaic-patterned nursery rhyme sentence 'Jack and Jill', and results are computed over the pooled syllable vowel nuclei from 2 speakers. The x-axis indicates the instantaneous Stress AM phase of the syllable vowel nuclei, binned into 17 equal phase bins between -π and _+_π radians. The curve below shows the equivalent oscillatory shape of the AM phase values on the x-axis.

In the AM hierarchy, daughter tiers are modulated in amplitude by parent tiers. Consequently, the amplitude (or loudness) of a given Syllable AM cycle will depend on the modulation that is imposed by its parent Stress AM cycle. If the Stress AM is at a peak (i.e. 0π radians phase), it follows that syllables occurring during this peak phase will receive stronger modulation and therefore be more prominent. Conversely when the Stress AM is at its trough (-π/+π radians phase), syllables occurring at this trough phase will receive less modulation and be less prominent. To test this assumption, the Stress AM phase distribution for known 'Strong' and 'weak' syllables was computed using one of the metronome-timed prosodic templates from Corpus 2, as produced by 2 native English speakers (1M, 1F). The speakers repeated the nursery rhyme *"Jack and Jill went up the hill to fetch a pail of water*, *Jack fell down and broke his crown*, *and Jill came tumbling after*" in time to a 3-Hz metronome beat using trochaic patterning. This template was chosen as the trochaic pattern occurs most frequently in English nursery rhymes [26], and utterances following this pattern should contain an equal number of 'Strong' and 'weak' syllables (i.e. a numerically balanced sample). The syllable vowel nuclei in both utterances were then manually located using Praat software [46], and each vowel's corresponding Stress AM phase was computed. The resulting Stress phase distribution obtained for Strong and weak syllables is shown in Fig 6. As expected, the vast majority of 'Strong' syllables occurred near the oscillatory peak of the Stress AM (~0π radian), with the highest occurrence at around -0.1π radians. By contrast, 'weak' syllables tended to occur more often near the oscillatory trough of the Stress AM (~-π/+π radian).

To accurately capture this probabilistic distribution of 'Strong' versus 'weak' syllables with regard to Stress AM phase, the PSM function should have a shape where it assigns maximum prosodic prominence (i.e. 1) at around -0.1π radian, and minimum prominence (i.e. 0) at ~-π/+π radian. As the exponential probability density function (PDF) is unimodal, symmetric, and has a convex shape, it was used for this purpose. To compute the PSM, the absolute instantaneous Stress AM phase value of the Syllable AM peak was taken, shifted by 0.12π radians (to reflect the shifted peak in the Stress AM phase distribution of strong syllables), and the corresponding probability under an exponential PDF with a μ of 1 was taken. Thus, PSM values range from a minimum of 0 (= lowest likelihood of a strong syllable) to a maximum of 1 (= highest likelihood of a strong syllable). To reflect the relatively narrow spread of Stress AM phase values occupied by 'Strong' syllables in the previous analysis, we estimated that syllables achieving PSM values of at least 0.4 would be considered 'Strong', whilst those achieving PSM values under 0.4 would be considered 'weak'.

### Evaluation Criteria for S-AMPH Acoustic-Phonological Mapping Accuracy

The accuracy of acoustic-phonological mapping at each phonological level (syllable, onset-rime and prosodic stress) was assessed using signal detection theory, which computes both the detection (hit) rate, as well as the false alarm rate of the model.

#### (a) Syllable Finding Performance

A 'hit' was recorded when a Syllable AM peak that was selected by the model lay within +/- half the mean syllable length (for that sample) of the real manually-annotated syllable vowel nucleus. A 'miss' was recorded when a manually-annotated syllable vowel nucleus failed to correspond to any Syllable AM peak that was selected by the model. A 'false alarm' was recorded when a Syllable AM peak that was identified by the model failed to correspond to any manually-annotated syllable vowel nuclei. A ‘correct rejection’ was recorded when a Syllable AM peak that did *not* correspond to a syllable vowel nucleus was correctly eliminated or ignored by the model. To compute the number of correct rejections, an estimate of the *total* number of 'spurious peaks' that did not correspond to a real syllable vowel nucleus was required. This spurious peak estimate was generated by detecting all possible peaks in the sample by setting *no* criteria on the mean peak height or distance (i.e. *any* point that was higher than its surrounding neighbours was included), dividing this total peak number by 5 (to normalise for the 5 Syllable AMs across the 5 Spectral bands), and subtracting the number of manually-annotated syllable vowel nuclei from this normalised peak number. Finally, the number of correct rejections was computed by subtracting the number of false alarms from the spurious peak estimate.

#### (b) Onset-Rime Division Performance

A 'hit' was recorded when an onset-rime division that was identified by the model lay within +/- 30 ms of the real manually-annotated onset-rime division. A 'miss' was recorded when a manually-annotated onset-rime division failed to correspond to any onset-rime division that was identified by the model. A 'false alarm' was recorded when an onset-rime division that was identified by the model failed to correspond to any manually-annotated onset-rime division. A ‘correct rejection’ was recorded when a Syllable AM derivative peak that did *not* correspond to an actual onset-rime division (i.e. a spurious peak) was correctly eliminated or ignored by the model. As before, to compute the number of correct rejections, an estimate of the *total* number of spurious peaks that did not correspond to a real onset-rime divisions was required. This spurious peak estimate was generated by detecting all possible peaks in the Syllable AM derivative function by setting *no* criteria on the mean peak height or distance (i.e. *any* point that was higher than its surrounding neighbours was included), dividing this total peak number by 5 (to normalise for the 5 Syllable AMs across the 5 Spectral bands), and subtracting the number of real onset-rime divisions from this normalised peak number. Finally, the number of correct rejections was computed by subtracting the number of false alarms from the spurious peak estimate.

#### (c) Stress-Detection Performance

In the stress-detection exercise, syllables were assigned either a 'Strong' or 'weak' status, using the Prosodic Strength Measure (PSM) described previously. For the timed CDS sample, the threshold PSM value used for assigning a 'Strong' status was 0.4, which produced excellent results. However, for the untimed CDS samples, a range of PSM threshold values was tested, to see which value would yield the most optimal results. The results of PSM threshold assessment are shown as an ROC curve in Figure A of S5 Appendix. It should be noted that the value of the PSM threshold that is used for 'Strong' stress assignment affects the trade-off between the number of hits and the number of false alarms achieved by the S-AMPH model. If the PSM threshold is high, only very strongly-stressed syllables will be assigned a 'Strong' status, leading to fewer hits but also fewer false alarms. Conversely, if the PSM threshold is very low, stressed syllables will be detected very well (i.e. a high hit rate), but some unstressed syllables may be incorrectly assigned a 'Strong' status as well (i.e. a high false alarm rate).

'Hits' were defined as prosodically-stressed syllables that were correctly assigned a 'Strong' status. 'Misses' were defined as prosodically-stressed syllables that were incorrectly assigned a 'weak' status. 'False alarms' were defined as unstressed syllables that were incorrectly assigned a 'Strong' status. 'Correct rejections' were defined as unstressed syllables that were correctly assigned a 'weak' status. For this exercise, only correctly identified syllable peaks were included in the analysis (i.e. 'hits' from the previous syllable-finding exercise). This was done so that the effectiveness of prosodic stress detection could be evaluated independently of the model's success in identifying syllables.

## Results

### Spectral and Modulation Rate (Temporal) PCA Analyses for the Dominant Spectro-Temporal Modulation Architecture of Child-Directed Speech

The aim of these analyses was to derive a low-dimensional representation of the core spectro-temporal modulation architecture of child-directed speech. Accordingly, Principle Component Analysis (PCA) was adopted as our method of choice for dimensionality reduction, and the PCA procedure was applied separately for (1) spectral and (2) modulation rate (temporal) dimensionality reduction respectively. In each dimension (spectral and rate), the procedure was equivalent to finding bands of acoustic frequencies or modulation rates in which the energy changed in a similar way over time (i.e. co-modulation). For both PCA exercises, a high-dimensional speech representation (i.e. a high number of audio frequency or modulation rate channels) was used as the input substrate, and the rectified loading patterns of the derived principle components (PCs) were used to identify patterns of covariation between the input channels (see Methods for explanation). The PCA method has long been used for dimensionality reduction in speech studies (e.g. [56–57]). However, unlike a typical PCA analysis, here the key outcome of interest was not the component scores, but the component *loadings*. Component loadings indicate the underlying patterns of correlation between channels. By analysing the patterns of component loading across the channels in a high-dimensional representation, we reasoned that one should be able to identify groups of adjacent channels that belong to the same core band of spectral or temporal modulation.

#### Spectral PCA

**Fig 7:**
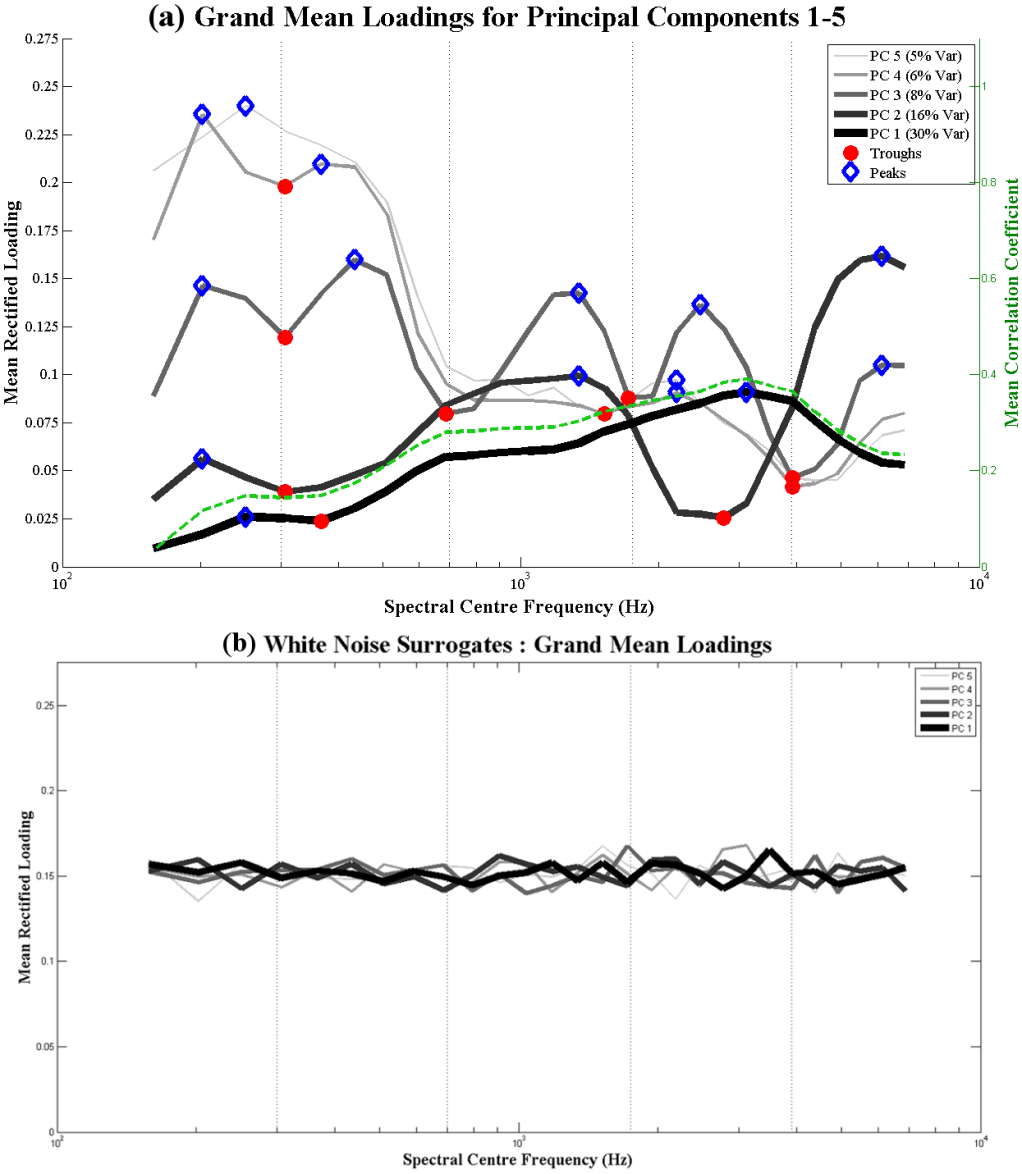
Spectral PCA loading patterns for CDS corpora (7a, top) and white noise surrogates (7b, bottom). **(7a)** (Left y-axis) Grand average rectified loadings for the top 5 principal components arising from the Spectral PCA of the CDS corpora, averaged across all speech samples from 6 speakers. The blue diamonds mark peaks in loading and the red dots mark troughs in loading. On the basis of these peak and trough patters (see text), 5 spectral bands are inferred, whose boundaries are shown as vertical dotted lines. (Right y-axis) Grand average correlation coefficient between each spectral channel and all the other spectral channels, plotted as a dotted green line. (7b) Grand average rectified loadings for the top 5 principal components arising from the Spectral PCA of white noise surrogates of the CDS corpora, averaged across all speech samples from 6 speakers. Note that there is no systematic pattern of peaks.

Fig 7 shows the grand average rectified loading patterns for the five principal components arising from the spectral PCA conducted with the Nursery Rhyme Corpus 1 (see Methods), averaged across all 6 speakers. For more detail on other aspects of the spectral analysis, please see S3 Appendix, which shows each *individual* speakers' rectified loading patterns for the spectral PCA, and S2 Appendix, which details the root-mean-square (RMS) power and cross-correlation across spectral channels. In Fig 7a, the peaks and troughs that were automatically identified for each PC are overlaid as blue diamonds (peaks) and red circles (troughs). In the figure, lower numbered PCs that account for more variance are shown in darker, thicker lines. As a control, we carried out an identical PCA analysis on a generated corpus of white noise surrogates that were matched in length to each stimulus in the CDS corpus. The grand mean rectified loading patterns obtained for this control analysis using white noise surrogates are shown in Fig 7b.

*PC1*: The first principal component (PC1) accounted for, on average, 30.4% of the total variance in the data. Fig 7a shows that PC1 (the thickest line) had a relatively low but even pattern of loading, which was highest in the mid-frequency range (~1000–4000 Hz). As may be clearly observed, the first PC reflects the mean correlation between spectral channels. Its rectified loading pattern was virtually identical to that of the mean correlation coefficient between each spectral channel and all the other spectral channels (plotted as a dotted green line). This similarity between the rectified loading pattern of PC1 and the average correlation between channels suggests that the PCA process is successful in drawing out some meaningful statistical relationships that are present within the modulation data. Two peaks (~250 Hz, ~3000 Hz) and 1 trough (~375 Hz) were identified from the loading pattern of PC1. However, these were not the sharp reversals in loading pattern that we sought, which would indicate transitions between core bands of spectral energy. Consequently, we considered PC2.

*PC2*: The second principal component (PC2) accounted for, on average, 16.1% of the total variance in the data. Unlike PC1, PC2 showed a more distinct loading pattern of peaks and troughs, with 3 prominent peaks at acoustic frequencies of ~200 Hz, ~1000 Hz and ~5000 Hz respectively. The frequency of these 3 peaks suggests that they might possibly correspond to speech features such as fundamental frequency, voicing and frication respectively. Thus, PC2 provided evidence for the potential existence of at least 3 core spectral bands in the CDS data.

*PC3*: The third principal component (PC3) accounted for, on average, 7.6% of the total variance in the data. The loading pattern of PC3 provided the most convincing evidence for the existence of spectral banding. There were 5 distinct peaks identified at acoustic frequencies of ~200 Hz, ~500 Hz, ~1000 Hz, ~2500 Hz, and ~5000 Hz respectively. Peaks 1, 3 and 5 occurred in identical locations to the 3 peaks previously observed in PC2, corroborating the evidence for 3 spectral bands at these 3 spectral locations. In addition to peaks, PC3 also showed 4 distinct troughs at acoustic frequencies of ~300 Hz, ~700 Hz, ~1750 Hz, and ~4000 Hz respectively, which indicated the potential boundaries between up to 5 spectral bands. Therefore, PC2 and PC3 collectively provided evidence for at least 3 spectral bands (~200 Hz, ~1000 Hz, ~5000 Hz), with PC3 additionally suggesting the existence of a further 2 spectral bands at ~500 Hz and ~2500 Hz respectively.

*PC4 & PC5*: Principal components 4 & 5 (PC4 & PC5) accounted for, on average, 6.0% and 5.3% of the total variance in the data respectively. The rectified loading patterns for these two PCs were highly similar, showing the strongest loading at low acoustic frequencies. Like PCs 1, 2 and 3, PCs 4 & 5 also showed peaks in loading at ~200–250 Hz, corroborating the evidence for a spectral band in this region (potentially corresponding to the fundamental frequency in speech). Given that PC3 had suggested the potential existence of 2 spectral bands at ~500 Hz and ~2500 Hz, we were particularly interested in whether PCs 4 & 5 would corroborate this. As may be seen from Fig 7a, PC4 did indeed show a peak at ~400 Hz, while *both* PCs 4 & 5 showed small peaks at ~2000 Hz. It is possible that these two additional spectral bands at ~500 Hz and ~2000–2500 Hz could correspond to formant energy (e.g. F1 & F3).

Thus, following our criteria (see Methods), we had indeed obtained sufficient evidence for the presence of 5 core spectral bands in the spectral modulation data, with at least 2 out of 5 PCs showing peaks in each of these 5 spectral regions. We then determined the upper and lower boundaries for each of these 5 spectral bands using the troughs in PC3 (which had shown 5 distinct peaks and 4 distinct troughs), whose locations coincided with the troughs in several other PCs. Table 4 provides a summary of the 5 spectral bands that were identified, and their respective frequency ranges. It is interesting to note that each spectral band comprises a roughly similar number of the original ERB_N -_spaced (i.e. cochlear-spaced, [47]) spectral channels, suggesting that the 5 speech spectral bands are proportionately-scaled with respect to the logarithmic frequency sensitivity of human hearing. That is, the distribution of spectral modulation energy within the input speech signal is well-matched to the receiver characteristics of the listener. There is also some evidence of a broadening of spectral bandwidths at mid-frequencies, since spectral bands 3 & 4 each span a slightly larger number (7) of ERB_N_ channels compared to the other bands. These mid-frequency bands might be expected to transmit the modulation patterns associated with energetically-rich vowel sounds, which are hyperarticulated in child-directed speech (hence in our speech corpus), and thus occupy an expanded spectral range.

**Table 4:** Summary of the 5 spectral bands indentified from PC loading patterns.

Spectral Band	PC Peaks	No of ERB_N_ Channels	Frequency Range (Hz)
Band 1	PC1 - PC5	4	100–300
Band 2	PC3, PC4	5	300–700
Band 3	PC2, PC3	7	700–1750
Band 4	PC3 - PC5	7	1750–3900
Band 5	PC2, PC3	5	3900–7250

It is important to note that the results we report here are inferred from the *grand average* PC loading patterns, obtained by taking the mean of loadings across 6 speakers. However, it is reasonable to expect that there will be some individual variation in peak and trough location across different speakers. This individual variation is explored and described further in S3 Appendix. In summary, we find that there is highest consistency across speakers at low spectral frequencies. In particular, the trough in loading that indicates a division between spectral bands 1 & 2 at 300 Hz is consistently present across speakers. However, we find that there is more individual variation at mid-to-high spectral frequencies.

#### Modulation Rate PCA

**Fig 8:**
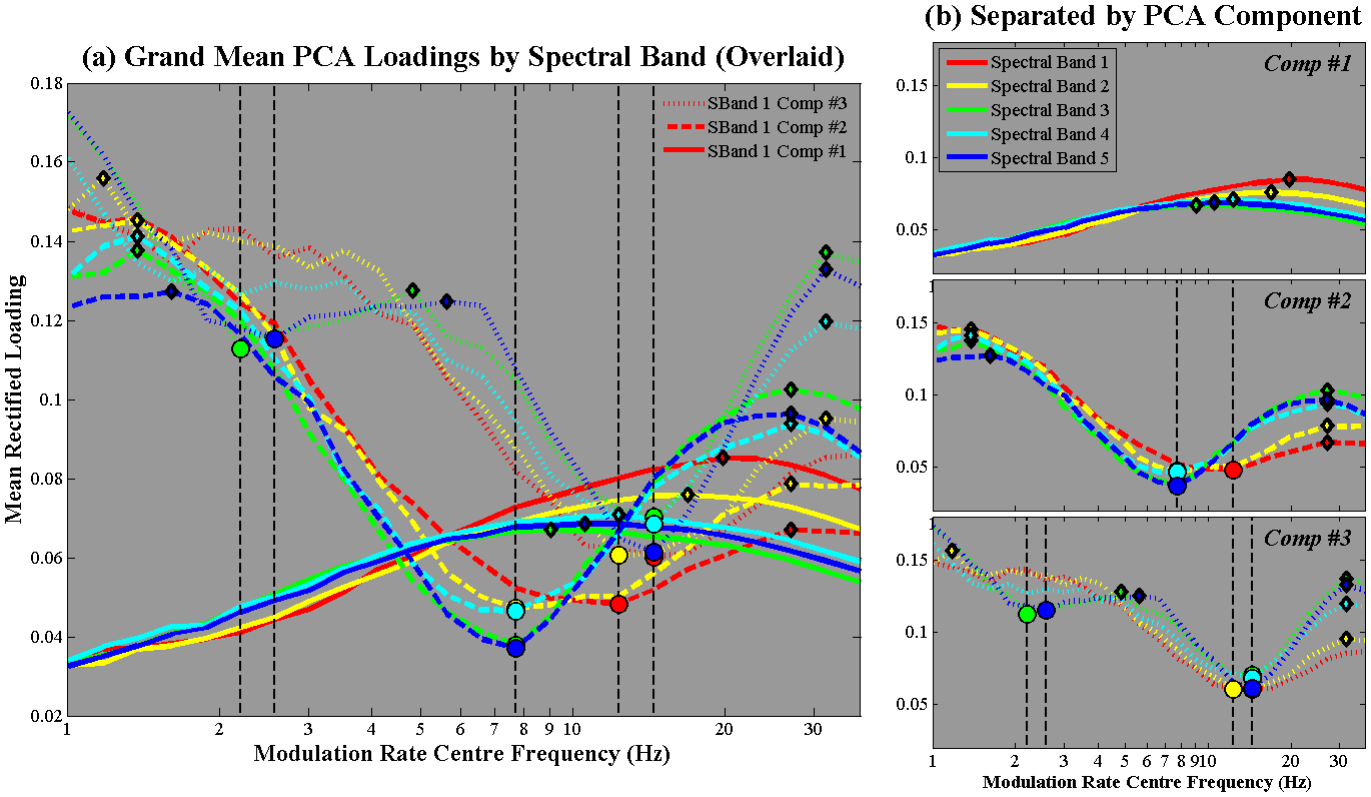
Modulation rate PCA resulting loading patterns. Mean rectified loading patterns for the first 3 principal components obtained for spectral bands 1–5 in the modulation rate PCA, averaged over all speech samples. In (a, left), the PC loading patterns for each spectral band are overlaid. In (b, right), the same loading data is separated by PCs into subplots for clarity. For both (a) and (b), the 5 different colours indicate the 5 different spectral bands. The linestyle (solid, dashed or dotted) indicates the PC number. Coloured circles show local troughs in loading that indicate potential boundaries between modulation rate bands (also marked on the x-axis with vertical dotted lines). The coloured diamonds show local peaks in loading that indicate clusters of modulation rate channels that show strong covariance in temporal patterning. The colour of each circle or diamond reflects its spectral band, following the main colour scheme.

Fig 8 shows the grand average rectified loading patterns for principal components 1–3 arising from the modulation rate PCA conducted for each of the 5 spectral bands (determined in the previous Spectral PCA, see Table 4). In the figure, the 5 spectral bands are indicated in different colours, while PCs 1,2 and 3 are indicated in bold, dashed and dotted lines respectively. The peaks and troughs that were automatically identified for each PC are overlaid as diamonds (peaks) and circles (troughs), coloured according to their spectral band of origin. The left portion of the figure (a) shows the 3 PCs overlaid, while the right portion of the figure (b) shows the same data, but with the PCs separated into subplots. Table 5 provides a summary of the location of the peaks and troughs detected for each PC, with further detail and explanation provided in the following text. For each *individual* speakers' rectified loading patterns for the modulation rate PCA, please see S3 Appendix.

**Table 5:** Peak and trough locations for PC1-PC3.

Component (variance)	Peak locations	Trough locations*(annotated with vertical dotted lines in Fig 8)*
PC1 (47.2%) *Note*: *Reflects global correlation*	10–20 Hz (all bands)	N.A.
PC2 (16.7%)	~1.5 Hz (all bands), ~30 Hz (all bands)	~12 Hz (band 1), ~8 Hz (bands 2–5)
PC3 (9.5%)	~1 Hz (band 2), ~5 Hz (bands 3 & 5) ~30 Hz (all bands)	~2.5 Hz (bands 3 & 5), ~12 Hz (band 2), ~14 Hz (bands 3 & 5)

*PC1*: Similar to what was observed in the spectral PCA, PC1 in this modulation rate analysis reflected the global correlation between modulation rate channels (data not shown), which was strongest at around the rates of 10–20 Hz across the 5 spectral bands. It is interesting to note that this peak in modulation *correlation* strength does not occur in the same modulation range as the peak in modulation *power*, which typically occurs at a much lower rate (~3–5 Hz for ADS [21]; ~2 Hz for CDS [23]). Thus, the two modulation statistics (power and correlation) could relate to qualitatively different aspects of the speech signal. As no troughs were detected in PC1 (indicating potential boundaries between modulation rate bands), our analysis was centred primarily on PC2 and PC3.

*PC2*: The rectified loading pattern of PC2 clearly consisted of 2 strong peaks (~1.5 Hz and ~30 Hz) which were separated by a deep trough. However, the modulation rate of this trough varied across different spectral bands. For spectral band 1, the trough occurred ~12 Hz, whereas for higher frequency spectral bands 2–5, the trough occurred earlier at ~8 Hz. Thus, this loading pattern clearly suggested the presence of at least 2 modulation rate bands, but the boundary between the bands was unclear. Consequently, we sought clarification by examining PC3.

*PC3*: Here, we observed some similarities to the loading patterns observed in PC2. For example, almost all the spectral bands showed a peak in loading at ~30 Hz, and at least one spectral band (2) similarly showed a slow-rate peak at ~1 Hz. However, unlike PC2, the loading patterns for PC3 spectral bands 3 & 5 showed an *additional* mid-rate peak at ~5 Hz. The flanking troughs for this additional peak in spectral bands 3 & 5 occurred ~2.5 Hz and ~14 Hz respectively, although spectral band 2 also showed a trough at ~12 Hz (similar to spectral band 1 for PC2).

Thus, following our criteria for identification of modulation rate bands (see Methods), the *peaks* from the loading patterns of PC2 and PC3 collectively pointed to the presence of 3 separate modulation rate bands with boundaries at ~2.5 Hz and ~12 Hz respectively. However, the pattern of *troughs* suggested that there could be an *additional* boundary within the second (mid-rate) band, dividing this band into a lower sub-band (2.5–8 Hz) and an upper sub-band (8–12 Hz) respectively. These boundaries are marked with vertical dotted lines in Fig 8.

The identification of 3 major modulation rate bands or modulation timescales in Nursery Rhyme Corpus 1 fits well with theoretical proposals regarding the typical timescales of 3 major phonological units in speech: *stress feet* (~2 Hz; [27]), *syllables* (~5 Hz; [21,36,51]) and *onset-rimes*/*phonemes* (~20 Hz, [58–59]). This correspondence is shown in Table 2 and discussed further in the Discussion. Accordingly, these 3 acoustic modulation rate bands are henceforward assumed to support the extraction of phonological units (prosodic stress, syllables and onset-rime/phoneme units respectively). We hereafter refer to these 3 AM bands as the 'Stress AM' (0.9–2.5 Hz), the Syllable AM (2.5–12 Hz) and the Onset-Rime/Phoneme AM (12–40 Hz). In the following study, we test the validity of assuming an acoustic-phonological correspondence at these key temporal rates by applying the S-AMPH to 2 other CDS corpora (Nursery Rhyme Corpus 2 and Nursery Rhyme Corpus 3). Note that there is a close similarity between the core modulation timescales of CDS and the core bands of oscillatory activity observed in the human cortex, as shown in the far right column of Table 6.

**Table 6:** Summary of the 3 modulation rate bands indentified from PCA.

Linguistic Unit	Modulation Rate Band (Geometric CF) in Hz	Neural Oscillatory Bands in Hz
**Prosodic Stress** (Mod band 1)	0.9–2.5 (1.5)	**Delta**: 1–3
**Syllable** (Mod band 2)	*Lower*: 2.5–8 (4.5)	**Theta**: 4–7
	*Upper*: 8–12 (9.8)	**Alpha**: 8–12
**Onset-Rime/Phoneme** (Mod band 3)	12–40.0 (21.9)	**Beta**: 13–25 **Low Gamma**: 25–35

Indeed, multi-time resolution models of speech processing (e.g. [34,36]) suggest that this close matching between the timescales of speech and brain activity reflects a biologically-expedient mechanism for the concurrent neural encoding or 'temporal sampling' of important speech information at different timescales. Moreover, the upper and lower sub-bands within the 'Syllable' band (modulation band 2) correspond well to the typical timescales of stressed vs unstressed syllables (e.g. [21]: <6 Hz for stressed syllables, 6–20 Hz for unstressed syllables) as well as to the timescales of content vs function words in infant-directed speech (e.g. [60]: <8 Hz for content words, >8 Hz for function words), where content words are more likely to receive stress than function words. Thus, the emergent modulation statistics of CDS appear to fit some core requirements for language learning in terms of extracting phonological and grammatical structure.

### Resulting Spectral Amplitude Modulation Phase Hierarchy Model (S-AMPH)

**Fig 9:**
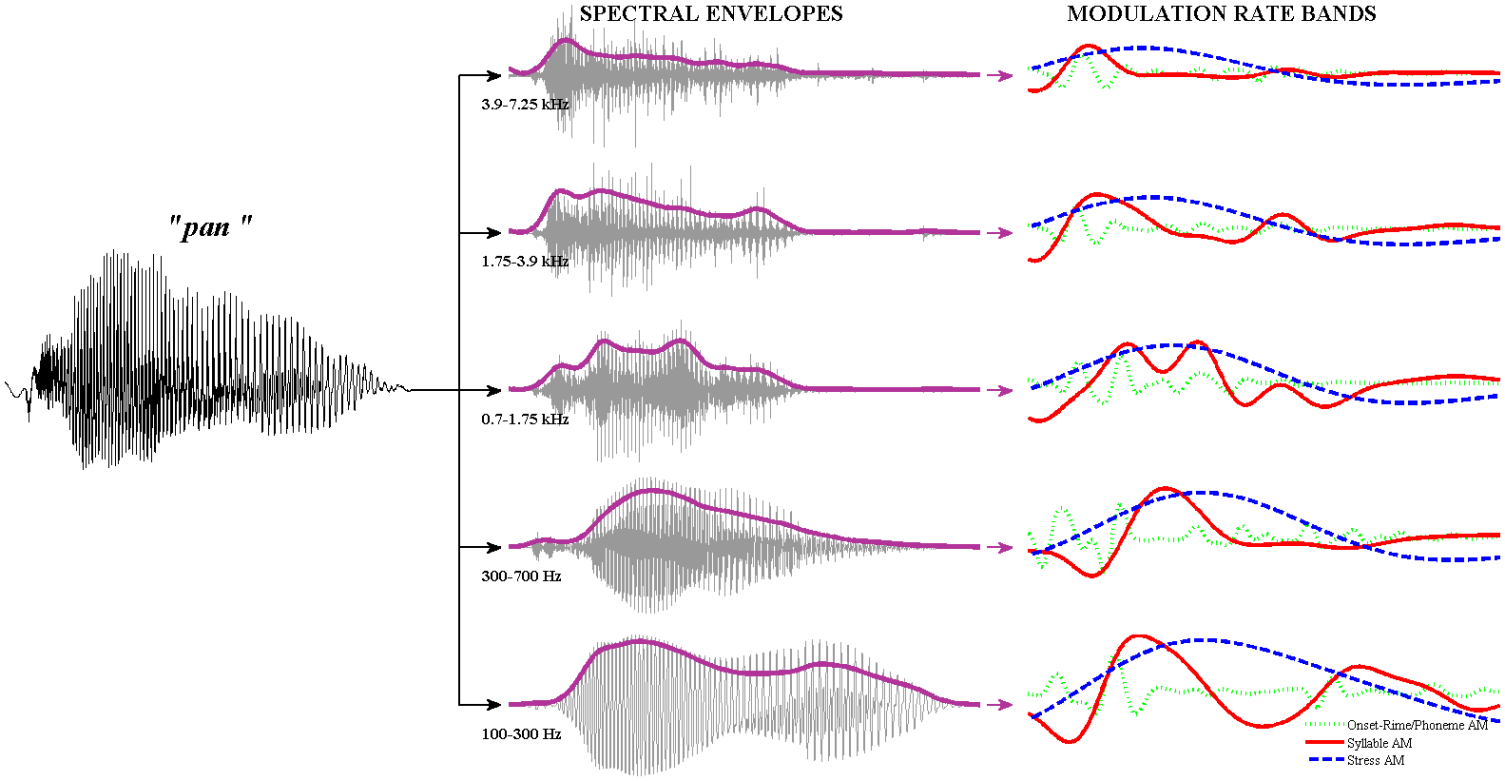
Example of the 5x3 spectro-temporal representation of speech modulation structure. (Left column) Original acoustic waveform for the word "pan". (Middle column) 5-spectral band filtered signal with Hilbert envelopes from each spectral band overlaid in bold. (Right column) Final 5x3 spectro-temporal representation. 3 modulation rate bands (Stress, Syllable & Phoneme) are extracted from each of the envelopes in the 5 spectral bands. Each modulation rate band is plotted in a different colour.

The Spectral Amplitude Modulation Phase Hierarchy Model (S-AMPH) model is based on a low-dimensional representation of CDS spectro-temporal modulation architecture. Derived from the combined results of the Spectral and Modulation Rate PCAs, this hierarchy consists of 5 spectral bands (100–300 Hz; 300–700 Hz; 700–1750 Hz; 1750–3900 Hz; 3900–7250 Hz), each containing 3 *hierarchically-nested* modulation rate bands (Stress AM: 0.2–2.5 Hz; Syllable AM: 2.5–12 Hz; Onset-rime/Phoneme AM: 12–40 Hz). The Matlab code for computing the S-AMPH 5x3 AM hierarchy is available as S1 Code. This 5x3 representation of core CDS spectro-temporal modulation structure is illustrated in Fig 9. In the spectral domain, the 5 core spectral bands could correspond to speech *features* such as fundamental frequency, formant structure, voicing or frication. In the temporal domain, the 3 major modulation rate bands show close correspondences to the characteristic timescales of 3 major types of phonological units: *prosodic feet*, *syllables* and *onset-rimes*.

To assess the generalisability of this 5x3 spectro-temporal banding structure to other types of spoken content and speaking styles, an identical PCA analysis was carried out with 3 other types of speech corpora, all obtained from the same 6 speakers. These additional corpora were (1) Children's nursery rhymes read in an adult-directed manner; (2) Children's stories read in a child-directed manner; and (3) Spontaneous adult-directed speech. The details and results of the PCA analyses with these additional corpora are provided as S4 Appendix, which shows that the 5x3 spectro-temporal banding structure (used in the S-AMPH model) is broadly conserved across both adult-directed and child-directed speech, although there are different emphases in different spectral and temporal regions for each speaking style. While the *biological* counterpart of this model, the neuronal oscillatory hierarchy, has already been proposed as a neurophysiological mechanism for parsing speech [33,34,36], there has not yet been a parallel acoustic demonstration that this hierarchy is actually present in the speech signal. Accordingly, a crucial test of the model's validity is whether it is able to act as an acoustic-phonological mapping framework for the naive listener.

### Principles of Acoustic-Phonological Structure Mapping by the S-AMPH Model

#### General Principles of Acoustic-Phonological Structure Mapping

Our results suggest that the core temporal modulation structure of CDS provides a *hierarchy* of AM patterns at 3 major timescales, ~ 2 Hz, 5 Hz and 20 Hz. Linguistically, this hierarchy could yield stress patterns (stress feet or proto-words, ~2 Hz), syllables (~5 Hz), and onsets (here, typically a single phoneme preceding the vowel nucleus, ~20 Hz). Accordingly, stress patterns, syllables and onset-rime units can be described as *acoustically-emergent* phonological units that can be parsed from the acoustic temporal structure of the AE via structure-mapping of the AM hierarchy by a naive listener. The S-AMPH model simulates this acoustic-phonological mapping process using two core principles, one based on correspondences between AM cycles and phonological units, and the other based on correspondences between oscillatory phase relationships and prosodic strength.

#### Acoustic-Phonological Mapping: From AM cycles to phonological units

In the AM hierarchy, each AM tier represents a different phonological grain size. These different grain sizes are the stress foot (top AM tier, slowest rate ~2 Hz), the syllable (middle AM tier, middle rate ~5 Hz), and the onset-rime unit, particularly here the *onset* (initial consonant or consonant cluster, bottom AM tier, fastest rate ~20 Hz). These grain sizes may be inferred from the different AMs because there is a direct *physical* correspondence between the characteristic duration (timescale) with which each type of phonological unit is uttered, and the equivalent modulation in energy produced by the utterance. For example, the typical duration of syllables in English spoken ADS is ~200 ms [21,51]. As CDS is produced at a slower rate, mean syllable durations are correspondingly longer, with estimates ranging from ~250 ms to ~350 ms [61–62]. Thus, each uttered syllable typically produces a momentary increase in speech energy (i.e. an AM) that lasts for around 200 ms (5 Hz, ADS) or 250–350 ms (~3.3 Hz, CDS). This correspondence allows us to make a reverse inference from the observed pattern of AM activity to the underlying phonological structure of the utterance. For example, it could be proposed that each single AM cycle within each AM tier corresponds to the occurrence of a single phonological unit at that grain size, so that a 1:1 mapping exists between AM cycles and phonological units.

#### Acoustic-Prosodic Mapping: From AM oscillatory phase to prosodic strength (S-w)

**Fig 10:**
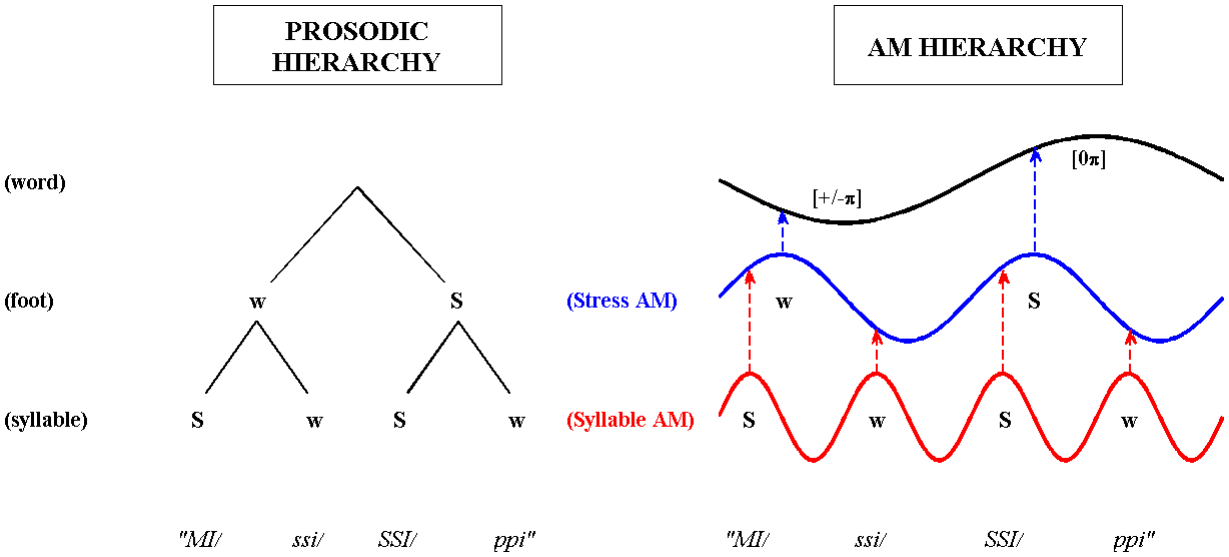
Representation of the prosodic hierarchy as an AM hierarchy. The dotted arrows represent the phase-relationships between nested units in the hierarchy, which indicate the prosodic prominence of daughter units ('S' or 'w'). For example, the 'w-S' iambic pattern of the 2 stress feet within the word is given by the phase relationship between the Stress AM and its upper parent tier, which takes the form [+/-π, 0π].

The AM hierarchy is also capable of representing the prosodic prominence or strength of its elements (i.e. Strong 'S' or weak 'w'). In a classic linguistic prosodic hierarchy [63–67], adjacent units at each level *alternate* in relative prominence. That is, each unit (e.g. syllable) can be either stronger or weaker than its neighbour, producing the characteristic strong-weak alternation that underlies English speech rhythm [68–69]. Thus, it is not the *absolute* amplitude of a syllable that determines its strength, but rather its *relative* amplitude in relation to its neighbours. To instantiate this concept of relativity, oscillatory ***phase*** is used as the computational statistic. By taking only the *phase series* of an AM pattern, one is effectively left with the relative pattern of amplitude change, disregarding any fluctuations in overall power. Thus, strong-weak rhythmic alternation patterns can conveniently be expressed in terms of a series of oscillatory phase states. Here, we use the phase convention where +/-1π radian refer equally to the oscillatory trough and 0π radians refers to the oscillatory peak. Thus, the trochaic [S,w] pattern can be expressed in phase terms as [0π, +/-π], whereas the iambic [w,S] pattern is expressed as [+/-π, 0π] (see Fig 10 for an illustration).

A second property of the prosodic hierarchy is that the prominence of units accrues in a top-down manner due to hierarchical *nesting* [66–67]. Fig 10 shows the hierarchically-nested prosodic structure for the word *"Mississippi"*. In this example, the 4 component syllables are nested within 2 stress feet, which in turn are nested within the whole word. One direct consequence of nesting is that the prosodic prominence of nested lower-level units (e.g. syllables) is governed by the prominence of parent units at a higher level in the hierarchy (e.g. stress feet). In the AM hierarchy, this top-down hierarchical control of prominence is instantiated in a scheme where the oscillatory phase of a *slower* AM governs the relative prominence of nested cycles of a *faster* AM. For example, as indicated by dotted arrows in Fig 10, Syllable AM cycles that occur near the peak [0π] of the Stress AM are prosodically-strong, whereas Syllable AM cycles that occur near the trough [+/-π] of the Stress AM are prosodically-weak.

### Evaluation of S-AMPH Acoustic-Phonological Structure Mapping

The efficacy of acoustic-phonological structure-mapping by the S-AMPH model was evaluated using 2 sets of CDS. The first was rhythmically-timed speech with a regular (isochronous) beat pattern (Nursery Rhymes Corpus 2, see Methods), such as might be produced when parents sing or play hand-clapping games with their children. The second was freely-produced but child-focussed speech, such as parents might produce when telling nursery rhymes and stories to their children (i.e. speaking in CDS without an external time-keeper; Nursery Rhyme Corpus 3, see Methods). Developmentally, it is plausible that deliberately-timed speech may contain more strongly phase-locked hierarchical modulation patterning, which should enable better extraction of phonological structure by naïve listeners (yielding a rhythmic benefit).

For the interested reader, S5 Appendix provides a detailed breakdown of the model's performance. This Appendix shows the percentage of hits, misses, correction rejections and false alarms computed for each phonological level (syllable, onset-rime and prosodic stress), for each speech corpus separately (timed or untimed) and for each type of metrical patterning (bi-syllable- or tri-syllable-footed). A summary of the key performance indicators (accuracy and d') is shown in Table 7. Inspection of Table 7 shows that the accuracy of acoustic-phonological mapping achieved by the S-AMPH model (averaging hits and correct rejection rates) exceeded 72% for onset-rime detection, syllable-finding and stress detection. The best performance was observed for syllable-finding in timed CDS (98% accuracy, d' = 4.3), and the weakest performance was observed for prosodic stress detection in untimed CDS (72% accuracy, d' = 1.2). Notably, the model performed significantly better for deliberately timed speech than for untimed speech in all cases (gains of between 13%-22%, see S5 Appendix for details of statistical tests). For timed speech, >90% accuracy was achieved for all phonological levels. For untimed speech, between 72%~82% accuracy was achieved across the three phonological levels. This difference is consistent with experimental evidence showing that the use of rhythmically-regular speech (as in sung nursery rhymes and hand-clapping games) with children significantly improves their phonological awareness [70–71]. In principle, therefore, the language routines of early childhood could potentially increase the accuracy of the child's phonological parsing by as much as ~15% (for syllables and onset-rime units) and by over 20% (for prosodic stress patterns). However, it remains to be investigated whether young children can track and use acoustic modulation patterns at a level comparable to adults [25,33]. Moreover, it is also unknown whether these mechanisms would be effective in parsing less rhythmically-regular forms of speech.

**Table 7:** Summary performance of the S-AMPH model. The table shows accuracy of performance for acoustic-phonological mapping at each phonological level for timed and untimed child-directed speech, and the advantage for timed-untimed CDS. The mean and standard deviation of the distance of the models' estimates from their respective phonological targets is also included.

		Timed CDS	Untimed CDS	Timed-Untimed
**Syllable**	*Accuracy*	98.3%	81.8%	16.5%
	*d'*	4.33	1.82	2.51
	*Distance from target (SD)*	22.8 ms (21.4 ms)	7.52 ms (30.7 ms)	15.28 ms
**Onset-Rime**	*Accuracy*	90.9%	77.6%	13.3%
	*d'*	2.76	1.53	1.23
	*Distance from target (SD)*	-1.80 ms (10.9 ms)	1.45 ms (11.2 ms)	-3.33 ms
**Prosodic Stress**	*Accuracy*	94.8%	72.4%	22.4%
	*d'*	3.46	1.21	2.25

## Discussion

In this paper, we developed a computational model, the S-AMPH model, to investigate the efficacy with which a naive learner could perform acoustic-phonological structure-mapping using only the modulation architecture and statistics of child-directed speech. We showed that the core acoustic modulation structure and statistics of the amplitude envelope of CDS form an inherent oscillatory hierarchy, whose statistics can be used to extract latent large-grain phonological structure (stress patterns [stress feet or proto-words], syllables and onset-rime units) by a process of structure-mapping with good accuracy (minimum of 70% across the three phonological levels). Theoretically, this structure-mapping process is mediated by the automatic entrainment (phase-locking) of endogenous neuronal oscillations to acoustic modulation patterns in CDS (i.e. delta-to-Stress AM, theta-to-Syllable AM). The result is Acoustic-Emergent Phonology, signal-driven parsing of CDS into emergent phonological units that could *in principle* provide the earliest building blocks for a young child's phonological system. Although it remains to be shown empirically that young children can track and use these acoustic modulation patterns as the S-AMPH model does, our modelling specifies biologically plausible developmental mechanisms that may contribute to the development of a phonological system by young children. AEP theory also suggests an acoustic basis for linguistic descriptions of hierarchical prosodic structure in the English language [63–67].

AEP theory draws on emerging evidence that nested oscillatory hierarchies are relational structures that characterise both sensory inputs (as demonstrated here for CDS) and neural encoding mechanisms (as demonstrated by Poeppel [36], Gross [35]). Work with both primate and human participants has demonstrated that endogenous neuronal oscillations within the auditory cortex fluctuate on similar timescales to the modulation patterns in the speech signal (notably delta, theta and gamma). Auditory neuronal oscillations are hierarchically-nested through phase-phase and phase-power couplings [41,35–36]. We have also previously demonstrated the perceptual utility of such oscillatory nesting between AM rates in the speech signal [25]. We demonstrated a key role for delta-theta phase alignment in prosodic prominence. When adults were asked to perform a rhythm discrimination task with degraded speech, they showed perceptual sensitivity to the phase coupling/nesting between the Stress AM and the Syllable AM, and used this hierarchical coupling to infer prosodic 'Strong'-'weak' stress patterns. Further investigations are now needed to determine whether the nested AM oscillations in the speech signal are tracked rate-wise by nested neuronal oscillations in auditory cortex, and whether the fidelity of this neural tracking predicts the accuracy of participants’ hierarchical phonological parsing of the speech signal. We now compare the S-AMPH to other models, and briefly assess the implications of AEP theory for children’s language development, for developmental disorders of language, for the classification of languages by rhythm type, and for the analysis of inherently rhythmic signals such as music.

### Comparison with Other Models

The S-AMPH model follows a long tradition of computational models whose aim is to describe the process by which listeners transform acoustic landmarks in the speech signal into distinctive phonological features that support speech comprehension (e.g. [72]). In terms of syllable-finding, the performance of the S-AMPH model compares favourably to that of other computational methods designed specifically for syllable detection. However, it should be noted that these other models have only been implemented in adult-directed speech. Thus, whilst analogies may be drawn between the performance of the S-AMPH model and these other computational models, direct comparisons may not be appropriate as these methods have not been applied to child-directed speech nor has our new method been tested on adult-directed speech. Nonetheless, the current AM peak detection method is, in principle, similar to that used by Pfitzinger et al [73], who also used peaks in the low-pass filtered (~<10 Hz) envelope as candidates for syllables. Pfitzinger et al [73] reported achieving accuracy rates of 87% and 79% for read and spontaneous adult-directed speech respectively, where detection was deemed accurate when the candidate syllable lay within 100 ms of the target. These percentages are not distant from the S-AMPH accuracy rates of 98% and 82% for timed and untimed child-directed speech respectively, using a similar criterion threshold of half a syllable length (varying between 70–167 ms). More sophisticated supervised machine-learning methods (e.g. [74–75]) achieve similar syllable detection accuracy rates of ~88% for read speech. These mapping results with syllables support our proposal that the acoustic modulation structure of CDS naturally contains strong acoustic cues for the extraction of large grain size phonology (Acoustic-Emergent Phonology).

With regard to onset-rime detection, the most relevant computational models are those that have been developed to detect the perceptual-centre (P-centre), or ‘moment of occurrence’ of an auditory event in any sensory modality [76–77]. P-centres in speech are commonly associated with the beginnings of syllable vowel nuclei, or the onset-rime division [78–81]. The P-centre of a sound is also its rhythmic centre. For example, when speaking the syllables "SWEET" and "SEAT" in time to a regular metronome beat, one would align the nucleus of their rimes ("-EET" and "-EAT") to time with the pacing beat, rather than their onsets ("SW-" and "S"). Consequently, for any particular beat interval, one would have to begin saying "SW-EET" earlier than "S-EAT" in order to ensure that both rimes were produced to coincide with the metronome beat. Although the exact location of P-centres in individual syllables can vary depending on the consonants that precede and follow the vowel nucleus (i.e. the phonological characteristics of onsets and codas), it is generally agreed that acoustically, P-centres are cued primarily by changes in loudness or signal amplitude [78–81]. Accordingly, attempts to identify and model the acoustic correlates of P-centres in speech have focused on the speech amplitude envelope.

For example, Howell [82–84] proposed a syllabic center of gravity model, in which the distribution of energy within the amplitude envelope was the key determinant of P-centre location. Other models by Pompino-Marschall [54] and Harsin [55] make use of loudness functions or the rate-of-change of modulation in the envelopes of different spectral bands. The approach we adopt here is similar to that by Pompino-Marschall [54] and Harsin [55], whereby a modulation rate-of-change function is used to model the onset-rime division within syllables (see Methods). It is difficult to compare the performance of the S-AMPH model in onset-rime detection against that of existing P-centre models for two reasons. First, P-centre models have primarily been tested on single isolated syllables or sounds, rather than on connected speech (as was done here). To our knowledge, there have been no corpus-based assessments of P-centre models. Second, P-centre models describe a *perceptual* phenomenon (the subjective moment of occurrence of a sound) whereas the S-AMPH attempts to detect an acoustic landmark (the onset-rime division) which is closely related to, but not synonymous with, the P-centre of a sound. Nonetheless, a comparative assessment by Collins [85] on eight different P-centre models using a varied database of 100 single short sounds (including musical instruments, speech, song and sine tones) found that on average, the mean error per sound over all 8 P-centre models was 65.3 ms. That is, on average, the models could only predict the P-centres of ~50% of the stimuli (assuming that performance over stimuli was normally distributed) within 65 ms of their actual location (which was determined experimentally). By comparison, the S-AMPH model located 91% of onset-rime divisions within 30 ms (for the timed corpus, which was more similar to Collins' single short stimuli). Thus, we conclude that the performance of the S-AMPH model compares favourably to that of existing P-centre models.

In terms of mapping between acoustic and prosodic structure, the S-AMPH model performed at a comparable level to other automatic stress transcription models. For example, Silipo & Greenberg [86] tested a variety of models using amplitude, duration and pitch cues where these cues were either used singly or in paired combination. Silipo & Greenberg [86] reported that the best performance was obtained when *both* duration and amplitude cues were used in combination, yielding correct identification of ~80% of stressed syllables and ~78% of unstressed syllables in adult-directed speech. When amplitude cues alone were used, transcription accuracy was ~64% for stressed syllables and ~65% for unstressed syllables. The 72% accuracy achieved by the S-AMPH model for untimed speech, using amplitude cues only, is thus slightly better. Clearly, other acoustic cues such as duration and pitch are also available to children, and would support the identification of prosodic stress in the speech signal. Nevertheless, our modelling suggests that the AM statistics of the AE already allow listeners to identify over 70% (and potentially up to 95%) of the stressed and unstressed syllables in speech correctly, even without other acoustic cues.

### Prosodic Structure in CDS and Language Development

The AEP model fits well with cross-language data showing that prior to formal literacy instruction, pre-reading children already possess reflective awareness of phonological units at the larger grain sizes of syllables and onset-rime units [1–2,87]. AEP theory demonstrates how this explicit awareness could develop in part from emergent features of the acoustic signal of CDS. Furthermore, AEP theory addresses a gap in the current literature, which is that there is currently no neural mechanistic description of how infants first acquire phonological knowledge. Despite longstanding recognition of the importance of bootstrapping in early language learning [52,88–89], AEP is the first theory to provide a statistical description of the precise temporal properties of the speech signal that might support the extraction of phonological structure. Thus, AEP theory potentially offers a significant advance in our understanding of the sensory/neural mechanisms of early language acquisition and phonological development, and can be extended to infants (e.g., [23]). By demonstration, we have used the S-AMPH model to analyse the temporal modulation patterns in infant-directed speech (IDS) as spoken live to typically-developing infants at 7-, 9-, and 11-months of age [23]. Our S-AMPH analysis revealed that compared to ADS, the modulation peak in IDS was shifted toward the slower (delta) modulation rate (a “stress-shifted” pattern). This “stress-shifted” rhythmic profile should support delta-rate neuronal entrainment to the speech signal by infants, which could be adaptive in helping infants to locate word boundaries during early language acquisition. Electrophysiology (EEG) reveals awareness of trochaic patterns by 4–5 months of age [90]. Nevertheless, it is risky to compare oscillations and timed responses in infants and children with those of adults. Further studies specifically testing AEP theory need to be performed with infants and children in order to document the nature of these sensory-neural mechanisms.

### Implications for Developmental Language Disorders

AEP theory demonstrates that the speech amplitude envelope contains vital acoustic structure relevant to phonological parsing. Therefore, children with poor sensitivity to amplitude envelope structure would be at a disadvantage during early phonological development. Such children would be less efficient at detecting prosodic strength, at parsing syllables and at locating onset-rime units in the speech input. Consequently the auditory organization of their mental lexicons would be atypical, with the emergent phonological structure in the amplitude envelope poorly represented [8]. This developmental description may capture the acoustic basis of developmental dyslexia [4]. In EEG studies the greatest perceptual difficulties in dyslexia occur with slower rise times [91], while psychophysical studies show that whereas 9-year old children without dyslexia can distinguish amplitude rise time differences of ~46 ms, 11-year-old children with dyslexia reading at a 9-year old level have a threshold of ~114 ms [92]. Sensory difficulties with slower rise times may explain why non-speech rhythm perception is also impaired in children with dyslexia [93–94]. Children with specific language impairment (SLI) also show rise time discrimination impairments and reduced prosodic sensitivity [95]. Since both developmental dyslexia and SLI are congenital disorders, infants at high genetic risk for developing dyslexia or SLI should in theory already show impaired sensitivity to amplitude rise time as well as atypical oscillatory mechanisms, particularly in the crucial delta (Stress AM) and theta (Syllable AM) bands. This presents the possibility that impaired rise time discrimination and atypical oscillatory activity could act as early biological markers for infants at-risk for developing later language disorders.

### Cross-Linguistic Perspectives: AEP Theory and Rhythm Typologies

The acoustic basis of language rhythm typologies continues to be debated [96]. Our findings suggest that rhythm typologies could be associated with the acoustic *modulation statistics* of CDS that are learned during infancy and childhood. A core modulation statistic for the S-AMPH is oscillatory phase. Phase-synchronicity is distinct from durational isochrony because two oscillators can remain phase-locked (synchronised) even when they are stretched or compressed in duration (e.g. as speakers slow down or speed up). When the Stress- and Syllable-rate modulations in speech are phase-synchronised, the resulting percept is that of a regular rhythmic pattern of stressed and unstressed syllables [97], which confers high transitional predictability to the utterance. Through language exposure, English-learning children’s perceptual systems may become hard-wired to expect and extract regularities in speech at these stress-syllable timescales. Consequently, during adulthood, English listeners may *impose* a framework of stress-syllable regularity when listening to speech, even though the speech signal itself (i.e. ADS) does not contain strong temporal regularity. This could explain why English is perceived to be regularly 'stress-timed', even though durational isochrony is absent from the (adult-directed) speech signal. By extension, the algorithms used in the S-AMPH could also offer new perspectives on the rhythm typology of other languages. For languages with a different rhythmic organisation, we might predict that the critical phase axis may not be the Stress-Syllable AM axis identified here, but rather phase relations between faster AM rates, in line with the phonological unit used by a particular language as the basic unit of rhythm. It is also possible that in fact all human languages can be arranged along a continuum as being “more-or-less stress-timed” [27]. In this case, the number and configuration of tiers within the AM hierarchy that are used to specify rhythm should systematically predict the location of a given language on this continuum.

### The S-AMPH Model and Rhythmic Structure in Music

Language and music may result from general perceptual mechanisms that are neither music- nor language-specific [69,98]. In principle, the application of spectral and modulation rate PCAs to different types of music should therefore also reveal covert hierarchical structures of the kind found here for the speech signal. Tapping a finger to a metronome beat, tapping one’s foot to the beat of music, and musicians playing different instruments together in time all depend on accurate perception and prediction of underlying rhythm. Different instruments produce notes with different rise times (attack times, see [99]), hence musicians playing different instruments who entrain together must phase-align the rise times of their notes in order to keep accurate time. Slower temporal rates at the top of the AM hierarchy are more likely to drive this mutual entrainment behaviour than faster temporal rates [97], and this is likely to involve the delta rate (in music the rate of 1.67 Hz, or a 600 ms interval, is frequently designated the preferred or natural tempo [100]). The rhythmic structure of music and language are aligned when we sing, and therefore training that assists children in being sensitive to the accuracy of this alignment should also help children to develop better phonological skills [70].

### The S-AMPH Model and Neural Speech Encoding

The modelling also fits well with current neural models of speech encoding by adults [32,34,36], as highlighted in the Introduction. Automatic neuronal entrainment mechanisms at rates matching the temporal patterns in the AE (delta, theta, beta, gamma; see [35–36]) may enable parsing of the speech signal by a signal-driven process of structure-mapping (mapping of the acoustic hierarchy onto the oscillatory hierarchy). For example, Poeppel and colleagues have demonstrated in adult MEG studies that theta oscillations phase-entrain to the peaks and troughs in acoustic modulation patterning at the syllable rate, and the fidelity of this neuronal entrainment predicts speech intelligibility [33]. Mechanistically, the beginnings of new phonological units would typically be identifiable by prominent increases in the amplitude of the signal (i.e. large rise times), which could function perceptually as auditory temporal edges [35,101]. These edges would both delineate new auditory objects (e.g. rime units) and re-set endogenous neuronal oscillations, enabling phase-alignment and neuronal entrainment. Regarding studies with children, the S-AMPH suggests that delta-driven parsing may be equally or more important for accurate speech encoding than theta-driven parsing. Indeed, we have demonstrated neuronal entrainment in the theta and delta bands in children to syllables produced rhythmically at a 2 Hz rate, and have also demonstrated *atypical* oscillatory delta entrainment for children with dyslexia [102–103]. Neuronal entrainment to speech stimuli is therefore present in childhood, and provides a sensory/neural mechanism for the extraction of Acoustic-Emergent Phonology (AEP) by young listeners.

### Limitations of Current Model

Acoustic-Emergent Phonology theory has several limitations. First, the S-AMPH model does not take into account the contribution of phonetic and phonotactic cues, or top-down lexical effects, in emergent phonological development. Secondly, as the amplitude envelope is the focus of AEP theory, any prosodically-relevant information that is present within the fine structure of the signal (e.g. pitch accent cues) is not accounted for. As a result of these two limitations, it is possible that AEP theory might underestimate children’s true capabilities in extracting phonological information bottom-up from the speech signal. On the other hand, the current model is based on a select corpora of nursery rhymes uttered by experienced female early years educators, thereby comprising high-quality CDS. Thus it is at present unclear how far the model may be generalised across other forms of CDS. It should also be noted that the speech corpus used for the statistical modelling comprised of read (i.e. scripted), rather than truly spontaneous child-directed speech (although a PCA analysis of spontaneous adult-directed speech yielded similar spectro-temporal structure to read CDS, see S3 Appendix). It is possible that mothers' (and fathers') utterances might differ subtly in temporal structure, depending on whether the produced speech is scripted or part of natural conversation. Nevertheless, the nursery rhyme materials used in this study were chosen from popular children's literature, hence similar material would realistically be heard by the child at home and in nursery. Furthermore, ongoing analysis of IDS shows that Stress-rate modulations dominate the modulation spectrum even more in spontaneous speech to infants [23]. Hence the use here of scripted speech may not exert strong effects. Further experimental research is clearly required to evaluate the hypotheses put forward here, ideally across languages, to investigate these and related issues in greater depth.

Finally, it should be noted that the focus of the S-AMPH model is on the *parsing* of phonological units (i.e. where is a syllable?), rather than on the identification or discrimination of these units per se (i.e. is this syllable "cat" or "cap"?). Consequently we primarily make use of modulation patterns in the *rate* dimension (which specify units), rather than modulations in the spectral dimension (which could specify features). Note also that even higher-order (slower) AM tiers than used here can be present in adult-directed speech (e.g. corresponding to phrases). In the spectral domain, the 5 core spectral bands could plausibly correspond to speech features such as fundamental frequency, formant structure, voicing or frication. In future versions of the model, featural computations could be included to yield a fully-fledged phonological model of speech units as well as their distinctive features.

### Conclusion

In summary, we show here that the AE of CDS can be described statistically in terms of an acoustic AM hierarchy that would match the known neural oscillatory hierarchy. Rate-specific and automatic neuronal entrainment to this acoustic hierarchy by infants and children would enable automatic parsing of the speech signal, yielding a set of phonological primitives (Acoustic-Emergent Phonology). Social learning mechanisms and top-down processes could capitalise upon these acoustic-emergent units to build a phonological system. In principle, social learning mechanisms, language play and top-down (e.g. semantic and pragmatic) processing would complement signal-driven parsing and facilitate the development of phonological representations that code prosodic structure, syllable boundaries and onset-rime information by pre-school children, prior to the teaching of literacy. Individual differences in sensitivity to the AM structure of speech would be expected to be associated with individual differences in children’s phonological awareness, as is empirically the case [3,4,8]. Application of the S-AMPH model to CDS in languages other than English awaits further research. Such investigations would enable a series of empirical tests of AEP theory.

## Supporting Information

S1 Appendix CDS Corpus 1 metadata, stimulus description & acoustic parameters. (DOCX)

S2 Appendix Root-mean-square (RMS) power and cross-correlation across spectral channels. (DOCX)

S3 Appendix Individual speakers' rectified loading patterns for the spectral PCA. (DOCX)

S4 Appendix Results of PCA analyses with other speech corpora (CDS and ADS). (DOCX)

S5 Appendix Detailed breakdown of S-AMPH performance at each phonological level. (DOCX)

S1 Code S-AMPH Matlab code and functions. (DOCX)

## Abbreviations

AE: Amplitude envelope
AEP: Acoustic-emergent phonology
AM: Amplitude modulation
ADS: Adult-directed speech
CDS: Child-directed speech
ERB: Equivalent rectangular bandwidth
FM: Frequency modulation
PCA: Principle Component Analysis
PC: Principle component
PSM: Prosodic strength measure
RMS: Root-mean-square
S-AMPH: Spectral Amplitude Modulation Phase Hierarchy
'S': Strong syllable
'w': weak syllable
