# Supplementary material for: Acoustic-Emergent Phonology in the Amplitude Envelope of Child-Directed Speech
Source: PLoS One. 2015 Dec 7;10(12):e0144411. doi: 10.1371/journal.pone.0144411 (PMC4671555; doi:10.1371/journal.pone.0144411)
Supplement: S1 Appendix — (DOCX) [file pone.0144411.s001.docx]

**Metadata, Stimulus Description & Acoustic Parameters for CDS Corpus 1**

CDS Corpus 1 can be accessed online using the following weblink :

http://figshare.com/articles/SAMPH_CDS/1318572

DOI : 10.6084/m9.figshare.1318572

License : CC-BY

Total Filesize : 626.20 MB

File format : .wav

Sampling rate : 44.1 kHz

This CDS corpus was used to derive the SAMPH model, a hierarchical model of acoustic-emergent phonological structure. The corpus comprises 44 nursery rhymes that were each read aloud by 6 female speakers (total of 264 samples). File names are concatenations of the speaker number (01-06) followed by the nursery rhyme number (01-44), followed by the suffix 'C' (for child-directed speech). Thus, file '0101C.wav' refers to nursery rhyme #1 spoken by speaker #1. Table a lists the file numbers of the nursery rhymes and their rhythmic meter.

**Table a. Nursery Rhyme List**

| File No | Nursery Rhyme | Music Time Signature | Rhythmic Meter |
| --- | --- | --- | --- |
| 1 | Baa Baa Black Sheep | 2/4 | Duple |
| 2 | Once I Caught a Fish Alive | 4/4 | Duple |
| 3 | One Two Buckle my Shoe | 4/4 | Duple |
| 4 | Old MacDonald Had a Farm | 4/4 | Duple |
| 5 | Twinkle Twinkle Little Star | 4/4 | Duple |
| 6 | London Bridge is Falling Down | 4/4 | Duple |
| 7 | Mary Had a Little Lamb | 4/4 | Duple |
| 8 | Polly Put the Kettle On | 2/4 | Duple |
| 9 | Yankee Doodle | 2/4 | Duple |
| 10 | Peter Peter Pumpkin Eater | 4/4 | Duple |
| 11 | Mary Mary Quite Contrary | 4/4 | Duple |
| 12 | Simple Simon Met a Pieman | 4/4 | Duple |
| 13 | As I Was Going to St Ives | 2/4 | Duple |
| 14 | The Queen of Hearts | N.A. | Duple |
| 15 | Lucy Lockett | 4/4 | Duple |
| 16 | Cobbler Cobbler Mend My Shoe | 4/4 | Duple |
| 17 | Peter Piper | N.A. | Duple |
| 18 | I'm a Little Teapot | 4/4 | Duple |
| 19 | Sing a Song of Sixpence | 2/4 | Duple |
| 20 | Wee Willie Winkie | 2/4 | Duple |
| 21 | Old King Cole | 4/4 | Duple |
| 22 | The Wheels on the Bus | 4/4 | Duple |
| 23 | Three Little Monkeys | N.A. | Duple |
| 24 | Grand Old Duke of York | 4/4 | Duple |
| 25 | Incy Wincy Spider | 6/8 | Duple |
| 26 | Jack and Jill | 6/8 | Duple |
| 27 | Humpty Dumpty | 6/8 | Duple |
| 28 | Ring-a-Ring-a-Roses | 6/8 | Duple |
| 29 | Row Row Row Your Boat | 6/8 | Duple |
| 30 | Hickory Dickory Dock | 6/8 | Duple |
| 31 | Here We Go Round the Mulberry Bush | 6/8 | Duple |
|  |  |  |  |
| 32 | Little Miss Muffet | 6/8 | Triple |
| 33 | Little Jack Horner | 6/8 | Triple |
| 34 | Little Boy Blue | 6/8 | Triple |
| 35 | Curly Locks | 6/8 | Triple |
| 36 | To Market | 6/8 | Triple |
| 37 | Pussycat Pussycat | 6/8 | Triple |
| 38 | Ladybird Ladybird | 6/8 | Triple |
| 39 | There Was An Old Lady | 6/8 | Triple |
| 40 | Two Cats of Kilkenny | N.A | Triple |
| 41 | Ride a Cock Horse | 3/4 | Triple |
| 42 | Orange and Lemons | 3/4 | Triple |
| 43 | Rock-a-Bye-Baby | 3/4 | Triple |
| 44 | Lavender's Blue | 3/4 | Triple |

Note that some nursery rhymes with an assigned with a 'Duple' or 'Triple' meter actually had a compound musical time signature, such as 6/8. Compound time signatures consist combinations of duple or triple beats within each bar, for example 6/8 indicates 2 sets of triple beats. Therefore, these rhymes can be uttered to fit a duple meter as well a triple meter, depending on the rate of speaking. In these cases, the decision as to whether a rhyme was 'duple'- or 'triple'-meter was made on the basis of poetic scansion, using the dominant prosodic foot length.

From inspection of the table, 31 out of the 44 (70%) nursery rhymes were assigned a duple meter, while 13 (30%) were assigned a triple meter. The fact that all the nursery rhymes had relatively short prosodic feet (2 or 3 syllables in length) is consistent with Gueron's (1974) analysis of the metrical structure of 130 Mother Goose nursery rhymes. She concluded that all but one of the nursery rhymes had a simple 'Strong (S) - weak (w)' alternating metrical pattern of : (w) S w S (w) S w S (w), with the weak elements in parenthesis omitted in some rhymes. In Gueron’s analysis, 'S' elements were usually realized by a single stressed syllable while 'w' elements were realised by between one to three unstressed syllables. Consequently, the prosodic feet in Gueron's analysis had a maximum length of 4. While the relative frequencies of each type of prosodic foot were not given in the study, the current set of nursery rhyme material indicates a higher incidence of nursery rhymes with shorter (e.g. 2-syllable-long) prosodic feet.

Acoustic Parameters

The following acoustic parameters were measured for this CDS corpus and shown in the table below : maximum intensity (dB SPL), mean intensity (dB SPL), maximum pitch (Hz) and mean pitch (Hz). For comparison, each speaker also produced conversational samples of adult-directed speech (see Appendix 4 for details).

**Table b. Acoustic parameters for CDS and ADS produced by the same speakers**

| **Corpus** | **Speaker** | **Max Intensity**  **(dB SPL)** | **Mean Intensity**  **(dB SPL)** | **Max Pitch (Hz)** | **Mean Pitch (Hz)** |
| --- | --- | --- | --- | --- | --- |
| CDS | 01 | 76.1 | 54.6 | 524.1 | 225.0 |
|  | 02 | 82.1 | 61.4 | 452.7 | 190.5 |
|  | 03 | 80.4 | 59.4 | 420.9 | 197.1 |
|  | 04 | 83.6 | 60.8 | 488.0 | 196.9 |
|  | 05 | 82.7 | 57.1 | 465.6 | 193.9 |
|  | 06 | 71.4 | 48.9 | 474.0 | 198.9 |
|  | ***Mean*** | ***79.4*** | ***57.0*** | ***470.9*** | ***200.4*** |
| ADS | 01 | 69.0 | 50.2 | 460.4 | 173.2 |
|  | 02 | 85.9 | 64.5 | 464.5 | 155.8 |
|  | 03 | 76.3 | 55.9 | 390.4 | 160.9 |
|  | 04 | 77.1 | 57.3 | 399.9 | 174.2 |
|  | 05 | 75.5 | 54.2 | 416.9 | 176.7 |
|  | 06 | 70.3 | 49.5 | 433.7 | 172.9 |
|  | ***Mean*** | ***75.7*** | ***55.3*** | ***427.6*** | ***169.0*** |

These children's nursery rhymes were selected on the basis of familiarity and metrical patterning. 22 nursery rhymes followed a duple rhythmic meters and a further 22 nursery rhymes followed a triple rhythmic meter, as shown in Table b.

Full Nursery Rhyme Text

**1. Baa Baa Black Sheep (x2)**

Baa baa black sheep have you any wool?

Yes sir, yes sir, three bags full.

One for the master and one for the dame

And one for the little boy who lives down the lane.

**2. Once I Caught a Fish Alive (x2)**

One, two, three, four, five

Once I caught a fish alive.

Six, seven, eight, nine, ten

Then I let it go again

Why did you let it go?

Because it bit my finger so

Which finger did it bite?

This little finger on my right.

**3. One, Two Buckle My Shoe**

One, two, buckle my shoe

Three, four, knock at the door

Five, six, pick up sticks

Seven, eight, lay them straight

Nine, ten, a big fat hen

Eleven, twelve, dig and delve

Thirteen, fourteen, maids a' courting

Fifteen, sixteen, maids in the kitchen

Seventeen, eighteen, maids in waiting

Nineteen, twenty, my plate's empty.

**4. Old MacDonald Had a Farm (x2)**

Old Macdonald had a farm, E-I-E-I-O

And on that farm he had some cows, E-I-E-I-O

With a moo-moo here and a moo-moo there

Here a moo, there a moo, everywhere a moo-moo

Old Macdonald had a farm, E-I-E-I-O

**5. Twinkle Twinkle Little Star (x2)**

Twinkle, twinkle, little star

How I wonder what you are

Up above the world so high

Like a diamond in the sky

Twinkle, twinkle, little star

How I wonder what you are!

**6. London Bridge**

London Bridge is falling down,

Falling down, falling down.

London Bridge is falling down,

My fair lady.

Build it up with wood and clay,

Wood and clay, wood and clay.

Build it up with wood and clay

My fair lady

Wood and clay will wash away,

Wash away, wash away.

Wood and clay will wash away,

My fair lady.

**7. Mary Had a Little Lamb**

Mary had a little lamb

its fleece was white as snow.

And everywhere that Mary went

the lamb was sure to go.

It followed her to school one day,

that was against the rule.

It made the children laugh and play

to see a lamb at school.

And so the teacher turned it out

but still it lingered near.

And waited patiently about

till Mary did appear.

Why does the lamb love Mary so?

The eager children cry.

Why, Mary loves the lamb you know,

the teacher did reply.

**8. Polly Put the Kettle On (x2)**

Polly put the kettle on,

Polly put the kettle on,

Polly put the kettle on

We'll all have tea

Sukey take it off again,

Sukey take it off again,

Sukey take it off again

They've all gone away!

**9. Yankee Doodle came to London (x3)**

Yankee Doodle came to London

Riding on a pony

He stuck a feather in his hat

And called it 'macaroni'!

**10. Peter Peter Pumpkin Eater (x3)**

Peter, Peter, pumpkin eater

Had a wife and couldn't keep her.

Put her in a pumpkin shell

And there he kept her very well!

**11. Mary Mary Quite Contrary (x3)**

Mary, Mary, quite contrary

How does your garden grow?

With silver bells and cockle shells

And pretty maids all in a row

**12. Simple Simon (x2)**

Simple Simon met a pieman

Going to the fair

Says Simple Simon to the pieman

"Let me taste your ware"

Says the pieman to Simple Simon

"Show me first your penny"

Says Simple Simon to the pieman

"Indeed I have not any"

**13. As I was going to St Ives (x2)**

As I was going to St. Ives

I met a man with seven wives

Each wife had seven sacks,

each sack had seven cats,

Each cat had seven kits.

Kits, cats, sacks and wives,

How many were going to St. Ives?

**14. The Queen of Hearts**

The Queen of Hearts

she made some tarts

all on a summer's day.

The Knave of Hearts

he stole those tarts

and took them clean away.

The King of Hearts

called for the tarts

and beat the Knave full sore.

The Knave of Hearts

brought back the tarts

and vowed he'd steal no more.

**15. Lucy Lockett (x3)**

Lucy Locket lost her pocket,

Kitty Fisher found it.

Not a penny was there in it,

only ribbon round it.

**16. Cobbler cobbler mend my shoe (x3)**

Cobbler, cobbler, mend my shoe.

Get it done by half past two.

Half past two is much too late,

get it done by half past eight!

**17. Peter Piper (x2)**

Peter Piper picked a peck of pickled peppers.

A peck of pickled peppers Peter Piper picked.

If Peter Piper picked a peck of pickled peppers,

Where's the peck of pickled peppers Peter Piper picked?

**18. I'm a Little Teapot (x3)**

I'm a little teapot short and stout.

Here's my handle, here's my spout.

When the kettle's boiling hear me shout:

Tip me up and pour me out!

**19. Sing a Song of Sixpence**

Sing a song of sixpence

A pocket full of rye

Four and twenty blackbirds

Baked in a pie

When the pie was opened

The birds began to sing

Wasn't that a dainty dish

to set before the King?

The King was in his counting house

Counting out his money

The Queen was in the parlour

Eating bread and honey

The maid was in the garden

hanging out the clothes

When down flew a blackbird

and pecked off her nose.

**20. Wee Willie Winkie (x2)**

Wee Willie Winkie

Runs through the town

Upstairs and downstairs

In his nightgown.

Rapping at the window,

Crying through the lock.

Are the children in their beds?

For now it's eight o'clock.

**21. Old King Cole**

Old King Cole was a merry old soul

And a merry old soul was he.

He called for his pipe and he called for his bowl

and he called for his fiddlers three.

Every fiddler he had a fiddle

and a very fine fiddle had he.

Oh, there's none so rare as can compare

with King Cole and his fiddlers three.

**22. The Wheels on the Bus**

The wheels on the bus go round and round,

Round and round, round and round.

The wheels on the bus go round and round

All day long.

The horn on the bus goes beep, beep, beep,

Beep, beep, beep, beep, beep, beep.

The horn on the bus goes beep, beep, beep,

All day long.

**23. Three Little Monkeys Jumping on the Bed**

Three little monkeys jumping on the bed.

One fell off and bumped his head.

Mummy called the doctor and the doctor said,

"No more monkeys jumping on the bed!"

Two little monkeys jumping on the bed.

One fell off and bumped his head.

Mummy called the doctor and the doctor said,

"No more monkeys jumping on the bed!"

One little monkey jumping on the bed.

He fell off and bumped his head.

Mummy called the doctor and the doctor said,

"No more monkeys jumping on the bed!"

**24. The Grand Old Duke of York**

Oh, the grand old Duke of York,

He had ten thousand men.

He marched them up to the top of the hill

And he marched them down again.

And when they were up, they were up.

And when they were down, they were down.

And when they were only halfway up

They were neither up nor down.

**25. Incy Wincy Spider (x2)**

Incy Wincy spider

climbed up the water spout

Down came the rain and

washed the spider out

Out came the sunshine

and dried up all the rain.

So Incy Wincy spider

climbed up the spout again.

**26. Jack and Jill (x3)**

Jack and Jill went up the hill

to fetch a pail of water

Jack fell down and broke his crown

and Jill came tumbling after.

**27. Humpty Dumpty (x3)**

Humpty Dumpty sat on the wall,

Humpty Dumpty had a great fall.

All the king's horses and all the King's men

Couldn't put Humpty together again.

**28. Ring a Ring a Roses (x3)**

Ring-a-ring o' roses,

A pocket full of posies.

A-tishoo, a-tishoo,

We all fall down.

**29. Row Your Boat**

Row, row, row your boat

Gently down the stream.

Merrily, merrily, merrily, merrily,

Life is but a dream.

Row, row, row your boat

Gently out to sea.

Merrily, merrily, merrily, merrily,

We'll be home for tea.

Row, row, row your boat

Gently on the tide.

Merrily, merrily, merrily, merrily

To the other side.

**30. Hickory Dickory Dock (x3)**

Hickory dickory dock,

The mouse ran up the clock.

The clock struck one,

The mouse ran down.

Hickory dickory dock.

**31. Little Miss Muffett (x3)**

Little Miss Muffett

Sat on a tuffett

Eating her curds and whey.

There came a big spider

who sat down beside her

and frightened Miss Muffett away.

**32. Little Jack Horner (x3)**

Little Jack Horner

sat in a corner

eating a Christmas pie.

He put in his thumb

and pulled out a plum

and said, what a good boy am I.

**33. Little Boy Blue (x2)**

Little boy blue

come blow your horn.

The sheep's in the meadow

the cow's in the corn.

Where is the boy who looks after the sheep?

He's under a haystack fast asleep.

Will you wake him?

No, not I.

For if I do

He's sure to cry.

**34. Here We Go Round the Mulberry Bush**

Here we go round the mulberry bush,

The mulberry bush, the mulberry bush.

Here we go round the mulberry bush

On a cold and frosty morning.

This is the way we wash our hands,

Wash our hands, wash our hands.

This is the way we wash our hands

On a cold and frosty morning.

This is the way we brush our hair,

Brush our hair, brush our hair.

This is the way we brush our hair

On a cold and frosty morning.

**35. Ride a Cock Horse (x2)**

Ride a cock horse to Banbury Cross

To see a fine lady upon a white horse.

With rings on her fingers and bells on her toes,

She shall have music wherever she goes.

**36. To market to market (x2)**

To market, to market, to buy a fat pig.

Home again, home again, dancing a jig.

To market, to market, to buy a fat hog.

Home again, home again, jiggety-jog.

To market, to market, to buy a plum bun.

Home again, home again, market is done.

**37. Two cats of Kilkenny (x2)**

There once were two cats of Kilkenny.

Each thought there was one cat too many.

So they fought and they fit

And they scratched and they bit,

Till excepting their nails

and the tips of their tails,

Instead of two cats there weren't any.

**38. Pussycat Pussycat (x2)**

Pussycat, pussycat, where have you been?

I've been up to London to visit the Queen.

Pussycat, pussycat, what did you there?

I frightened a little mouse under her chair.

**39. Ladybird Ladybird (x2)**

Ladybird ladybird fly away home.

Your house in on fire and your children are gone.

All except one and that's little Ann,

For she crept under the frying pan.

**40. There was an Old Lady**

There was an old lady who swallowed a fly.

I dunno why she swallowed that fly,

Perhaps she'll die.

There was an old lady who swallowed a spider,

That wiggled and wiggled and tickled inside her.

She swallowed the spider to catch the fly.

But I dunno why she swallowed that fly,

Perhaps she'll die.

**41. Oranges and Lemons**

"Oranges and lemons" say the Bells of St. Clement's.

"You owe me five farthings" say the Bells of St. Martin's.

"When will you pay me?" say the Bells of Old Bailey.

"When I grow rich" say the Bells of Shoreditch.

"When will that be?" say the Bells of Stepney.

"I do not know" say the Great Bells of Bow.

Here comes a candle to light you to bed.

Here comes a chopper to chop off your head!

**42. Curly Locks (x2)**

Curly locks, curly locks,

Will you be mine?

You shall not wash dishes

Nor feed the swine.

But sit on a cushion

And sew a fine seam,

And sup upon strawberries,

Sugar, and cream.

**43. Rock-a-Bye Baby (x2)**

Rock-a-bye baby,

On the treetop.

When the wind blows,

The cradle will rock.

When the bough breaks,

The cradle will fall,

and down will come baby,

Cradle and all.

**44. Lavender's Blue**

Lavender's blue, dilly, dilly,

Lavender's green.

When I am king, dilly, dilly,

You shall be queen.

Call up your men, dilly, dilly,

Set them to work.

Some to the plough, dilly, dilly,

Some to the cart.

Some to make hay, dilly, dilly,

Some to thresh corn.

While you and I, dilly, dilly,

Keep ourselves warm.
