# Supplementary material for: Acoustic-Emergent Phonology in the Amplitude Envelope of Child-Directed Speech
Source: PLoS One. 2015 Dec 7;10(12):e0144411. doi: 10.1371/journal.pone.0144411 (PMC4671555; doi:10.1371/journal.pone.0144411)

**Spectral RMS Power and Spectral Correlation Patterns**

*RMS Power.*

The RMS (root-mean-square) spectral power of the 29 cochlear channels was computed. Since the actual RMS power varied across samples and speakers, for each sample, the average power across all spectral channels was subtracted from each channel, leaving only the difference from the average. This difference was then averaged over samples and speakers. Figure a shows the computed difference RMS power by spectral channel, averaged over all 44 nursery rhymes and 6 speakers. As shown in the figure, RMS power is strongest for low spectral frequencies around 200 Hz, and steadily declines as frequency increases.

*Figure a. RMS power across 29 ERB_N_-spaced cochlear channels*


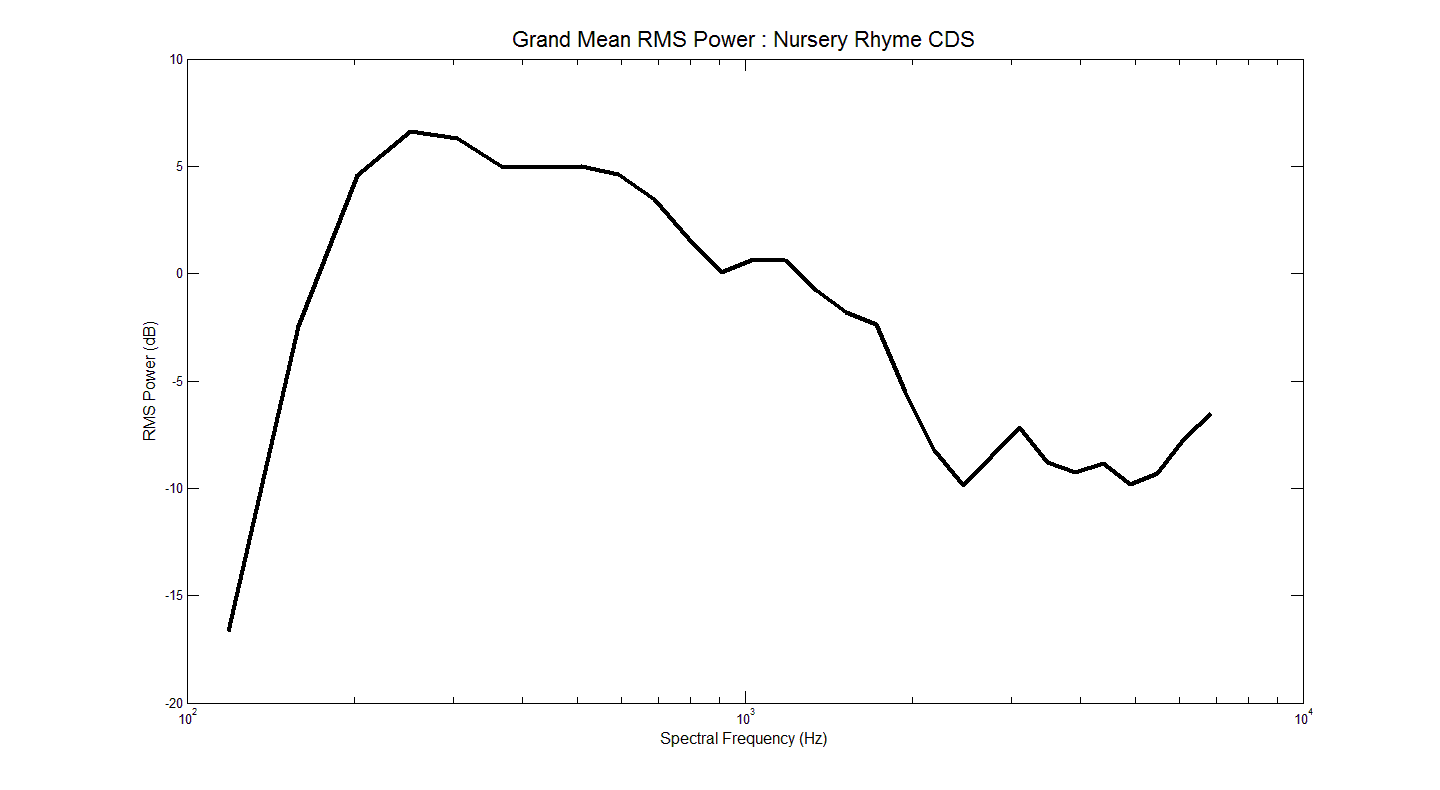


As expected from the classical acoustic phonetics and engineering literatures, the long term power spectrum has a low-frequency maximum with gradual fall towards high frequencies and Figure a confirms this for the present material. Power is generally stronger at lower spectral frequencies, and decreases approximately logarithmically (since the y-axis unit is dB) with increasing spectral frequency, thereby obeying a 1/f law. The drop off in power below ~200-300 Hz corresponds to the approximate lower end of the fundamental frequency for female speakers.

*Spectral Correlation.*

Next, the cross-(spectral)-channel correlation was computed for the 29 cochlear channels. Before this could be done, a thresholding procedure was employed to remove extraneous low-level modulations in the envelope arising from background noise. Such noise would reduce the true correlation between spectral channels. For each spectral channel, the long-term RMS power was determined. Using a threshold of -16dB from the long-term RMS power of each spectral channel, all portions of the envelope with power above this value were left unchanged. All time periods of the signal with power below RMS -16dB were set to RMS -16dB (henceforth referred to as the 'floor') plus a very small amount (amplitude of 1^-10^) of random noise. This small amount of noise was added to portions that did not meet the threshold level so that floored sections of the envelope would not be completely flat, as this could artificially elevate correlations between spectral channels in subsequent analyses. This flooring procedure is described in greater detail in Stone & Moore (2007). Figure b shows an example of the original (black) and floored (red, dashed) envelopes for two adjacent spectral (cochlear) channels. Since the human discrimination of intensity and the subjective sensation of loudness varies approximately logarithmically with signal power (Fechner, 1860), the base 10 logarithm of the floored envelope in each band was taken, and this logarithmic envelope was used for the subsequent correlation analysis.

*Figure b. Example of floored envelopes in adjacent spectral channels*


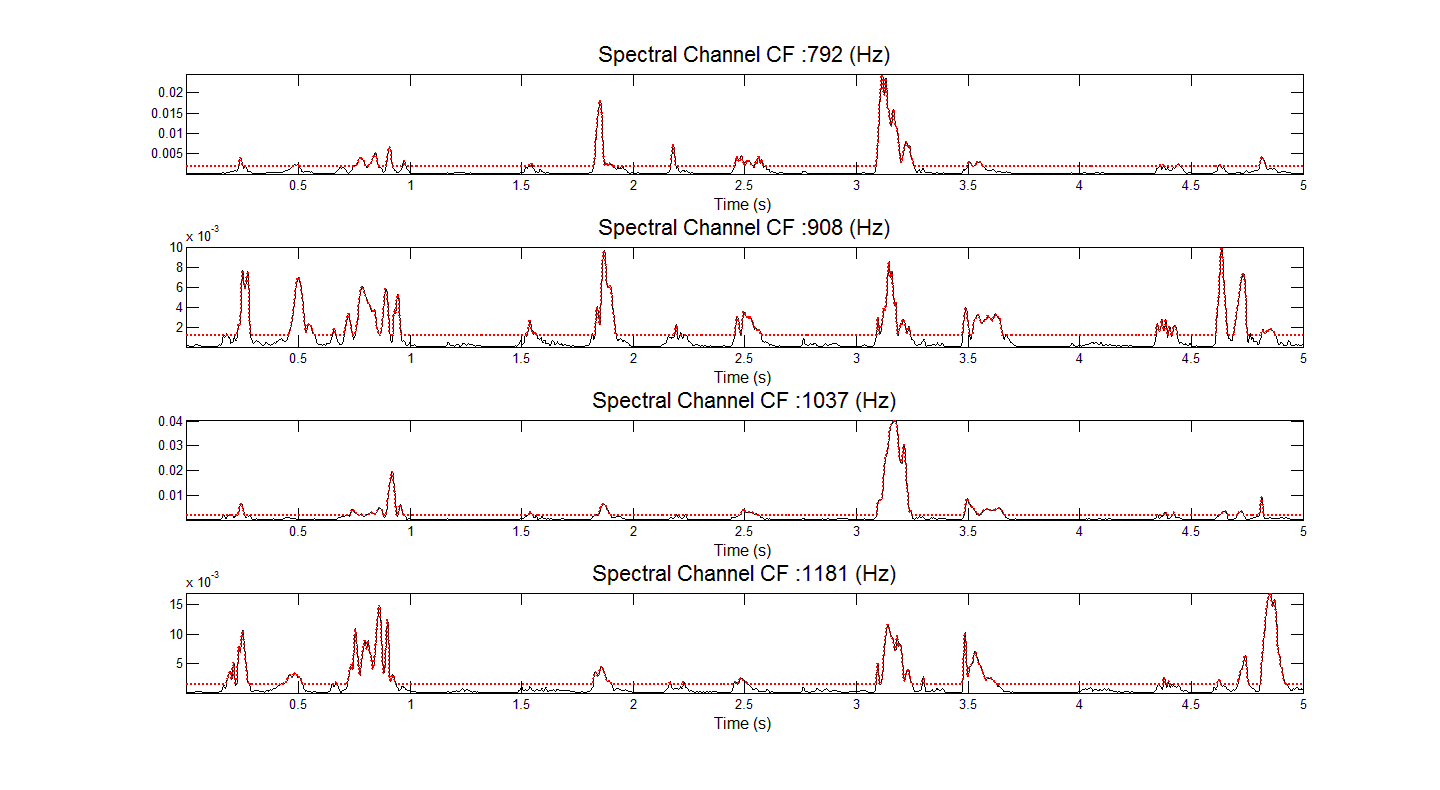


For the correlation analysis, only the unfloored sections of each cochlear channel (i.e. active sections) were correlated with the temporally-corresponding sections of all the other cochlear channels. To do this, floored sections were excised from the target channel, and the remaining unfloored sections were concatenated. This concatenated envelope was then z-scored, and correlated with temporally-corresponding z-scored sections of every other channel (ie irrespective of whether those were floored or unfloored). Thus the temporally-corresponding sections in other channels could contain floored (silenced) sections as well as unfloored (active) sections, although the target channel itself only contained unfloored (active) sections.

Figure c shows the result of this cross-correlation across cochlear channels (with zero lag), where the mean correlation coefficient over 44 speech samples and 6 speakers is plotted. Visual inspection of the figure shows that mid-frequency spectral channels around 1000 Hz (green-cyan) show the strongest correlation with each other in this general region, and with other spectral channels as well. There is also some evidence of channel 'clustering' for example among green-cyan (~1000 Hz) channels, or among blue (~3000 Hz) channels.

*Figure c. Intercorrelations between spectral channels. In the top plot, each coloured line indicates a single cochlear channel.*


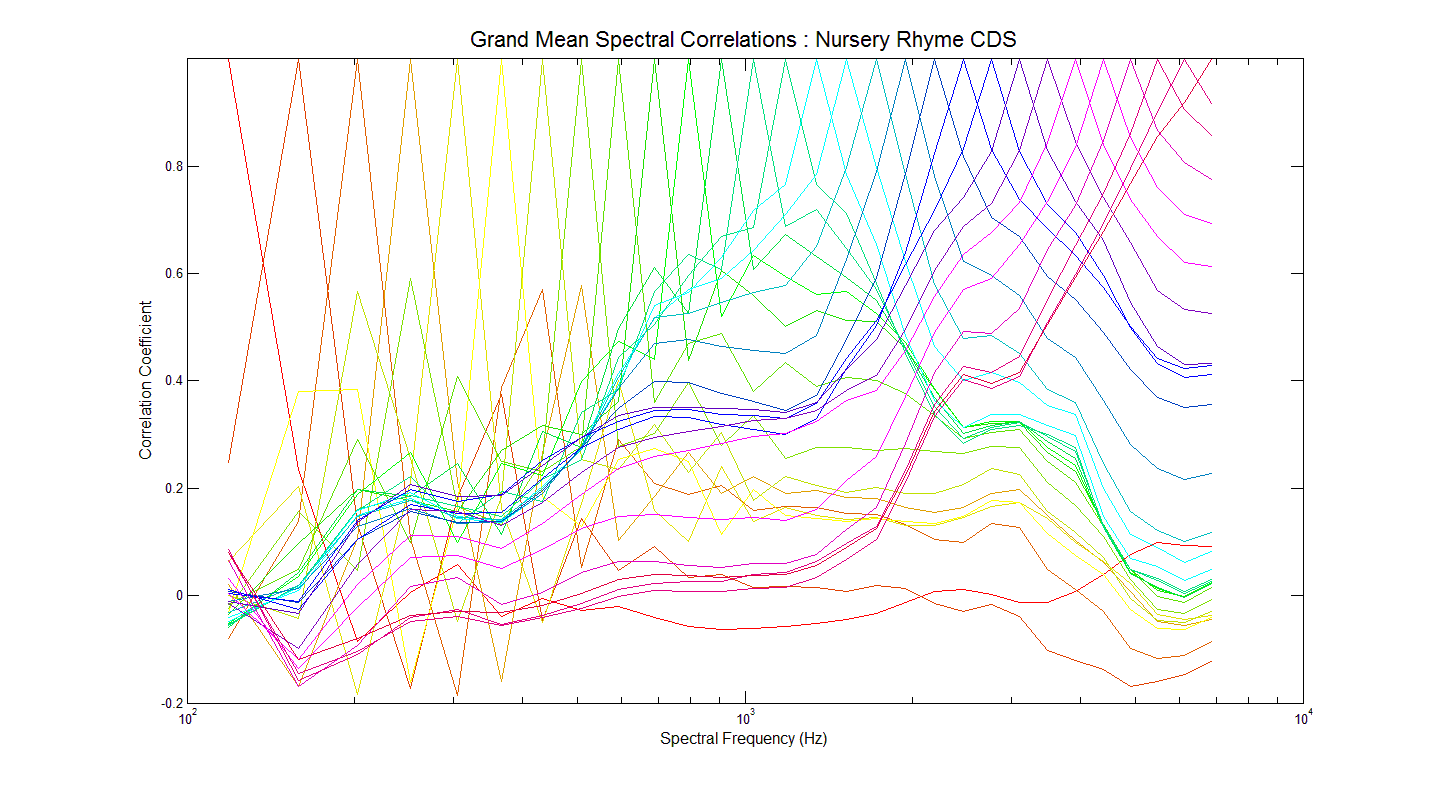


*Spectral Correlations by Spectral Band*

5 spectral bands were identified on the basis of the rectified component loading pattern of the top 5 principle components (see Results section). The top panel of Figure d shows the mean correlation of the cochlear channels in each spectral band (shown as coloured lines) with the channels in other spectral bands. The black dotted line shows the grand mean correlation over all cochlear channels. Note the similarity in pattern between this grand mean correlation, and the loading pattern of principle component 1 in the spectral PCA. The bottom panel of Figure d shows the RMS power across the 29 cochlear channels, with the boundaries of the spectral bands superimposed as vertical dotted lines. It is interesting to note that there is an approximately inverse relationship between RMS power and correlation strength. So Spectral Band 4, which has lowest power, shows strong correlations with adjacent channels (has the highest mean). In contrast, Spectral bands 1 & 2 have high power, but only correlate weakly with other channels. Hence, the RMS power in an audio frequency region is not necessarily a good indicator of its correlation strength with other channels.

*Figure d. (top) Mean spectral correlations between each spectral band and the other spectral bands. Each band is shown in a different colour. The black dotted line indicates the grand mean correlation over all spectral bands. The vertical dotted line shows the boundary between spectral bands. (bottom). RMS power of each cochlear channel, with spectral band boundaries overlaid as vertical dotted lines.*

*
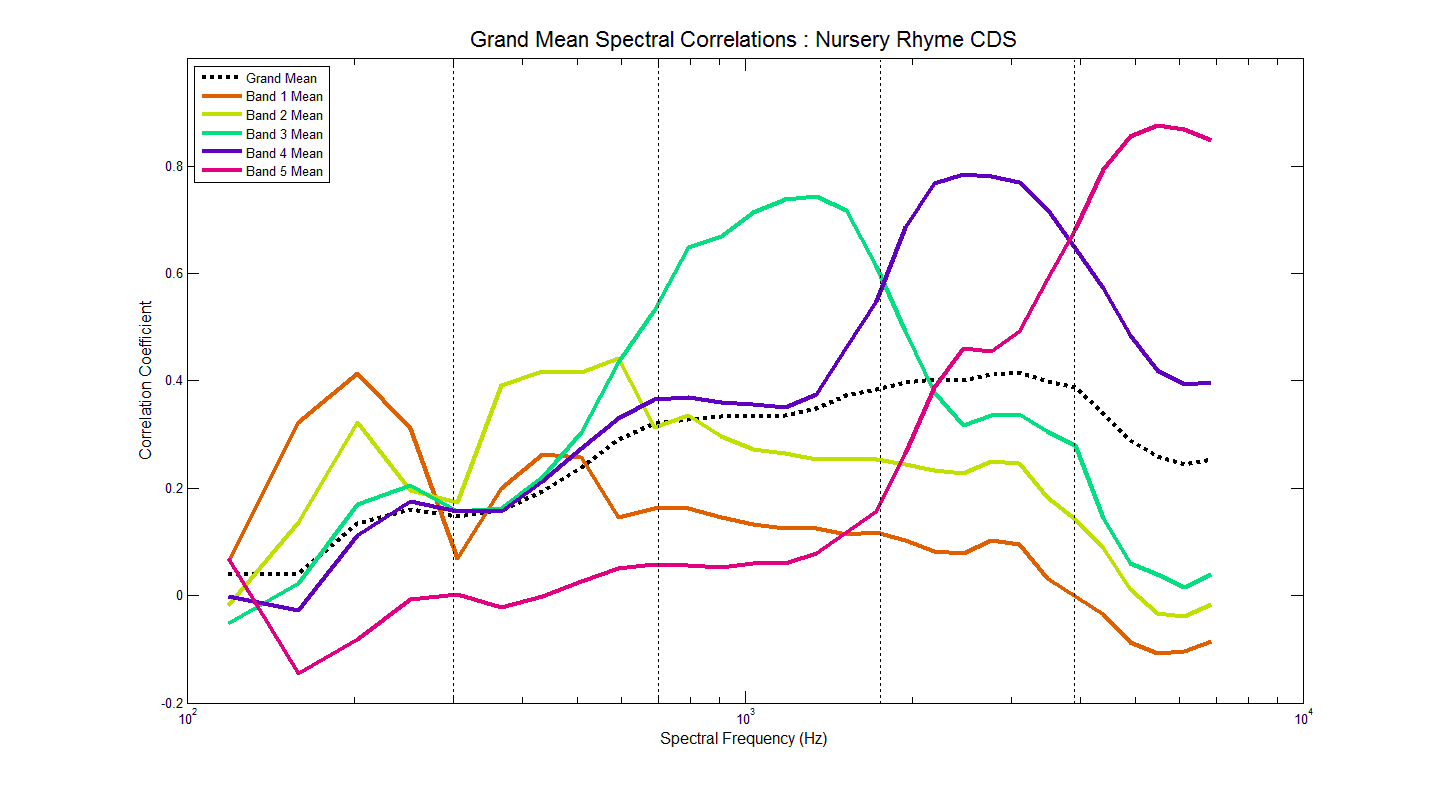
*


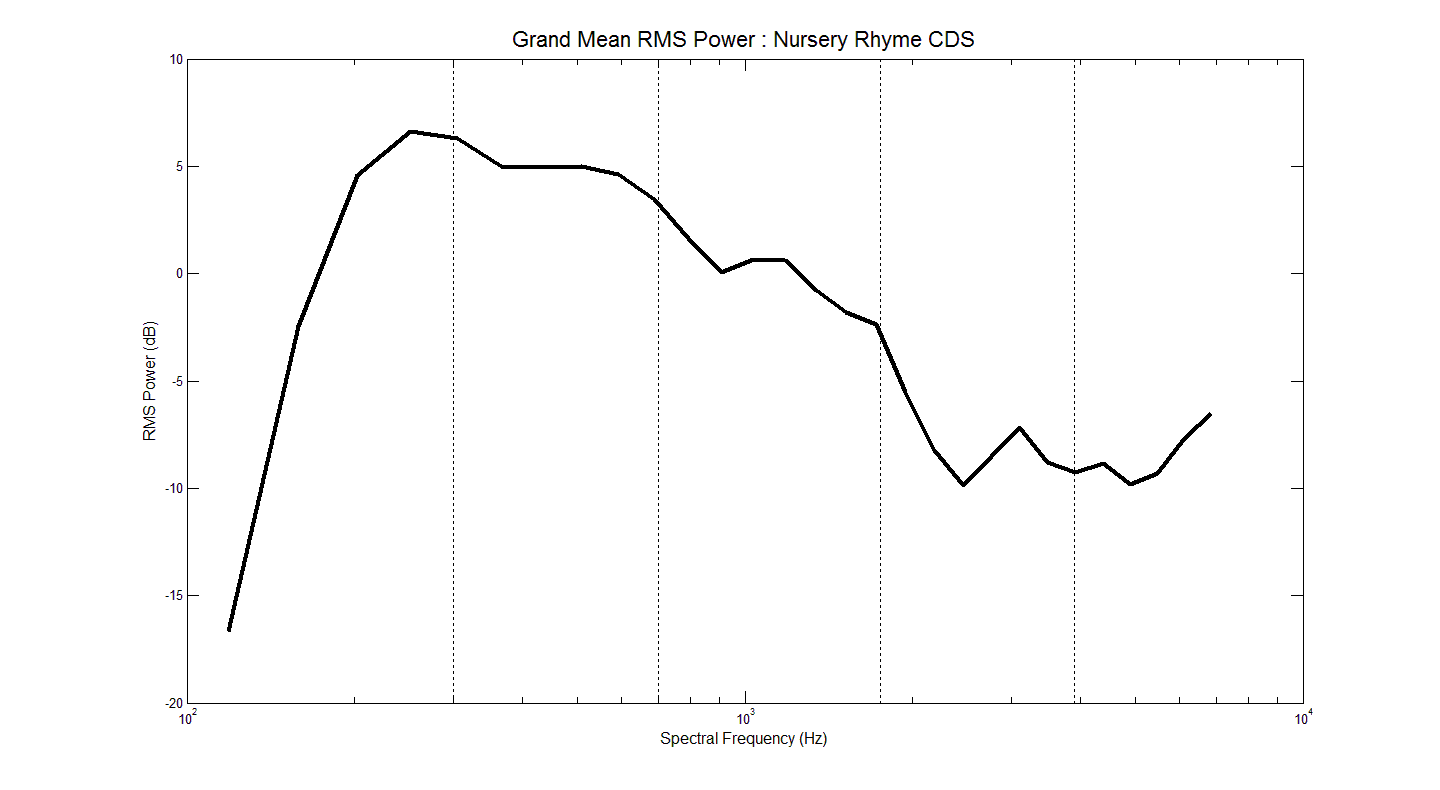

Supplement: S2 Appendix — (DOCX) [file pone.0144411.s002.docx]
