# Supplementary material for: Acoustic-Emergent Phonology in the Amplitude Envelope of Child-Directed Speech
Source: PLoS One. 2015 Dec 7;10(12):e0144411. doi: 10.1371/journal.pone.0144411 (PMC4671555; doi:10.1371/journal.pone.0144411)
Supplement: S3 Appendix — (DOCX) [file pone.0144411.s003.docx]

**Individual Variation in Spectral PCA Component Loadings**

Figure a shows the spectral PCA component loading patterns for each individual speaker (P01 to P06). In each subplot, the lines of different thickness indicate different PCA components. More important (lower numbered) components are shown in a thicker line. The loading patterns for the top 5 PCA components shown here are broadly similar across the 6 speakers. The total amount of variance explained by the top 5 components was also similar across speakers, ranging from 62.1% to 68.0%. It may be observed that the 6 speakers produced broadly consistent PCA loading patterns, particularly for the first 3 components.
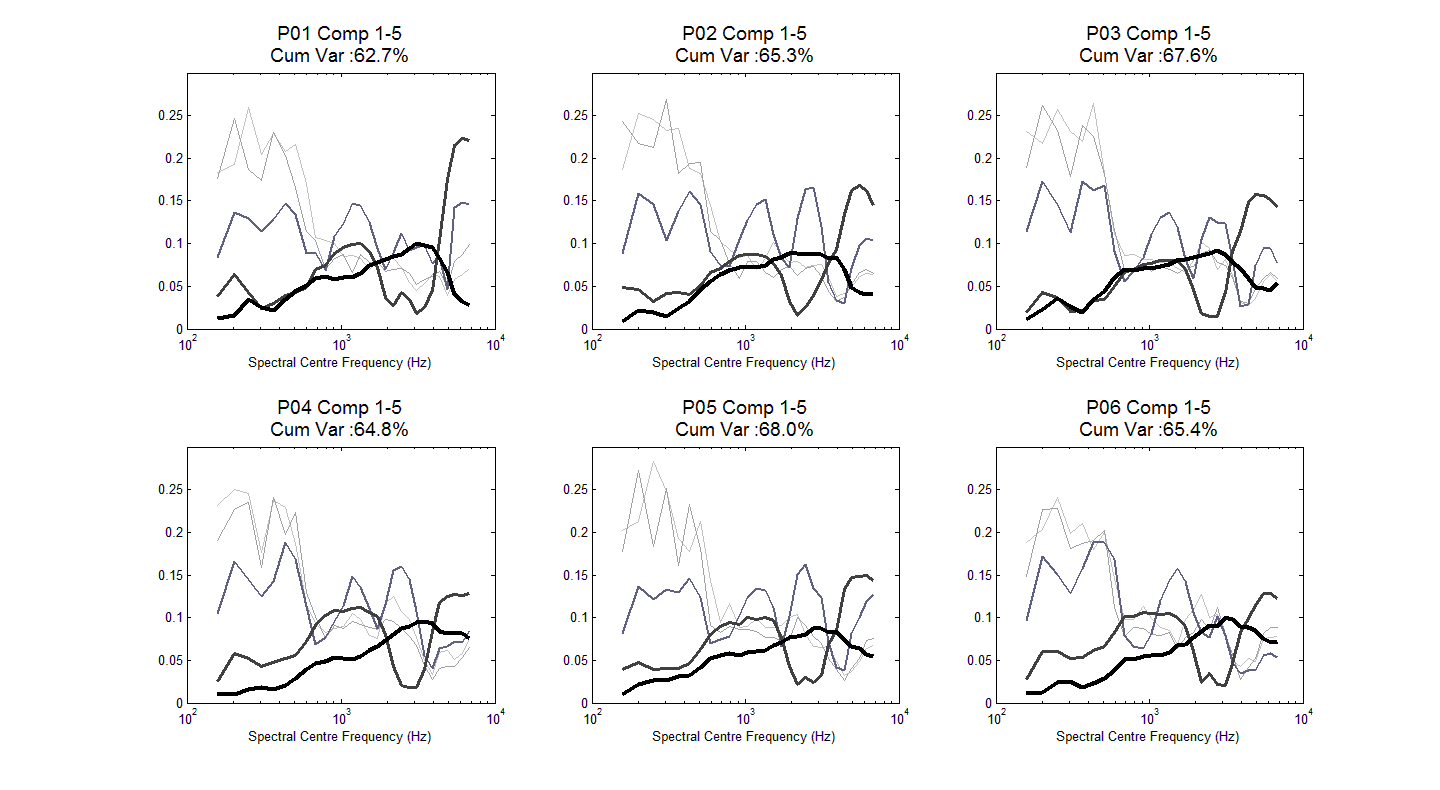


*Figure a. Individual spectral PCA component loading patterns*

The population distribution of peaks and troughs across the 6 speakers for each principal component is shown in Figure b. In Figure b, the x-axis indicates the spectral centre frequency, the y-axis indicates the frequency with which a peak or trough was identified at that spectral frequency, and the different coloured bars indicate the first 5 principle components. The population distribution of trough locations (top panel of Figure b) indicates that, across the 5 PCs, most of our speakers showed troughs at ~300 Hz, between 700-800 Hz, at ~1700 Hz and between 3000-4400 Hz. These spectral regions are broadly consistent with the 4 band divisions identified on the basis of the grand mean PCA loading patterns in the main text. However, this figure also shows that there is considerable individual variation in trough locations, particularly for the middle spectral frequencies. Nonetheless, there is a very high population tendency for a trough to be located at the low frequency of ~300 Hz, delineating the boundary between spectral bands 1 & 2. The population distribution of peaks provides a complementary picture. Most speakers show a peak within Band 1 (~200 Hz), and there is also a weaker population tendency towards peaks in PC loading at ~400 Hz (Band 2), between 900-1300 Hz (Band 3), at ~2500 Hz (Band 4) and at the ~6000 Hz (Band 5). In conclusion, the Spectral Band divisions appear to highly consistent across individuals at low spectral frequencies (i.e. Bands 1 & 2), and less consistent across individuals for mid-to-high frequencies.


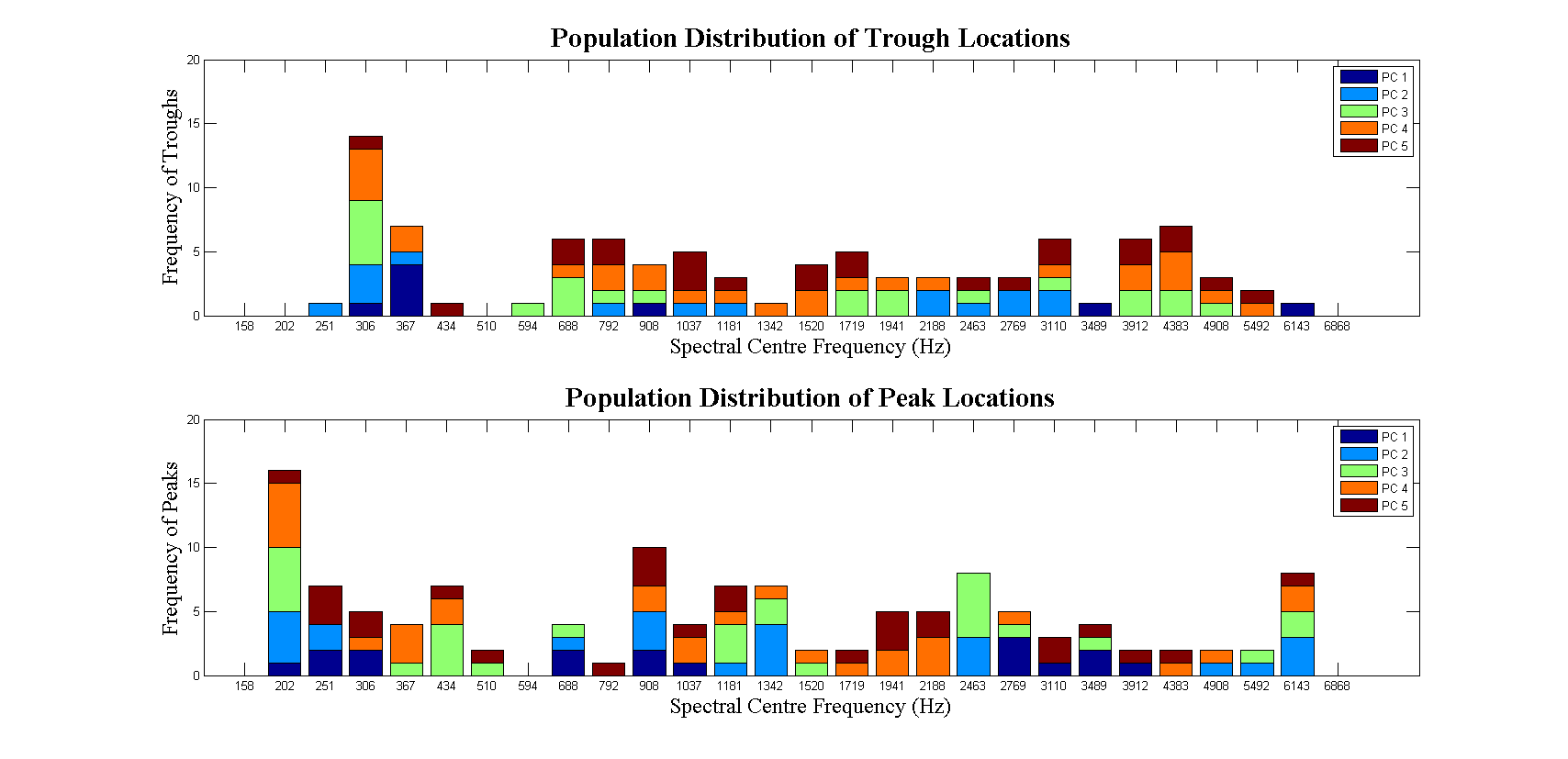
**Individual Variation in Modulation Rate PCA Component Loadings**

*Figure b. Population distribution of trough locations (top) and peak locations (bottom)*

Figure c below shows the modulation rate PCA component loading patterns for each individual speaker (P01 to P06). In each subplot, the lines of different thickness indicate different PCA components. More important (lower numbered) components are shown in a thicker line, and the components from the 5 spectral bands are plotted in different colours. It may be observed that all 6 speakers showed broadly similar patterns of loading for PCA components 1, 2 and 3.

*Figure c. Individual modulation rate PCA component loading patterns*


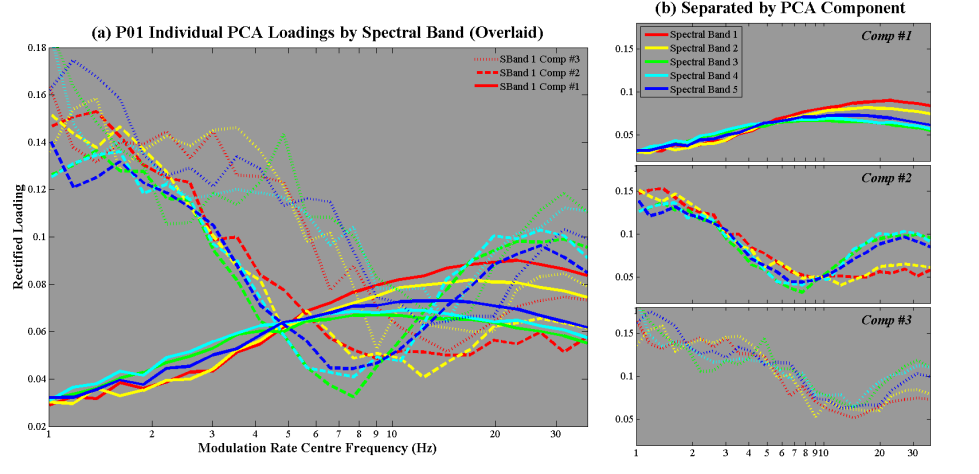

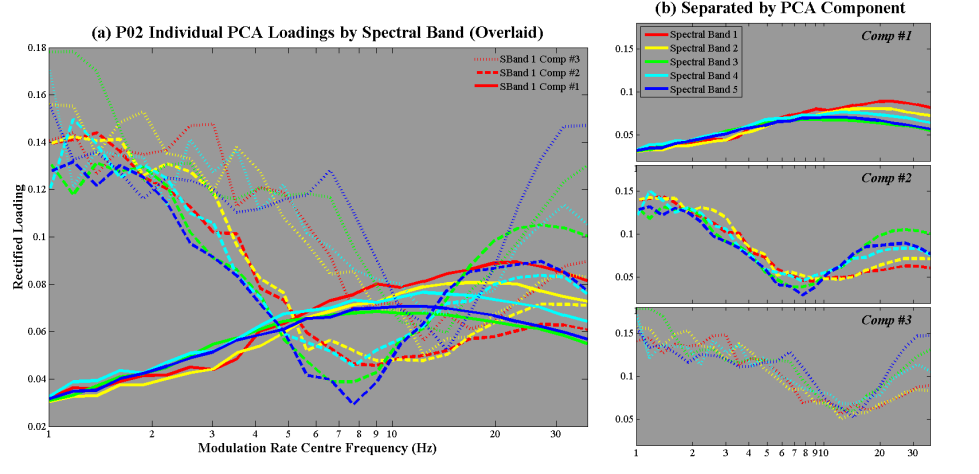

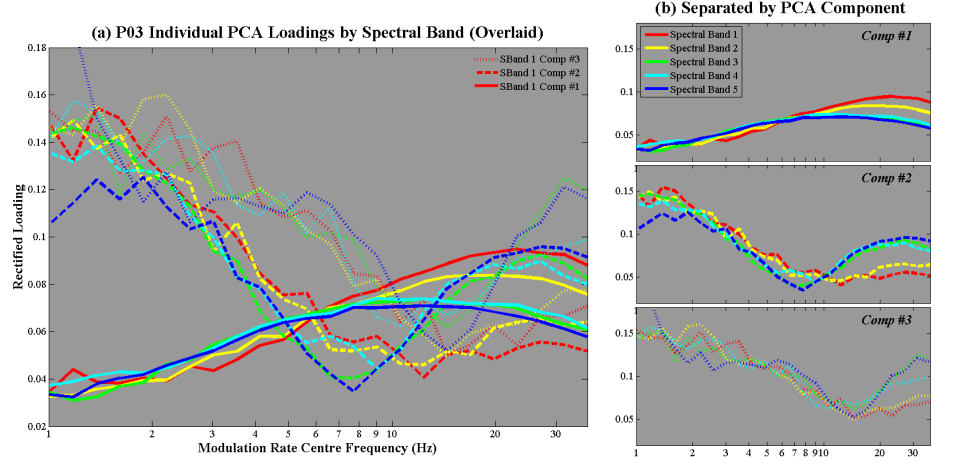


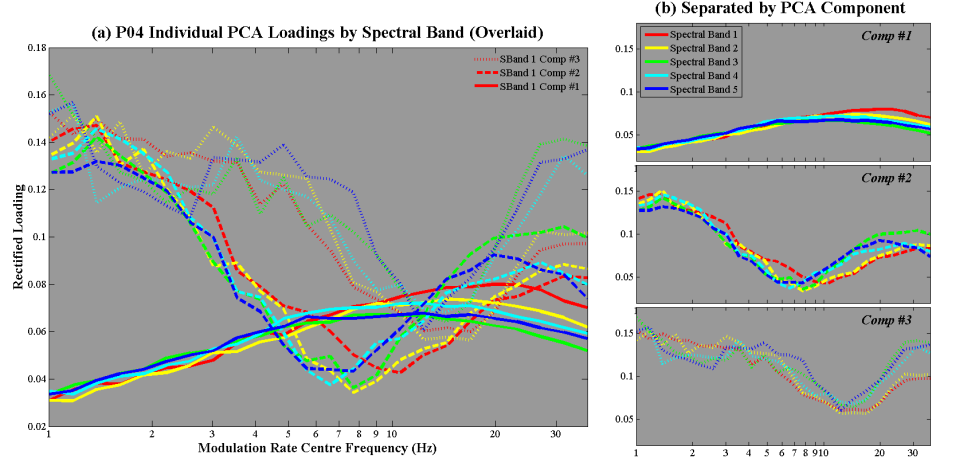

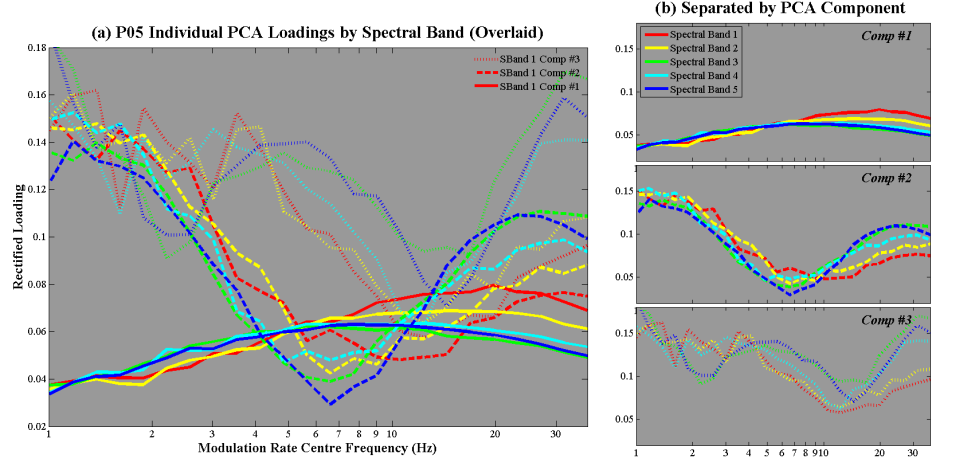

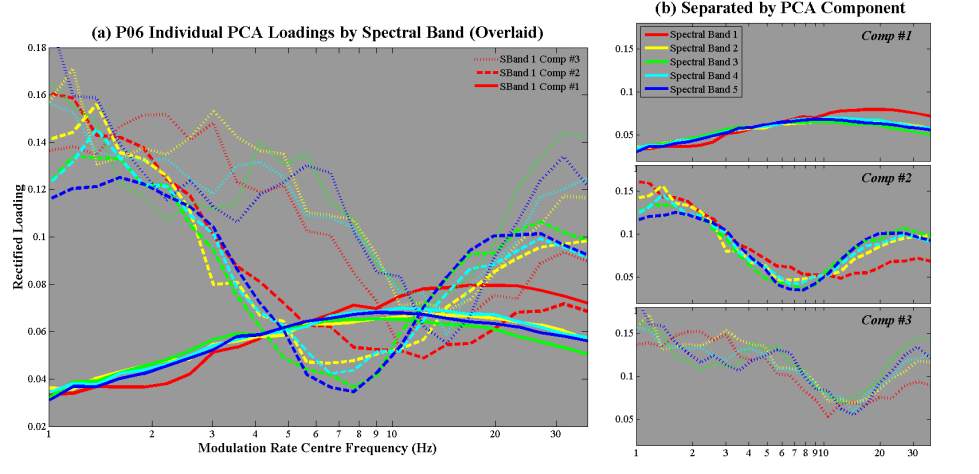


The population distribution of peaks and troughs across the 6 speakers for each principal component is shown in Figure d. For each speaker's subplot, the x-axis indicates the modulation rate centre frequency, the y-axis indicates the frequency with which a peak or trough was identified at that rate, and the different coloured bars indicate the first 3 principle components. The population distribution of peaks (bottom panel) indicated that there was a clear clustering of peaks in 3 regions (~1.4 Hz, between 3.5 Hz-6 Hz, and between 27-31 Hz) which corresponded well to the existence of 3 AM bands (Stress, Syllable and Phoneme) in these respective regions. Correspondingly, the population distribution of trough locations (top panel of Figure d) indicates there was a highly consistent appearance of a trough at ~12-14 Hz, carried entirely by PC3, and corresponding to the division between Syllable AM and Phoneme/Onset-Rime AM rate bands in the S-AMPH model. In the low frequency region, there was also a strong tendency for troughs to appear within a fairly broad range of 1.2-3 Hz (particularly carried by PCs 1 & 2) suggesting the existence of the Stress AM-Syllable AM band division in this region. However, this Stress AM-Syllable AM band division was less well-defined and more variable across speakers than the Syllable AM-Phoneme AM division.


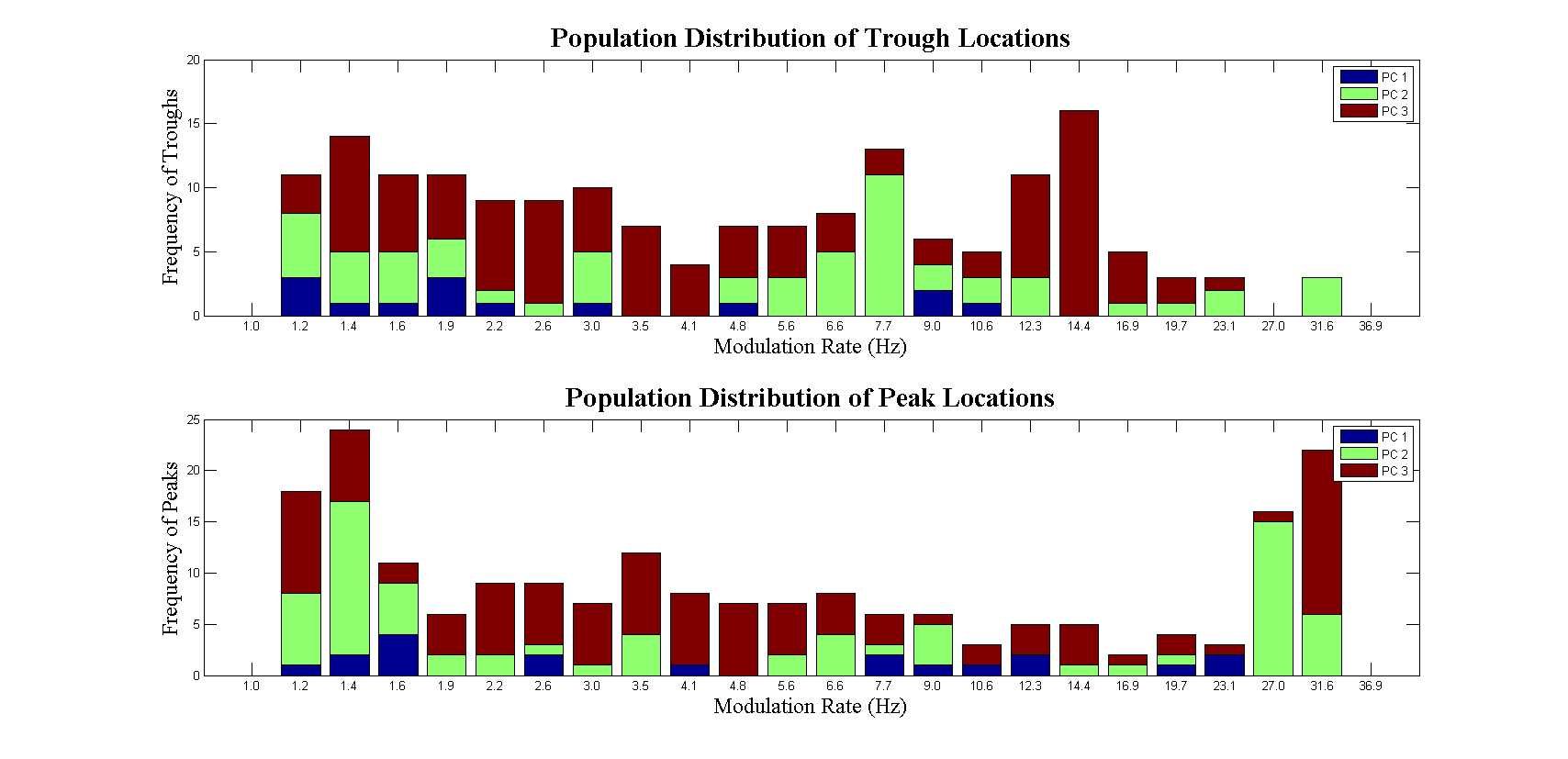


*Figure d. Population distribution of trough locations (top) and peak locations (bottom) for modulation rate PCA*
