# Supplementary material for: Acoustic-Emergent Phonology in the Amplitude Envelope of Child-Directed Speech
Source: PLoS One. 2015 Dec 7;10(12):e0144411. doi: 10.1371/journal.pone.0144411 (PMC4671555; doi:10.1371/journal.pone.0144411)
Supplement: S4 Appendix — (DOCX) [file pone.0144411.s004.docx]

**Spectral and Temporal PCA with Other Speech Corpora**

*Acoustic Differences with Child-Directed and Adult-Directed Speech*

Child-directed speech (CDS) and adult-directed speech (ADS) refer to two different speaking 'registers' or styles. These differences are thought to arise because the speaker is adapting his or her speaking style to the language abilities and needs of his or her audience. In child-directed speech, these adaptations reflect the fact that the child is a novice language-learner, rather than an expert. Much of this adaptation occurs at the lexical and syntactic levels. For example, child-directed speech contains simpler syntactic structures (Sachs et al, 1976), shorter sentences (Barnes et al, 1983), and pertains to topics that are of interest to the child (Ferguson, 1977; Ferguson & Debose, 1977). However, adaptation also occurs at the perceptual-acoustic level. Child-directed speech is *prosodically-enhanced*, making it more interesting and engaging for the listener, and conveying a positive affect (Fernald, 1989). The perceptual-acoustic properties of CDS have commonly been studied in terms of pitch, duration, speaking rate, pauses, etc (Broen, 1972; Fernald & Simon, 1984; Fernald, 1989; Albin & Echols, 1996). Accordingly, it is possible that CDS and ADS also differ in their basic or core spectro-temporal modulation structure. Accordingly, the same PCA procedures were applied to 3 additional corpora of CDS and ADS elicited from the same 6 adult speakers, and these results were compared against those obtained from the main corpora of nursery rhymes (CDS) reported in the paper.

*Speech Materials*

Each speaker produced four speech corpora, two spoken in CDS and two spoken in ADS. These were (1) Nursery rhymes produced in child-directed speech (CDS Rhyme, corpus used in main paper); (2) Nursery rhymes produced in adult-directed speech (ADS Rhyme); (3) Children's stories produced in child-directed speech (CDS Story); and (4) Spontaneous conversation spoken in an adult-directed manner (ADS Conversation).

(1) CDS Rhyme & (2) ADS Rhyme

In these two corpora, the spoken material (nursery rhymes) was held constant, and speakers were told to change their manner of speaking as if addressing a child (CDS Rhyme) or an adult (ADS Rhyme). A total of 44 familiar children's nursery rhymes were used (see Appendix 1), and each speaker produced all 44 nursery rhymes first in ADS, then in CDS. Since the spoken material was identical, any differences between the two corpora should cleanly reflect perceptual-acoustic variations in speaking style, without being affected by differences in words or syntax. For the CDS version of the rhymes, participants spoke in a lively and rhythmic fashion, often speaking to the rhythm of tunes that were associated with these nursery rhyme (although the rhymes were not actually sung). For the ADS version of the rhymes, participants' utterances were less metrically-regular, and they attempted to produce the sentences with a 'normal' prosodic pattern, as appropriate to an adult audience.

(3) CDS Story

For the third speech corpus, participants read 5 classic children's stories in a child-directed manner. These stories were taken from the children's book 'The Puffin Baby and Toddler Treasury' (Puffin Books, 1998). The titles of the stories were 'The Gingerbread Man', 'The Three Billy Goats Gruff', 'Goldilocks and the Three Bears', 'The Three Little Pigs' and 'The Ugly Duckling'. The language used in these stories was simple, and appropriate for nursery and preschool aged children. Depending on the reading speed of the speaker, the recordings of the stories ranged from 4 to 8 minutes in length for each story. To produce speech samples that were comparable in length and quantity to the 44 nursery rhymes, nine continuous sections were extracted from each of the five stories, giving 45 story sections for each speaker. For each speaker, the length of each story segment was the same as the mean length across their 88 CDS + ADS nursery rhyme samples. These mean lengths for each speaker are shown in Table a. Different segment lengths were used for each individual (rather than one standard length for the whole group) because the speakers differed substantially in speaking rate. Therefore, to ensure that the same quantity of spoken material (i.e. syllables and words) was captured for each individual across nursery rhymes and stories, individually-adjusted segment lengths were used. Therefore, if an individual's speaking rate was slower, this would result in a longer mean section length for the nursery rhymes. This longer length would then also be used to segment her read stories so that each story sample would contain on average the same quantity of syllables and words as each nursery rhyme sample.

*Table a. Mean lengths for CDS and ADS nursery rhyme samples by speaker, used to determine section length for CDS Story samples.*

| **Speaker** | **Mean Section Length (s)** |
| --- | --- |
| 1 | 25.6 |
| 2 | 23.9 |
| 3 | 27.5 |
| 4 | 34.3 |
| 5 | 24.5 |
| 6 | 26.1 |

(4) ADS Conversation

For the fourth speech corpus, spontaneously-produced adult-directed speech was recorded. Here, participants were provided with suggested topics to speak about, and were given a few minutes for mental preparation before they began speaking. These suggested topics were :

• Describe a typical day at work (or home)?

• Describe a book that you've read?

• Describe a film that you've watched?

• What leisure activities and hobbies do you enjoy?

• What are you looking forward to in the summer?

Participants were told to speak about each topic for about 2-3 minutes, before moving on to the next topic. Since no verbal feedback was given and participants spoke continuously, these samples essentially comprised a narrative monologue that lasted around 10-12 minutes. As all the participants were eloquent speakers, the sentences in these conversation samples were typically well-formed and grammatically correct. Similar to the CDS Story recordings, sections were extracted from the continuous ADS recording for analysis. These sections were matched in length to the nursery rhymes produced by each speaker. Since the total amount of spontaneous conversation varied from speaker to speaker, the total number of segmented samples also differed from speaker to speaker, ranging from 23 to 34 samples, with a mean of around 27 samples.

Table b summarises the speaking style and material used for the four speech corpora. In total, each of the 6 speakers contributed 44 CDS Rhyme samples, 44 ADS Rhyme samples, 45 CDS Story samples and ~27 ADS Conversation samples. This gave a total of ~160 samples per speaker (each around 25s-35s in length), and a grand total of ~960 samples across all the speakers.

*Table b. Features of the four speech corpora*

|  | **Speaking Style** | **Type of Material** |
| --- | --- | --- |
| (1) CDS Rhyme | Child-directed | Nursery Rhymes |
| (2) ADS Rhyme | Adult-directed | Nursery Rhymes |
| (3) CDS Story | Child-directed | Children's Stories |
| (4) ADS Conversation | Adult-directed | Free Conversation |

*Spectral & Modulation Rate PCA Procedures*

Prior to performing the PCA analysis on the 28 cochlear-spaced frequency channels, the RMS power for the spectral channels was computed. This initial step was performed to see if there were any obvious differences in patterns of spectral power across the 4 speaking conditions, before the more complex PCA analysis was applied. A Spectral PCA procedure was then applied to the samples, as described in the Methods section. For this PCA analysis, the speech signal was divided into 29 ERB_N_-spaced frequency channels, and the Hilbert envelope was taken from each spectral channel. The first low-pass channel was discarded, and the remaining 28 spectral envelopes were then entered into a PCA analysis as separate variables. As before, the absolute value of the spectral channel loadings was taken, and these rectified values were averaged across all the samples, for each speaking condition. The mean rectified PCA loading patterns for each speaking condition were then compared.

For the modulation rate PCA, the speech signal was first divided into 5 spectral bands (as indicated by the results of the previous Spectral PCA). The Hilbert envelope was obtained for each spectral band and then low-pass filtered under 40 Hz. These 5 Hilbert envelopes from each spectral band were then individually passed through a 24-channel logarithmically-spaced modulation filterbank spanning 0.9-40 Hz. Prior to conducting the PCA, the RMS power of the 24 modulation channels was computed to look for obvious differences in the modulation spectrum. Then, the modulation rate PCA procedure was applied as deswcribed in the Methods section. This PCA analysis was repeated separately for each of the 5 spectral bands. The absolute value of the modulation rate channel loadings was again taken, and these rectified values were averaged across all the samples for each speaking condition. The mean rectified PCA loading patterns for each speaking condition were then compared.

*Results*

RMS Power Across Spectral Channels

Figure a shows the grand mean RMS power over the 28 cochlear channels for each speaking condition. The 4 speech corpora are each plotted in a different colour. From visual inspection, there appear to be clear differences in RMS power between CDS and ADS samples within spectral bands 1, 3 and 5. In the middle spectral band 3 (700 Hz-1750 Hz), the two CDS conditions (yellow and red lines) clearly showed higher RMS power than the two ADS conditions (blue and green lines). The opposite was true for the two extreme spectral bands 1 (100 Hz -300 Hz) & 5 (3900 Hz to 7250 Hz), where the two ADS conditions now showed higher RMS power than the two CDS conditions. Moreover, the order of effects across speaking conditions was maintained even though the direction of differences was reversed.

*
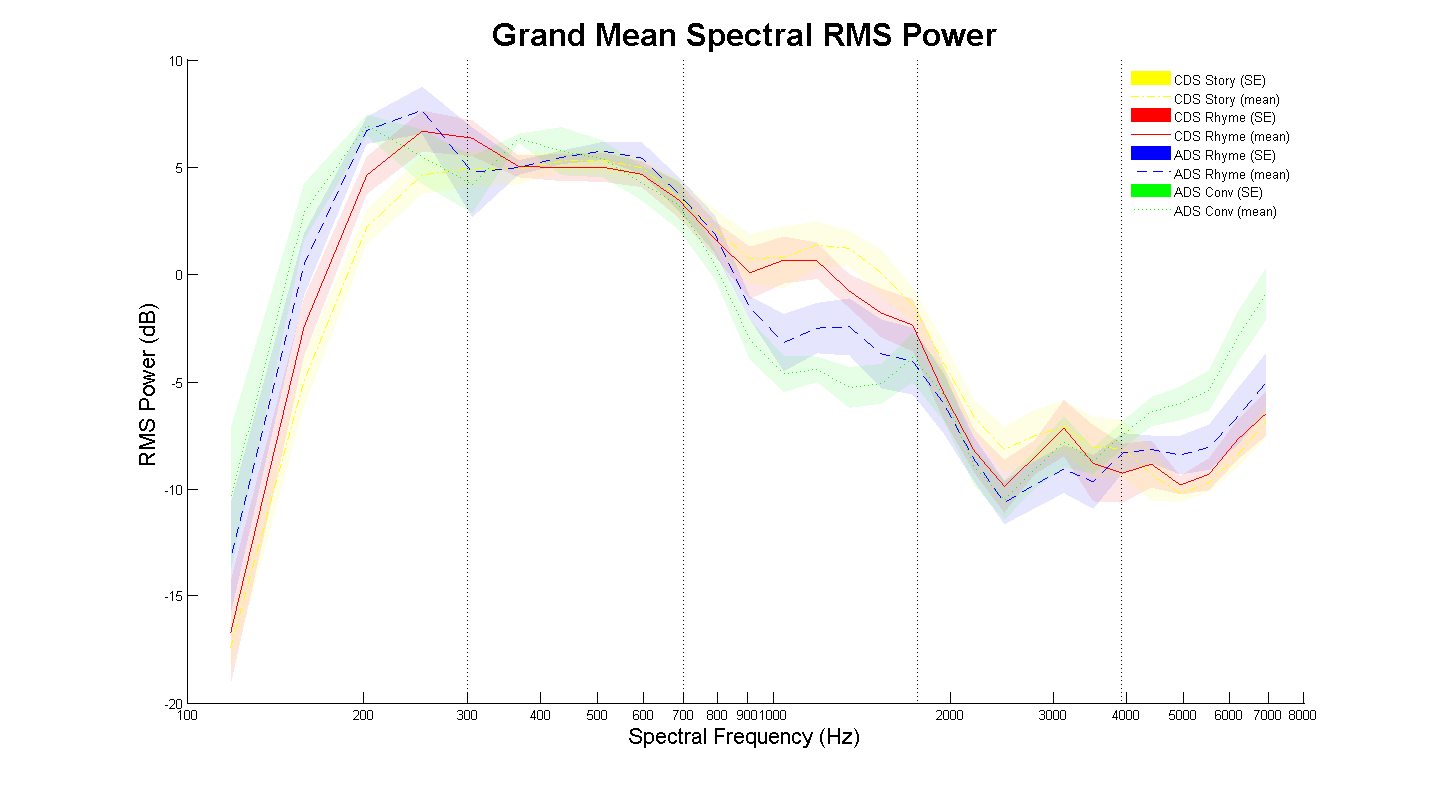
Figure a. RMS power of the 28 cochlear channels. Vertical dotted lines indicate the boundaries between the 5 S-AMPH spectral bands. The 4 speaking conditions are shown as different coloured lines. Shaded areas indicate the standard error of the mean.*

**Band 1 Band 2 Band 3 Band 4 Band 5**

In spectral band 3, CDS Story showed the highest RMS power, followed by CDS Rhyme, ADS Rhyme and ADS Conversation. In spectral bands 1 & 5, this order was perfectly reversed, with ADS Conversation now showing the highest RMS power, followed by ADS Rhyme, CDS Rhyme and CDS Story. This orderly pattern of RMS power differences suggests that child- and adult-directed speech differ systematically in their relative spectral composition. For child-directed speech, the power of the middle spectral frequencies was increased relative to the power of very low and very high spectral frequencies. For adult-directed speech, the middle spectral frequencies received less emphasis, relative to very low and very high spectral frequencies.

It is worth noting that the differences between CDS and ADS samples occurred in spectral regions that corresponded fairly well to the S-AMPH spectral band divisions. For example, the increase in power for CDS samples at the middle spectral frequencies occurred between ~800-1750 Hz, which correspond closely with the spectral band 3 region of 700-1750 Hz. This suggests that the 5 (PCA-derived) spectral bands do indeed reflect separate spectral components in speech, since they can be modulated independently (in power) by the speaker. However, recall from Appendix 4.3 that RMS power does not necessarily reflect correlation strength. Therefore, to investigate the underlying spectral correlation structure of the 4 types of speech more closely, a Spectral PCA analysis was conducted.

Spectral PCA

**a. Principal Component Loadings**

The loading patterns for the top 3 PCA components in each speaking condition are shown in Figure b. Only the top 3 components were analysed because cumulatively, these 3 components already accounted for over 50% of the total variance in the samples. The variance accounted for by each principal component is indicated in the titles of Figure b. In Figure b, the x-axis plots the centre frequency for each spectral channel and the y-axis plots the absolute (rectified) component loading, averaged across all samples and speakers. Each of the 4 speaking conditions is shown in a different colour. From visual inspection, the overall loading patterns were very similar across the four spoken conditions. The locations of major peaks and troughs (indicating spectral band boundaries) were also very similar across the four conditions, coinciding well with the band boundaries previously identified in the S-AMPH model. These previously-identified band boundaries are shown as vertical dotted lines. This suggests that the fundamental band structure of the speech samples was similar *across* the four speaking conditions, and agreed well with the spectral band boundaries that had previously been identified.

However, there were also some systematic differences between the four conditions. Although the spectral *location* of the peaks and troughs was similar across conditions, the relative *height* of the peaks differed systematically across conditions. For example, in PCA component 2 (middle plot of Figure b), loadings within spectral bands 3 and 5 (indicated by the white arrows) were clearly different across speaking conditions. In both these spectral regions, component loadings were, from highest to lowest : CDS Story > CDS Rhyme > ADS Rhyme > ADS Conversation. This same pattern of component loading was also observed in PCA component 3 (right plot) in the region of spectral band 5 (white arrow). Therefore, CDS samples loaded more strongly than ADS samples in mid-high frequency regions. The opposite pattern was observed for PCA component 1 in the regions of spectral band 3 and spectral band 1 (grey arrows). Here, the order of component loadings, from highest to lowest were : ADS Conversation/ADS Rhyme > CDS Story/CDS Rhyme. Therefore, ADS samples loaded more strongly than CDS samples in low-mid frequency regions. Note that in spectral band 3 (the middle frequency band), there was an opposite loading order across conditions for PCA components 1 & 2. In component 1, ADS samples loaded more strongly, but in component 2, CDS samples loaded more strongly. To estimate how these loading order differences would trade-off on average, the loadings for all the conditions were averaged across PCA components 1 to 3. The results of this averaging are shown in Figure c.

*Figure b. Spectral PCA rectified mean loading patterns for PCA components 1, 2 and 3 (shown from left to right). The four speaking conditions are shown in different coloured lines. The amount of variance accounted for by each PCA component, for each speaking condition is shown in the title of each plot (averaged across speakers). Vertical dotted grey lines indicate the boundaries between the five spectral bands. White arrows indicate spectral regions where CDS samples load more strongly than ADS samples. Grey arrows indicate spectral regions where ADS samples load more strongly than CDS samples.*


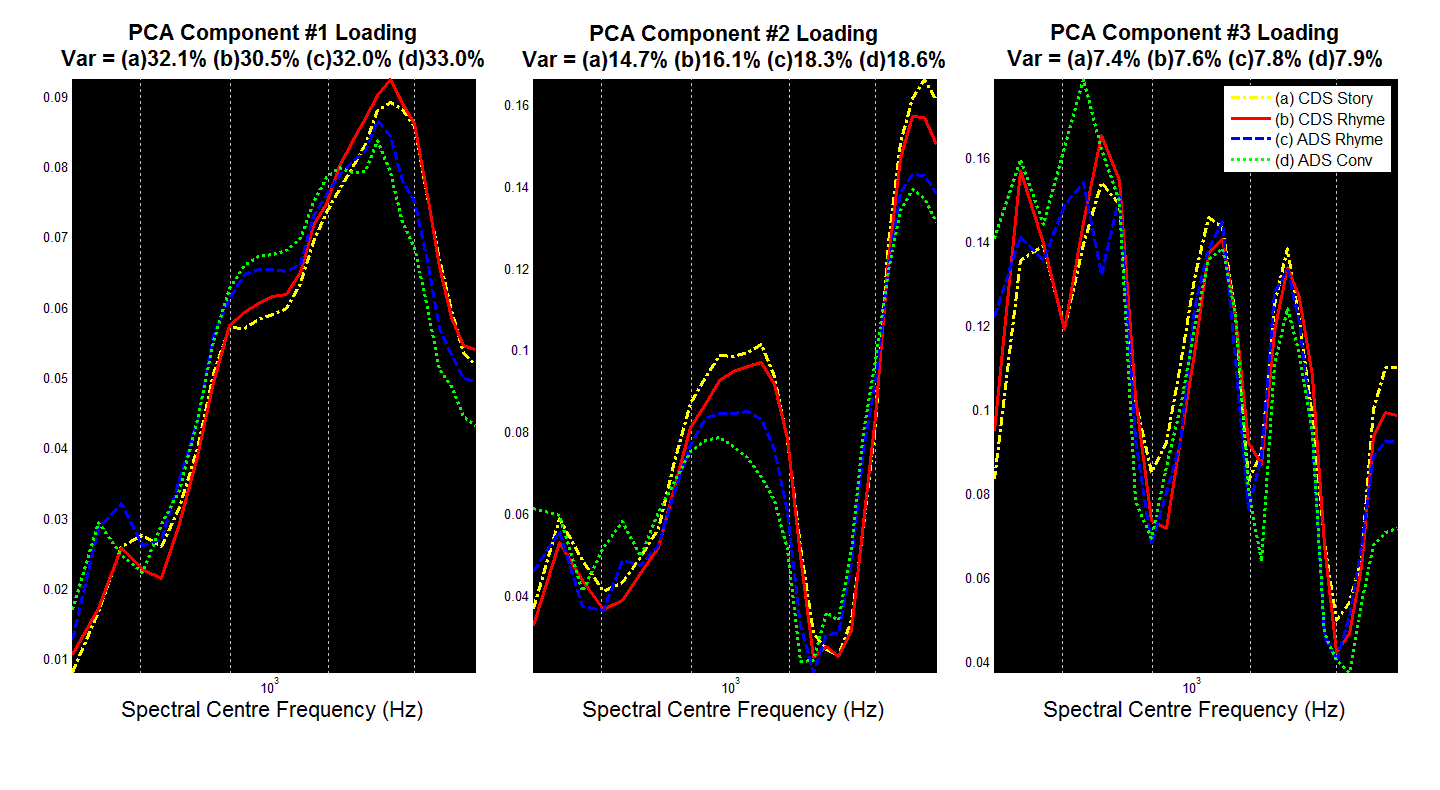


**b. Averaged Principal Component Loadings (Components 1-3)**

In the averaged loading patterns shown in Figure c, the trends differentiating CDS and ADS conditions are now clear. CDS conditions (yellow and red lines) loaded *more* *strongly* than ADS conditions (blue and green lines) at *higher* spectral frequencies (e.g. spectral bands 3 & 5), but loaded *less* *strongly* at *lower* spectral frequencies (e.g. spectral bands 1 & 2). It is also interesting to note that the overall order of the 4 speaking conditions was systematically preserved at both ends of the frequency spectrum, suggesting a parametric change between conditions. At high spectral frequencies, CDS Story loadings were the strongest, followed by CDS Rhyme, ADS Rhyme and ADS Conversation. At low spectral frequencies, this order was reversed, with ADS Conversation showing the strongest loadings, followed by ADS Rhyme, CDS Rhyme and CDS Story. However, the interpretation of these loading patterns requires careful consideration. Recall that in the previous section, the RMS power over the 28 spectral channels had been computed. This power spectrum is shown again in the bottom half of Figure c, where it can be directly compared with the PCA loading patterns. It may be observed that the higher component loading for CDS samples in spectral band 3 *is* accompanied by a relative boost in RMS power in this spectral band (grey arrow). Similarly, the drop in component loading for CDS samples in spectral band 1 is *also* accompanied by a drop in RMS power in this spectral band (grey arrow). However, the higher component loading for CDS samples in spectral band 5 is *not* accompanied by an increase in RMS power. Rather, in spectral band 5, the RMS power for CDS samples *decreases* relative to ADS samples (grey dotted arrow). Therefore, in CDS samples (Story & Rhyme), there is a relative 'boost' at middle spectral frequencies (~1200 Hs, spectral band 3) as compared to low spectral frequencies (<300 Hz, spectral band 1). This boost occurs for both RMS power (i.e. middle frequency sounds like vowels are louder), as well as for component loading strength (middle frequency modulation patterns are more similar). However, at very high spectral frequencies (>3900 Hz, spectral band 5), component loadings again increased in CDS, but this is now accompanied by a *drop* in RMS power (i.e. high frequency sounds like fricatives are *softer*).

*Figure c. (top) Mean rectified loadings averaged over PCA Components 1 to 3. The 4 speaking conditions are shown in different coloured lines. Vertical lines indicate the boundaries of the five spectral bands. (bottom) RMS power of the 28 spectral channels, replicated from Figure 7.2. Solid arrows indicate spectral regions where strength of component loading across conditions is positively correlated with RMS power. Dotted arrow indicates a spectral region where strength of component loading is negatively correlated with RMS power.*


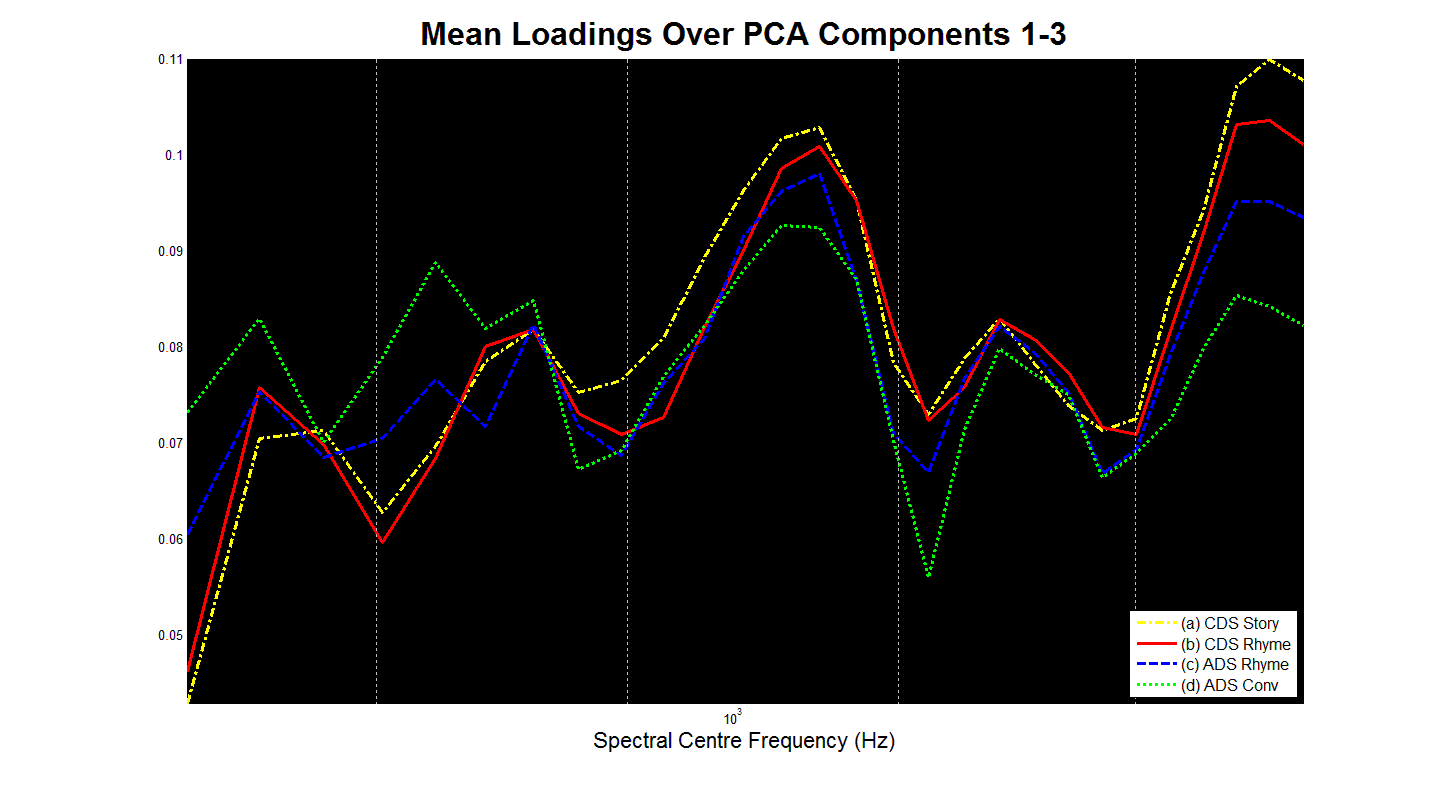

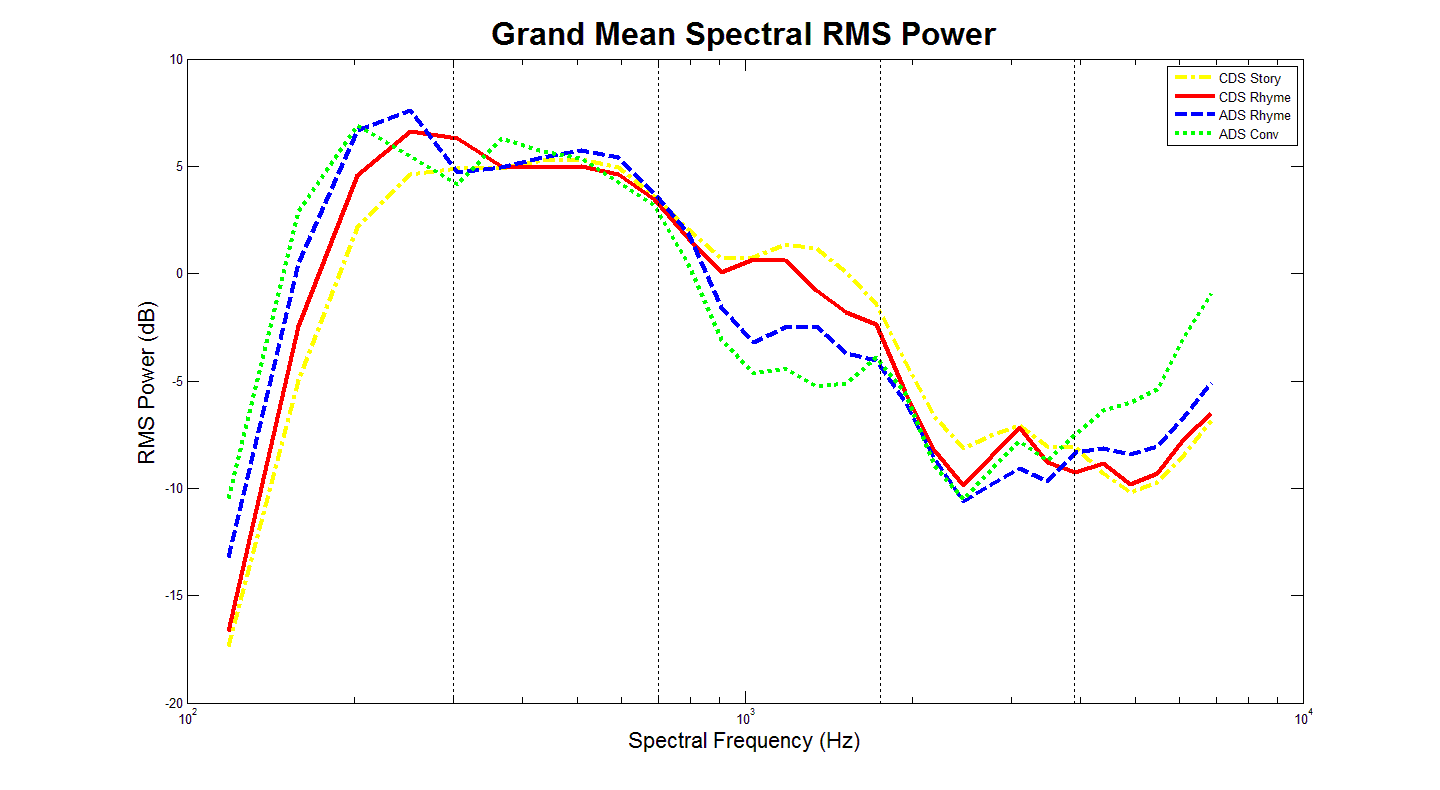


**Band 1 Band 2 Band 3 Band 4 Band 5**

**c. Interim Summary & Discussion of Spectral PCA Results**

Both child- and adult-directed speech shared the same fundamental spectral band structure of 5 major spectral bands. However, CDS speech samples showed a specific *boost* in spectral RMS power and PCA component loading strength at middle spectral frequencies around 1200 Hz, suggesting that vowel sounds may be particularly emphasised in CDS. This result is consistent with the finding that vowel sounds in child-directed speech are hyperarticulated, or more separated in formant space (Ratner, 1984; Burnham et al, 2002). Here, these results suggest that the vowel sounds in child-directed speech are also relatively louder, and more strongly co-modulated (i.e. contain similar patterns of modulation across the channels in each band). In contrast, CDS showed a relative *reduction* of RMS power for very high frequency (>3900 Hz) and very low frequency sounds (<300 Hz), even though high frequency sounds also showed increased co-modulation. This suggested that while high frequency sounds (like fricatives) were softer in CDS, they still contained strong and consistent modulation patterns.

Modulation Rate PCA

**a. RMS Power Across Modulation Channels**

Since the speech samples across the 4 conditions had the same 5-Band spectral structure, all the samples were filtered into 5 spectral bands, and Hilbert envelopes were extracted from each spectral band. These envelopes for each spectral band were then passed through a 24-channel modulation filterbank. Figure d shows the mean RMS power at each modulation channel (i.e. the modulation spectrum), for each of the 5 spectral bands (top plots), as well as the grand mean over the 5 spectral bands (bottom plot). Vertical lines indicate the boundaries between 'Stress', 'Syllable' and 'Phoneme' modulation bands. From visual inspection of the grand mean plot (bottom), CDS samples (red and yellow lines) appeared to have higher power in the Stress modulation band, and slightly lower power in Syllable and Phoneme modulation bands. This suggests that in child-directed speech, speakers tended to place relatively greater emphasis on the slower stress patterns, than on the faster syllable and phoneme-rate patterns.

*Figure d. Modulation spectrum for each spectral band (top) and averaged over all spectral bands (bottom). Coloured lines indicate the 4 speaking conditions. Shaded areas indicate the standard error of the mean for each condition. Vertical dotted lines indicate the boundaries of the 3 modulation rate bands.*

*
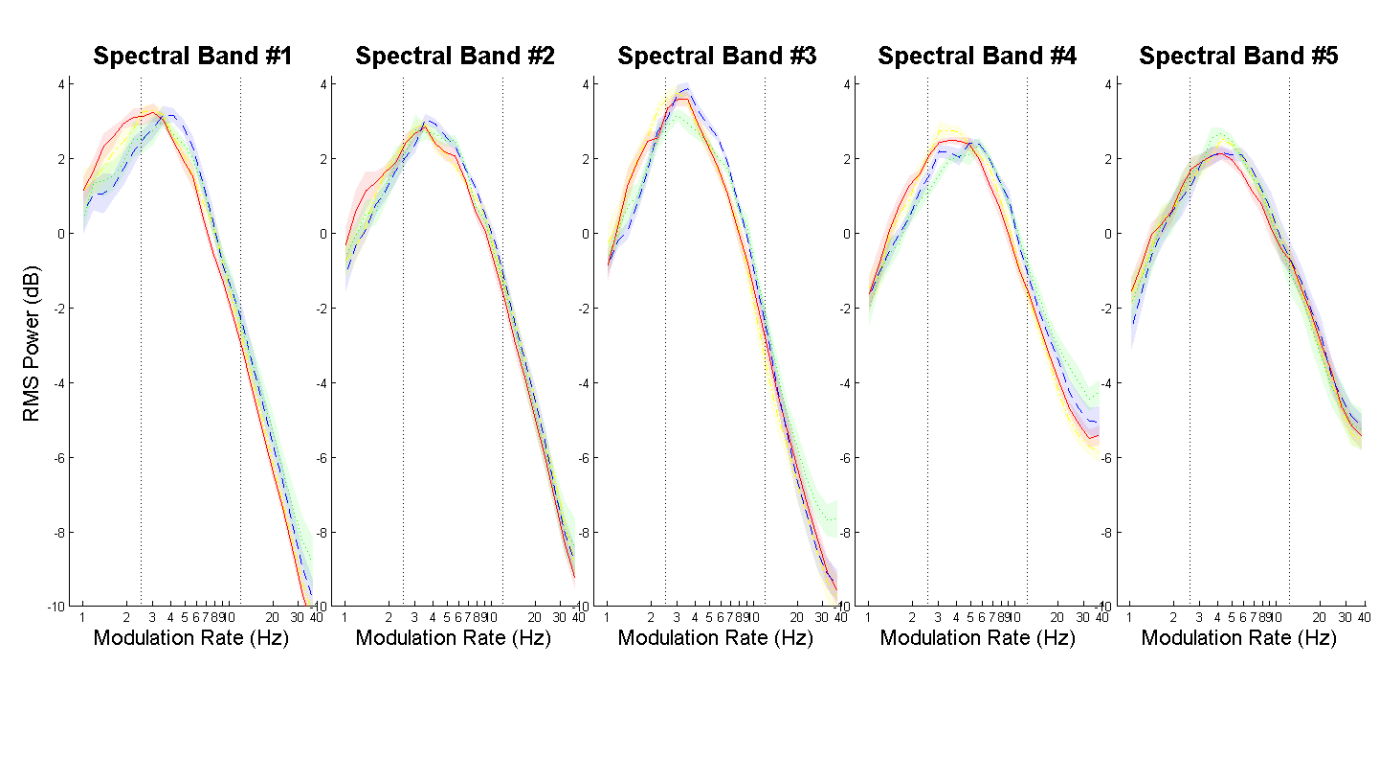
*

**Stress Syllable Phoneme**

*
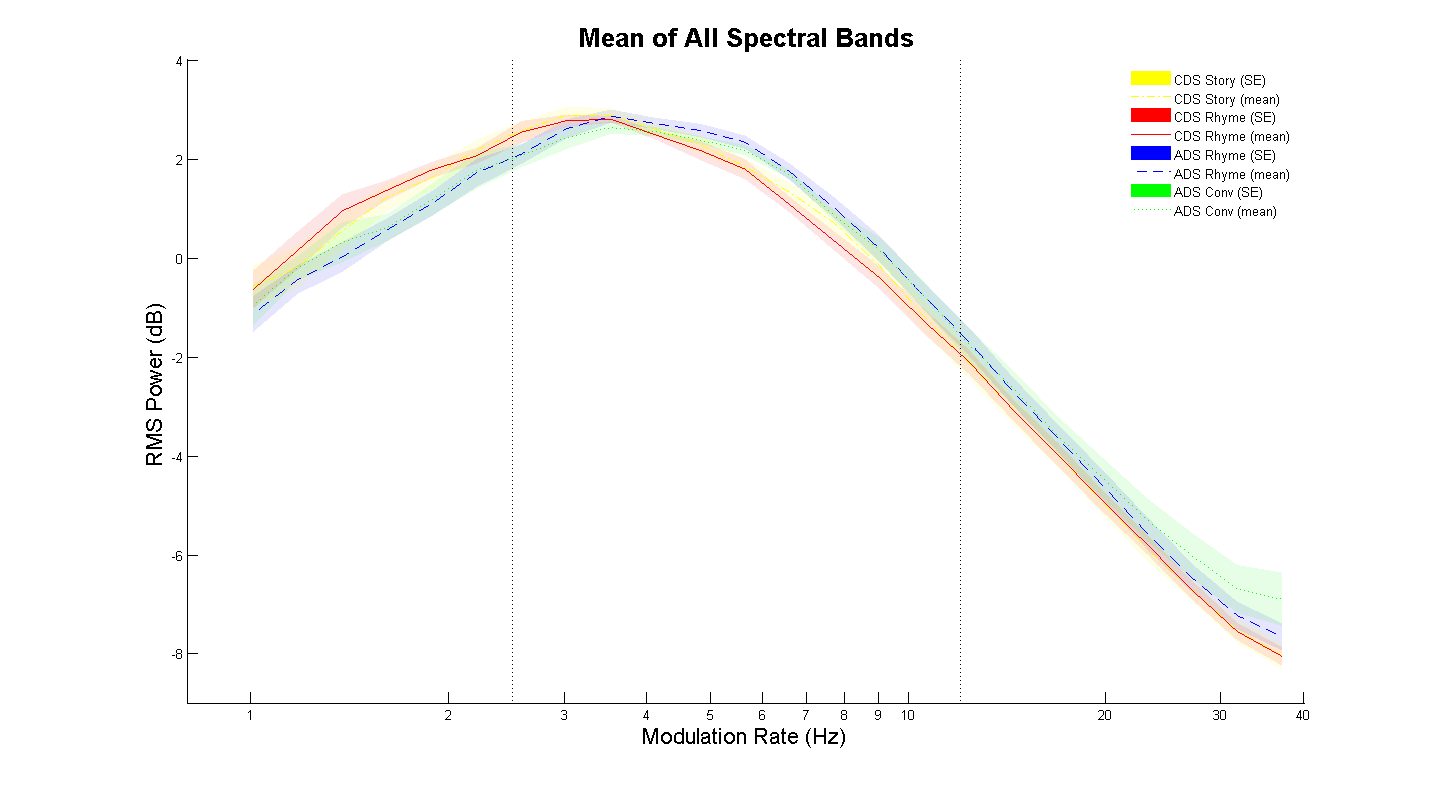
*

**b. Principal Component Loadings**

The modulation rate PCA analysis was carried out separately for each of the 5 spectral bands. Figure e shows the mean PCA loading patterns for the top 3 components, for each spectral band (top half of figure), as well as averaged over the 5 spectral bands (bottom half of figure). The top 3 principal components cumulatively accounted for almost 80% of the total variance in the samples. The variance accounted for by each principal component is indicated in the titles of the bottom plot of Figure e. In Figure e, the x-axis plots the centre frequency for each modulation channel and the y-axis plots the absolute (rectified) component loading, averaged across all samples and speakers. Each of the 4 speaking conditions is shown in a different colour. From visual inspection, the loading patterns were again highly similar across all 4 speaking conditions. The locations of major peaks and troughs (indicating modulation band boundaries) were also very similar across the four conditions, coinciding well with the band boundaries in the S-AMPH model. These S-AMPH modulation band boundaries are shown as vertical dotted lines. This suggests that the basic modulation band structure of the speech samples was again similar across the four speaking conditions, and agreed well with the band boundaries that had previously been identified.

*Figure e. Results of the Modulation Rate PCA Analysis. (top) Mean rectified loading patterns for each spectral band are shown in columns, PCA components 1 to 3 are shown in rows (bottom) Grand mean component loading patterns, averaged across spectral bands 1 to 5. The vertical lines in the plots indicate the boundaries between the 3 modulation rate bands at 2.5 Hz and 12 Hz. White arrows indicate inconsistencies in the order of loading strength across the 4 conditions, observed within the same modulation rate band.*

**Spectral Bands 1 to 5**

*
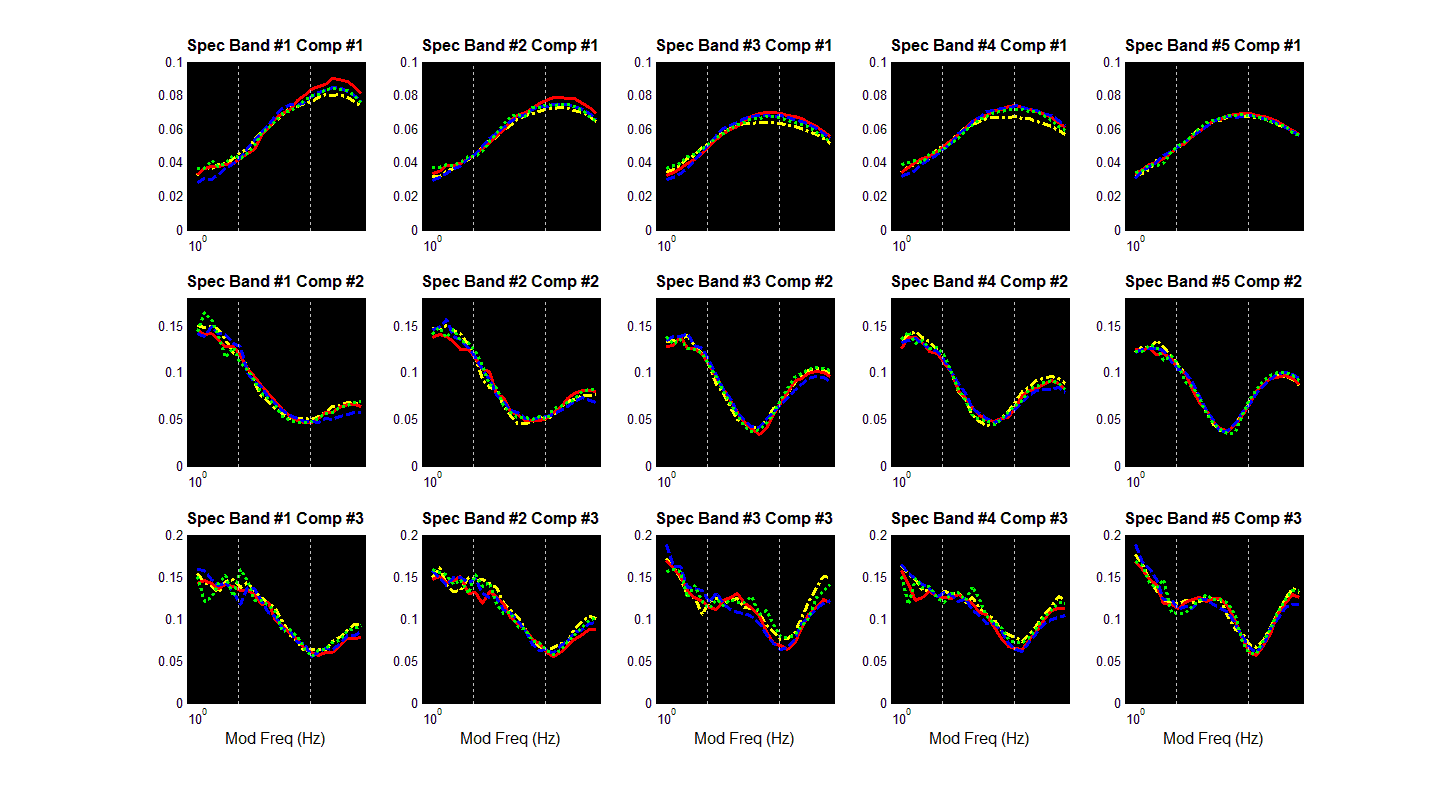
*
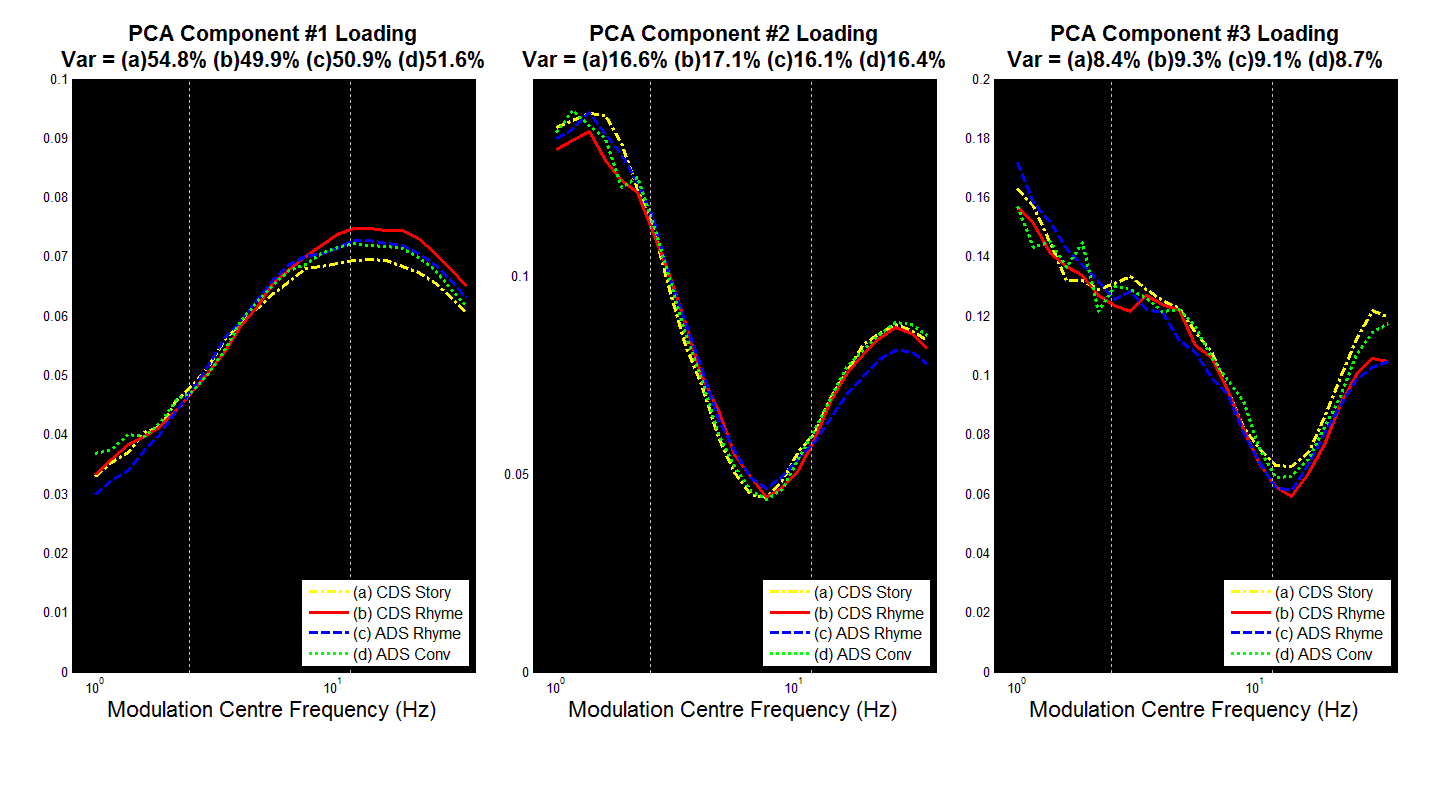


**PCA Components 1 to 3**
