# Supplementary material for: Acoustic-Emergent Phonology in the Amplitude Envelope of Child-Directed Speech
Source: PLoS One. 2015 Dec 7;10(12):e0144411. doi: 10.1371/journal.pone.0144411 (PMC4671555; doi:10.1371/journal.pone.0144411)
Supplement: S5 Appendix — (DOCX) [file pone.0144411.s005.docx]

**Detailed Breakdown of S-AMPH Performance for Each Phonological Level**

**Testing for Acoustic-Emergent Syllables.** The location of individual syllables in each utterance were automatically estimated by the S-AMPH model following the procedure outlined in the Methods (detection and selection of Syllable AM peaks). The model's success in syllable-finding was evaluated using signal detection theory. Table a shows the mean percentages of hits, misses, false alarms and correct rejections. The respective d-primes achieved by the S-AMPH model in syllable-finding for timed and untimed child-directed speech samples are also shown. The results for bi-syllable-footed sentences (i.e. trochees and iambs) and tri-syllable-footed sentences (i.e. dactyls and amphibrachs) are displayed in separate columns.

**Table a. Mean syllable-finding performance for timed and untimed speech samples.**

| SYLLABLE-FINDING | **Timed CDS**  (3 spkrs, 12 samples) | | **Untimed CDS**  (6 spkrs, 120 samples) | |
| --- | --- | --- | --- | --- |
|  | *Bi-Syll* | *Tri-Syll* | *Bi-Syll* | *Tri-Syll* |
| **Hits** | 98.2%  (1.8%) | 96.4%  (1.8%) | 85.8%  (2.4%) | 81.2%  (3.1%) |
|  | Average :  **97.3%** (2.7%) | | Average :  **83.5%** (1.8%) | |
| **Misses** | 1.8%  (1.8%) | 3.6%  (1.8%) | 14.2%  (2.4%) | 18.8%  (3.1%) |
|  | Average :  **2.7%** (2.7%) | | Average :  **16.5%** (1.8%) | |
| **False Alarms** | 0.5%  (0.9%) | 1.1%  (1.0%) | 18.0%  (6.3%) | 21.7%  (4.6%) |
|  | Average :  **0.8%** (1.5%) | | Average :  **19.9%** (3.5%) | |
| **Correct Rejections** | 99.5%  (0.9%) | 98.9%  (1.0%) | 82.0%  (6.3%) | 78.3%  (4.6%) |
|  | Average :  **99.2%** (1.5%) | | Average :  **80.1%** (3.5%) | |
| **d-prime**  (based on mean percentages shown above) | 4.66 | 4.09 | 1.99 | 1.67 |
|  | Average :  **4.33** | | Average :  **1.82** | |

Results are broken down by bi-syllable-footed (trochees/iambs) and tri-syllable-footed (dactyl/amphibrach) prosodic patterns respectively. The standard deviations over speakers are shown in brackets.

From inspection of Table a, it is apparent that the syllable-finding performance of the S-AMPH model was very good for both timed and untimed child-directed speech samples. The average accuracy of the model, computed as the average of hits and correct rejections, reached a remarkable 98.3% (d' = 4.33) for timed CDS. The accuracy for untimed CDS was lower at 81.8% (d' = 1.82), but still good. To assess whether there was a statistically-significant difference in syllable-finding performance between timed and untimed speech, a non-parametric Mann-Whitney U test was conducted, comparing the average d' scores for the 3 timed speakers to the 6 untimed speakers. The results of this test indicated that syllable-finding d-prime scores were indeed significantly higher for timed CDS than for untimed CDS (Z = 2.19, *p = .028*). We were also interested in whether the prosodic pattern of the sentences (i.e. bi-syllable or tri-syllable foot) had an effect on syllable-finding. According, we pooled the d' scores from timed and untimed speakers (i.e. 9 speakers in total), and conducted a non-parametric Wilcoxon matched pairs test comparing bi-syllable footed and tri-syllable footed sentences. The results of this second test indicated that there was a close to significant difference (Z = 1.82, *p = .068*), with bi-syllable footed sentences (i.e. trochees and iambs) yielding marginally higher d' scores than tri-syllable footed sentences (i.e. dactyls and amphibrachs). As might be expected, finding the syllables in a nursery rhyme with dactyl structure like ‘*Pussycat Pussycat Where Have You Been’* was more challenging for the model than finding the syllables in a nursery rhyme with trochaic structure like ‘*Mary Mary Quite Contrary’*.

**Testing for Acoustic-Emergent Onset-Rime Units.** The location of onset-rime divisions within each syllable in each utterance was estimated by the S-AMPH model following the procedure outlined in the Methods section. The model's success in onset-rime detection was evaluated using signal detection theory. Table b shows the mean percentages of hits, misses, false alarms, correct rejections and respective d-primes achieved by the S-AMPH model for timed and untimed child-directed speech samples. The results for bi-syllable-footed sentences (i.e. trochees and iambs) and tri-syllable-footed sentences (i.e. dactyls and amphibrachs) are displayed in separate columns.

**Table b. Mean onset-rime division detection performance for timed and untimed speech samples.**

| ONSET-RIME DIVISION-DETECTION | **Timed CDS**  (3 spkrs, 12 samples) | | **Untimed CDS**  (6 spkrs, 120 samples) | |
| --- | --- | --- | --- | --- |
|  | *Bi-Syll* | *Tri-Syll* | *Bi-Syll* | *Tri-Syll* |
| **Hits** | 84.5%  (4.5%) | 89.3%  (3.6%) | 78.2%  (2.8%) | 71.6%  (6.6%) |
|  | Average :  **86.9%** (2.2%) | | Average :  **74.9%** (4.2%) | |
| **Misses** | 15.5%  (4.5%) | 10.7%  (3.6%) | 21.8%  (2.8%) | 28.4%  (6.6%) |
|  | Average :  **13.1%** (2.2%) | | Average :  **25.1%** (4.2%) | |
| **False Alarms** | 6.1%  (1.7%) | 4.0%  (1.8%) | 18.4%  (3.5%) | 20.9%  (4.5%) |
|  | Average :  **5.1%** (1.4%) | | Average :  **19.6%** (3.0%) | |
| **Correct Rejections** | 93.9%  (1.7%) | 96.0%  (1.8%) | 81.6%  (3.5%) | 79.1%  (4.5%) |
|  | Average :  **94.9%** (1.4%) | | Average :  **80.4%** (3.0%) | |
| **d-prime**  (based on mean percentages shown above) | 2.56 | 2.99 | 1.68 | 1.38 |
|  | Average :  **2.76** | | Average :  **1.53** | |

Results are broken down by bi-syllable-footed (trochees/iambs) and tri-syllable-footed (dactyl/amphibrach) prosodic patterns respectively. The standard deviations over speakers are shown in brackets.

The onset-rime detection performance was good for both timed and untimed child-directed speech samples. The average accuracy of the S-AMPH model, computed as the mean of the hit and correct rejection percentages, was 90.9% (d' = 2.76) for timed CDS and 77.6% (d' = 1.53) for untimed CDS. To assess whether onset-rime detection performance was significantly different for timed and untimed speech samples, a non-parametric Mann-Whitney U test was conducted, comparing the average d' scores for the 3 timed speakers to the 6 untimed speakers. The results of this test indicated that onset-rime detection d-prime scores were indeed significantly higher for timed CDS than for untimed CDS (Z = 2.19, *p = .028*). Next, we assessed whether the prosodic pattern of the sentences (i.e. bi-syllable or tri-syllable foot) had an effect on onset-rime detection. According, we pooled the d' scores from timed and untimed speakers (i.e. 9 speakers in total), and conducted a non-parametric Wilcoxon matched pairs test comparing bi-syllable footed and tri-syllable footed sentences. The results of this test indicated that there were *no* significant differences in onset-rime detection between bi-syllable footed and tri-syllable footed sentences (Z = 0.65, *p = .51*).

**Testing for Acoustic-Emergent Prosodic Strength.** Figure a plots the trade-off between the hit rate and the false alarm rate for different Prosodic Strength Measure (PSM) threshold values as an ROC (receiver operating characteristic) curve, computed for the untimed CDS corpus. As may be observed from Figure 5, the d-prime score and accuracy of the model was poor for a PSM threshold of 0.1 (d'~1.0, Accuracy ~64%), but improved for thresholds of 0.2 and above (d'~1.2, Accuracy~72%). Table c shows a breakdown of the results obtained when a PSM threshold of 0.2 was used for the untimed CDS corpus, and these data are used in subsequent statistical analyses.

**
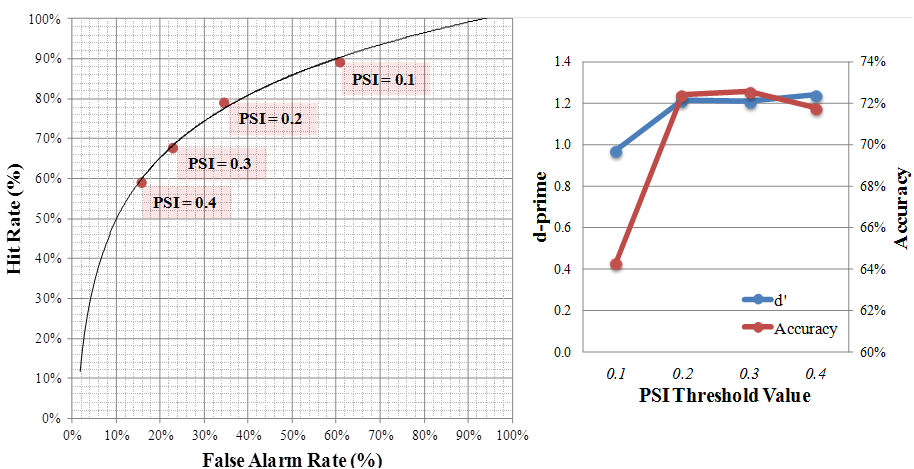
**

**Fig. a. Hit rates and d'prime values for prosodic stress detection.** (left) ROC curve for S-AMPH prosodic stress detection using different PSM threshold values, for the untimed CDS sample. The actual computed hit rates (y-axis) and false alarm rates (x-axis) for 4 PSM threshold values are shown as red dots. The solid black curve indicates the logarithmic line of best fit through these points.(right) Equivalent d-prime scores (left y-axis) and Accuracy rates (right y-axis) for each PSM threshold value.

Table c shows the mean percentages of hits, misses, false alarms and correct rejections, and the respective d-primes achieved by the S-AMPH model in stress detection for timed (PSM threshold = 0.4) and untimed (PSM threshold = 0.2) child-directed speech samples. The results for bi-syllable-footed sentences (i.e. trochees and iambs) and tri-syllable-footed sentences (i.e. dactyls and amphibrachs) are shown in separate columns.

**Table c. Mean stress detection performance for timed and untimed speech samples.**

| STRESS-DETECTION | **Timed CDS**  (3 spkrs, 12 samples;  *PSM threshold = 0.4*) | | **Untimed CDS**  (6 spkrs, 120 samples;  *PSM threshold = 0.2*) | |
| --- | --- | --- | --- | --- |
|  | *Bi-Syll* | *Tri-Syll* | *Bi-Syll* | *Tri-Syll* |
| **Hits** | 96.4%  (3.6%) | 100.0%  (0.0%) | 78.8%  (3.4%) | 79.6%  (4.9%) |
|  | Average :  **98.2%** (1.8%) | | Average :  **79.2%** (3.6%) | |
| **Misses** | 3.6%  (3.6%) | 0.0%  (1.8%) | 21.2%  (3.4%) | 20.4%  (4.9%) |
|  | Average :  **1.8%** (1.8%) | | Average :  **20.8%** (3.6%) | |
| **False Alarms** | 3.9%  (0.3%) | 13.6%  (1.3%) | 33.3%  (7.6%) | 35.6%  (4.7%) |
|  | Average :  **8.7%** (0.8%) | | Average :  **34.4%** (4.6%) | |
| **Correct Rejections** | 96.1%  (0.3%) | 86.4%  (1.3%) | 66.7%  (7.6%) | 64.4%  (4.7%) |
|  | Average :  **91.3%** (0.8%) | | Average :  **65.6%** (4.6%) | |
| **d-prime**  (based on mean percentages shown above) | 3.57 | 3.43 | 1.23 | 1.20 |
|  | Average :  **3.46** | | Average :  **1.21** | |

Results are broken down by bi-syllable-footed (trochees/iambs) and tri-syllable-footed (dactyl/amphibrach) prosodic patterns respectively. The standard deviations over speakers are shown in brackets.

The stress-detection performance was good for both timed and untimed child-directed speech samples. The average accuracy of the S-AMPH model, computed as the mean of the hit and correct rejection percentages, was 94.8% (d' = 3.46) for timed CDS and 72.4% (d' = 1.21) for untimed CDS. To assess whether stress-detection performance was significantly different for timed and untimed speech samples, a non-parametric Mann-Whitney U test was conducted, comparing the average d' scores for the 3 timed speakers to the 6 untimed speakers. The results of this test indicated that stress-detection d-prime scores were indeed significantly higher for timed CDS than for untimed CDS (Z = 2.19, *p = .028*). Next, we assessed whether the prosodic pattern of the sentences (i.e. bi-syllable or tri-syllable foot) had an effect on stress-detection. According, we pooled the d' scores from timed and untimed speakers (i.e. 9 speakers in total), and conducted a non-parametric Wilcoxon matched pairs test comparing bi-syllable footed and tri-syllable footed sentences. The results of this test indicated that there were *no* significant differences in stress detection between bi-syllable footed and tri-syllable footed sentences (Z = 0.65, *p = .51*).
