# Supplementary material for: Acoustic-Emergent Phonology in the Amplitude Envelope of Child-Directed Speech
Source: PLoS One. 2015 Dec 7;10(12):e0144411. doi: 10.1371/journal.pone.0144411 (PMC4671555; doi:10.1371/journal.pone.0144411)
Supplement: S1 Code — (DOCX) [file pone.0144411.s006.docx]

**Matlab S-AMPH Code and Functions Used**

**a. Matlab code to extract S-AMPH Hierarchy**

*('SAMPH_1_Extract5x3_single.m', 'MFB_coeffs.m')*

Also available at : http://figshare.com/articles/SAMPH_CDS/1318572

DOI : 10.6084/m9.figshare.1318572

___________________________________________________________________________

function [Y,tA,CF,CF_MFB,NSamp] = SAMPH_1_Extract5x3_single(FileName,FSamp);

% DESCRIPTION :

% Produces downsampled 5x3 spectro-temporal envelopes of audio (.wav) files. The 5 spectral

% channels span 100-7250 Hz, the 3 modulation rate bands span 0.9-40 Hz (Stress, Syllable, Phoneme).

% INPUT VARIABLES :

% FileName = Name of file to process e.g. 'E1_Mary.wav'

% FSamp = Sampling frequency of wav files, in Hz. Only 44100 or 16000 accepted.

% OUTPUT VARIABLES :

% Y = Cell array of 5-freq channel filtered signals (no demodulation), single point precision and downsampled to either 7350 Hz (for 44.1 kHz) or 8000 Hz (for 16 kHz)

% tA = Cell array of spectro-temporal Hilbert envelopes, in the form L x 3 (mod rate : Stress, Syllable, Phoneme) x 5 (spectral band : low to high)

% CF = Arithmetic centre frequencies of the 5 spectral channels

% CF_MFB = Arithmetic centre frequencies of the 3 modulation rate channels (N.B. It may be more appropriate to report geometric CFs of 1.5 Hz, 5.5 Hz & 21.9 Hz)

% NSamp = Final downsampled frequency of the spectro-temporal envelopes (1050 or 1000 Hz)

% EXAMPLE OF USE :

% [Y,tA,CF,CF_MFB,NSamp] = SAMPH_1_Extract5x3_single('E1_Mary.wav'',44100)

%-------------------------------------------------------------------------%

%%% Downsample envelope

if FSamp == 44100;

ds1 = 3; %% iSamp = 14.7khz

ds2 = 14; %% NSamp = 1050 Hz

elseif FSamp == 16000;

ds1 = 1; %% iSamp = 16000

ds2 = 16; %% NSamp = 1000;

else

display('Incompatible sampling rate, please use 44100 or 16000 only')

end

iSamp = FSamp/ds1;

NSamp = iSamp/ds2;

%%% Set filterbank edges

N = 5;

edges = [10;100;300;700;1750;3900;7250];

[CF, bpfs] = MFB_coeffs(edges,iSamp,1);

CF = CF(2:end);

M = 3;

Fcor = [0.1; 0.9; 2.5; 12; 40];

[CF_MFB,MFB_bpfs] = MFB_coeffs(Fcor,NSamp,2);

sil = zeros(length(MFB_bpfs)/2,1);

CF_MFB = CF_MFB(2:end);

y = wavread(FileName);

y = y(1:ds1:end);

%%%% Extract 5 SPECTRAL BANDS

yfil = zeros(length(y),N);

for n = 2:N+1

l_fil = bpfs(1,n);

fil_n = bpfs(2:1+l_fil,n);

nshift = floor(l_fil/2);

yfil = filter(fil_n, 1, y);

yfil = sample_advance(yfil, nshift, 1e-7);

ychan(:,n-1) = single(yfil(1:2:end));

Ahil = abs(hilbert(yfil));

Ahil = Ahil(1:ds2:end);

clear yfil

%%%% Extract 3 MODULATION RATE BANDS

Ahil = [sil; Ahil; sil];

Atmp = zeros(length(Ahil),1);

Afil = zeros(length(Ahil),M);

for m = 2:M+1

bpf_len = MFB_bpfs(1,m);

bpf_chan = MFB_bpfs(2:bpf_len+1,m);

Atmp = filter(bpf_chan, 1, Ahil);

dly_shift = floor(bpf_len/2);

len_A = length(Atmp) - dly_shift;

Afil(1:len_A,m-1) = Atmp(1+dly_shift:length(Atmp));

Afil(1+len_A:length(Atmp),m-1) = 0;

clear Atmp

Atmp = zeros(length(Ahil),1);

end

A(:,:,n-1) = Afil(length(sil)+1:end-length(sil),:);

end

Y = ychan;

tA = A;

clear Afil

clear Atmp

clear A

clear ychan

__________________________________________________________________________

% Copyright (C) M.A. Stone 2001-2013, under funding from received from MRC (UK)

function [CF, anlys_bpfs] = MFB_coeffs(edges, FSamp, diagnostic_fig);

%%%% 'edges' contains the n+1 edges to the channels,

%%%% 'FSamp' sampling frequency in Hz

%%%% 'diagnostic_fig' if non-zero prints diagnostics from filter design and plots response to the same figure number as "diagnostic_fig"

%%%% returns : 'CF' : centre frequencies of band-pass filters (arithmetic mean of adjacent edges)

%%%% 'anlys_bpfs' FIR filter arrrays (part-filled): first term is number of taps, followed by the taps.

%%%%%%%%%%%%%%%%%%%%%%%%%%%%%%%%%%%%%%%%%%%%%%%%%%%%%%%%%%%%%%%%%%%%%%%%%%%%%%%%%%%%%%%%%%%

%%%% to generate bandpass filters for analysis/synthesis, need some magic numbers to define

%%%% transition widths/time domain impulse response. Originally designed to produce audiotry filterbank

%%%% suitable for human auditory experiemnts. Modified for V Leong to be suitable for modulation filterbank

%% esp when using lower sampling rates . Parameters chosen so that no long-time impulse response filters

%%%% generated. Also set minimum number of taps to ensure that tails fall to < -65 dB

%%%% Also adjust transition width so that for low number of channels, where spacing is typically high,

%%%% it still chooses moderately steep filters to get good channel separation

%%%% In order to tame the filter tails: in lpf design use kaiser with beta=7.4,

%%%% Since FIR widths change, we need to time-align the envelopes extracted, as well as

%%%% the noise samples, extracted by the same filters . Done outside of this code

nchans = length(edges)-1; %% number of processing channels

firlen0 = 2*floor(1.4*FSamp/1000); %%% minimum length for FIR filter, suitable for high frequency channels. Lower channels have longer span

max_mult = 6; %%%% controls maximum fir size likely for low-freq channels

DIAGNOSTIC = diagnostic_fig; %% if set, prints filterbank responses to different windows

%% 'qnorm', along with 'q', and cf spacing adjusts transition widths of filters so that they appear nearly similar across filterbank, when viewed on logarithmic frequency scale.

qnorm = 5; %%%% magic number of about 5: [q] and [qnorm] are not true filter "quality factors" as in the text books, but essentially perform a similar function, steepness of transition.

bp_lf = edges(1:nchans); %%% lower corner frequencies for channel splits

bp_hf = edges(2:nchans+1); %%% upper corner frequencies ditto

CF= .5*(bp_hf + bp_lf);%%% centre frequencies/crossovers

%%%%%% 9-10-01 shape of transition is dependent on difference between the adjacent cfs.

delta_cf = CF(2:nchans) - CF(1:nchans-1);

%%%% measure of steepness, is 'q' around transition

q = .5*(CF(2:nchans) + CF(1:nchans-1))./(CF(2:nchans) - CF(1:nchans-1));

%%% effectively broaden transitions for high nchans, tighten for low.....

delta_cf = delta_cf.*((q.^.5)/qnorm); %%% stable Oct2005

anlys_bpfs = zeros((2*max_mult*firlen0+2),nchans); %%% set up maximum storage for bpfs, pre-calculated

%%%%%%%% generate bpfs in ascending order: low-pass seciton of channel m becomse high-pass section of channel m+1

for ix = 1:nchans %%% design firs, gradually increase window to reduce tails

%%%%% design channel filter in two stages, depending on adjacent channels, high pass first then low-pass

if ix ~= 1 %% every channnel other than first....... hpf = 1-lpf for centre term, or -lpf otherwise

mid_lpf = floor(length(lpf_chan)/2) +1; %%%%%% index of middle position of lpf

hpf_chan = -lpf_chan; %%%% high-pass is complementary of low-pass from previous channel

hpf_chan(mid_lpf) = 1 + hpf_chan(mid_lpf);

else %% chan 1 high-pass FIR would be very long, and little info there in real signals, so cheapskate on design

hpf_chan = 1; %%%% special case at start

end

if ix < nchans %%% firlen adapts to transition width, last channel is irrelevant

%%%%% must ALWAYS end up EVEN, so fir1() turns it ODD

firlen = max(firlen0, max_mult*floor(firlen0 * 100/delta_cf(ix) ));

end

%% use MATLAB fir1(N, fc, window) function to design low-pass stage. Window is used to tame tails of design, but broadens transition width between filters

lpf_chan = fir1(firlen, bp_hf(ix)/(FSamp/2),kaiser(firlen+1,7.4)); %% tames tails: to (-10*beta in dB) : 7.4 post Jan09

bpf_len = length(hpf_chan) + length(lpf_chan) - 1;

bpf_chan = zeros(bpf_len,1);

bpf_chan(1:length(hpf_chan)) = hpf_chan; %%%% copy in

bpf_chan = filter(lpf_chan,1,bpf_chan); %%% and convolve two halves of filter to make one

if DIAGNOSTIC %%% FREQUENCY RESPONSE OF ANALYSIS BAND-PASS FILTER DESIGN

figure(DIAGNOSTIC); [hz fz] = freqz(bpf_chan, 1, 4096, FSamp); %% autoscale freq range according to 'edges'

semilogx(fz, 20*log10(abs(hz)),'r'); hold on ; set(gca,'xlim',[0.7*edges(1) 1.414*edges(end)],'ylim',[-75 3]); grid on ;

title(['qnorm = ',num2str(qnorm)])

end

%%% no need to ensure 0dB gain at centre otherwise flat recombination does not work, especially with round-topped filters

anlys_bpfs(1,ix) = bpf_len; %% save filter size and.....

anlys_bpfs(2:bpf_len+1,ix) = bpf_chan; %%%%% .......filter for later

end

if DIAGNOSTIC %% set figure to be overwritten next time this figure() is used

figure(DIAGNOSTIC); hold off;

end

__________________________________________________________________________

**b. Key Matlab functions and computations used in analysis**

- PCA : In-house function developed by Dr Richard Turner (*http://cbl.eng.cam.ac.uk/Public/Turner/Turner*).
- 'findpeaks.m' : Function from Signal Processing Toolbox that identifies local maxima (peaks) of the input data. A local peak is a data sample that is larger than either of its two neighbouring samples. Three further input arguments, 'minpeakheight', 'threshold' and 'minpeakdistance', can be set to calibrate the sensitivity of the peak-finding process. This function was used to identify local maxima and minima in the PCA loading patterns and also to identify Syllable AM peaks during model evaluation.
- 'fft.m' : discrete Fourier transform, computed with a fast Fourier transform algorithm.

The function Y = fft(x) is implemented for a vector of length *N* as follows :


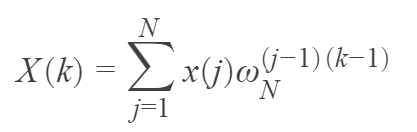


where


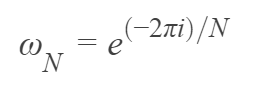


is an *N*th root of unity.

This function was used to determine a representative syllable period for each speech sample in order to determine the minimum peak distance to be used during Syllable AM peak finding.
